# Computationally-Guided Development of a Stromal Inflammation Histologic Biomarker in Lung Squamous Cell Carcinoma

**DOI:** 10.1038/s41598-018-22254-4

**Published:** 2018-03-02

**Authors:** Daniel Xia, Ruben Casanova, Devayani Machiraju, Trevor D. McKee, Walter Weder, Andrew H. Beck, Alex Soltermann

**Affiliations:** 1 Department of Pathology, Brigham and Women’s Hospital, Boston, MA USA; 2 Institute of Pathology, University Hospital Zurich, Zurich, Switzerland; 3 Department of Pathology, Beth Israel Deaconess Medical Center and Harvard Medical School Boston, Boston, MA USA; 4 Division of Thoracic Surgery, University Hospital Zurich, Zurich, Switzerland; 5 Department of Pathology, Massachusetts General Hospital, Boston, MA USA; 6 Department of Pathology, University Health Network, Toronto, ON Canada; 7 STTARR Innovation Centre, University Health Network, Toronto, ON Canada

## Abstract

The goal of this study is to use computational pathology to help guide the development of human-based prognostic H&E biomarker(s) suitable for research and potential clinical use in lung squamous cell carcinoma (SCC). We started with high-throughput computational image analysis with tissue microarrays (TMAs) to screen for histologic features associated with patient overall survival, and found that features related to stromal inflammation were the most strongly prognostic. Based on this, we developed an H&E stromal inflammation (SI) score. The prognostic value of the SI score was validated by two blinded human observers on two large cohorts from a single institution. The SI score was found to be reproducible on TMAs (Spearman rho = 0.88 between the two observers), and highly prognostic (e.g. hazard ratio = 0.32; 95% confidence interval: 0.19–0.54; p-value = 2.5 × 10^−5^ in multivariate analyses), particularly in comparison to established histologic biomarkers. Guided by downstream molecular/biomarker correlation studies starting with TCGA cases, we investigated the hypothesis that epithelial PD-L1 expression modified the prognostic value of SI. Our research demonstrates that computational pathology can be an efficient hypothesis generator for human pathology research, and support the histologic evaluation of SI as a prognostic biomarker in lung SCCs.

## Introduction

Lung cancer is the leading cause of cancer-related deaths in the United States, with an estimated 220,000 new cases and 150,000 deaths each year^1^. Approximately 85% of all lung cancers are non-small cell lung cancers (NSCLCs), and squamous cell carcinoma (SCC) is the second most common type. In contrast to tumor grading for lung adenocarcinomas^2^ (the most common type of NSCLC), there is currently no universally accepted method for the prognostic stratification of lung SCCs by histology. Traditional histologic grading of lung SCC includes the assessment of the extent tumor epithelial keratinization, but the prognostic relevance of this is questionable^3^. In view of these considerations, we selected lung SCC as a candidate for high-throughput computational histomorphometric discovery.

Computational analysis of digital pathology images is a relatively new tool for researchers in pathology, and offers opportunities for the discovery of morphologic features predictive of patient outcomes, including features not assessed in traditional clinical practice. As an illustrative example, in a previous study, we developed a machine-learning-based model called C-Path (or Computational Pathologist) to predict breast cancer patient survival from quantitative analyses of the patients’ digital pathology images^4^. The computational 5YS (5 year survival) histologic prognostic score developed from this approach was better at predicting overall survival than the conventional grading of breast cancers in a validation cohort. Interestingly, the 5YS score was based on histologic features from both the neoplastic cancer epithelia (which is the traditional focus for pathologists) and the non-neoplastic cancer stroma (which is not traditionally commonly assessed), emphasizing the importance of the latter for tumor biology. A major current limitation to the clinical translation of this form of computational analysis is that it is not yet widely available or validated for routine clinical use.

Conceptually, human pathology and computational image analysis have different relative strengths and weaknesses, and the two approaches can (at times) complement each other (see Table 1). The relative strengths of computational pathology include the ability to objectively assess and quantify a large number of features (far more than would be possible for a human researcher) in a systematic and high-throughput fashion. The relative strengths of human pathology include the ability of pathologists to integrate highly complex histologic and clinical data in the appropriate contexts (tasks not yet mastered by algorithms), as well as the wide-availability of routine hematoxylin and eosin (H&E) stained slides. In view of these considerations, we reasoned that one particularly practical combination of the two approaches would be to employ digital image analysis as a high-throughput histologic screening tool (analogous to screens in genomics), and then to translate the findings from the analysis into real parameters for scoring by human pathologists on H&E staining. It is expected that in comparison to a computer-based score (e.g. the 5YS score), a straightforward human-based H&E biomarker could have more immediate clinical impact.

**Table 1:** A comparison of the relative strengths and weaknesses of human pathology and computational histologic image analysis. The following is intended to illustrate the rational basis for the current project (summarized in Fig. 1). Considerations 1–2 favor the application of computational pathology as a screening tool for hypothesis generation. Considerations 4–6 outline some of the reasons for why human pathologists are arguably more suited for current clinical practice. Consideration 3 is a relative advantage of computer-based image analysis, but the concordance between and within observers for human-based histologic biomarkers vary widely. In the case of the SI score, the intra- and inter-observer concordance was found to be high, at least on tissue microarrays (see Results and Supplementary Figure 1).

	Computer-based histologic image analysis	Human-based histology
1. Number of features assessed at one time	Strength: Can be a relatively large number (e.g. > 1000)	Weakness: Usually a relatively small number (e.g. < 10)
2. Bias in selecting features	Strength: No inherent bias in favor of either neoplastic or non-neoplastic tissue	Weakness: Potential bias towards epithelial biomarkers, since the classification of neoplastic tissue is a traditional focus of oncological pathology
3. Intra- and inter-observer variability	Strength: The same algorithm analyzing the same digital image should give the same result every time	Weakness: Reproducibility can be an issue for human pathologists, but this depends greatly on the feature assessed
4. Use in routine clinical practice	Weakness: Not currently	Strength: Human-based histopathology is the current gold-standard in clinical practice
5. Versatility across different clinical and pathologic settings	Weakness: Algorithms developed for a specific application may not work well in other settings, e.g. an algorithm trained only on breast cancer examples may not interpret foci of adjacent benign breast lobules appropriately, if the system has not been trained to identify normal breast tissue.	Strength: Human pathologists are currently more versatile than computer algorithms trained for specific applications. This represents a distinct advantage in the practice of general surgical pathology, which depends heavily on a very wide breadth of knowledge of different tissue types and pathological processes.
6. Availability of method	Weakness: Currently not widely available; requires specialized software and hardware	Strength: Currently widely available; can be used by people in any research or clinical laboratory

**Figure 1:**
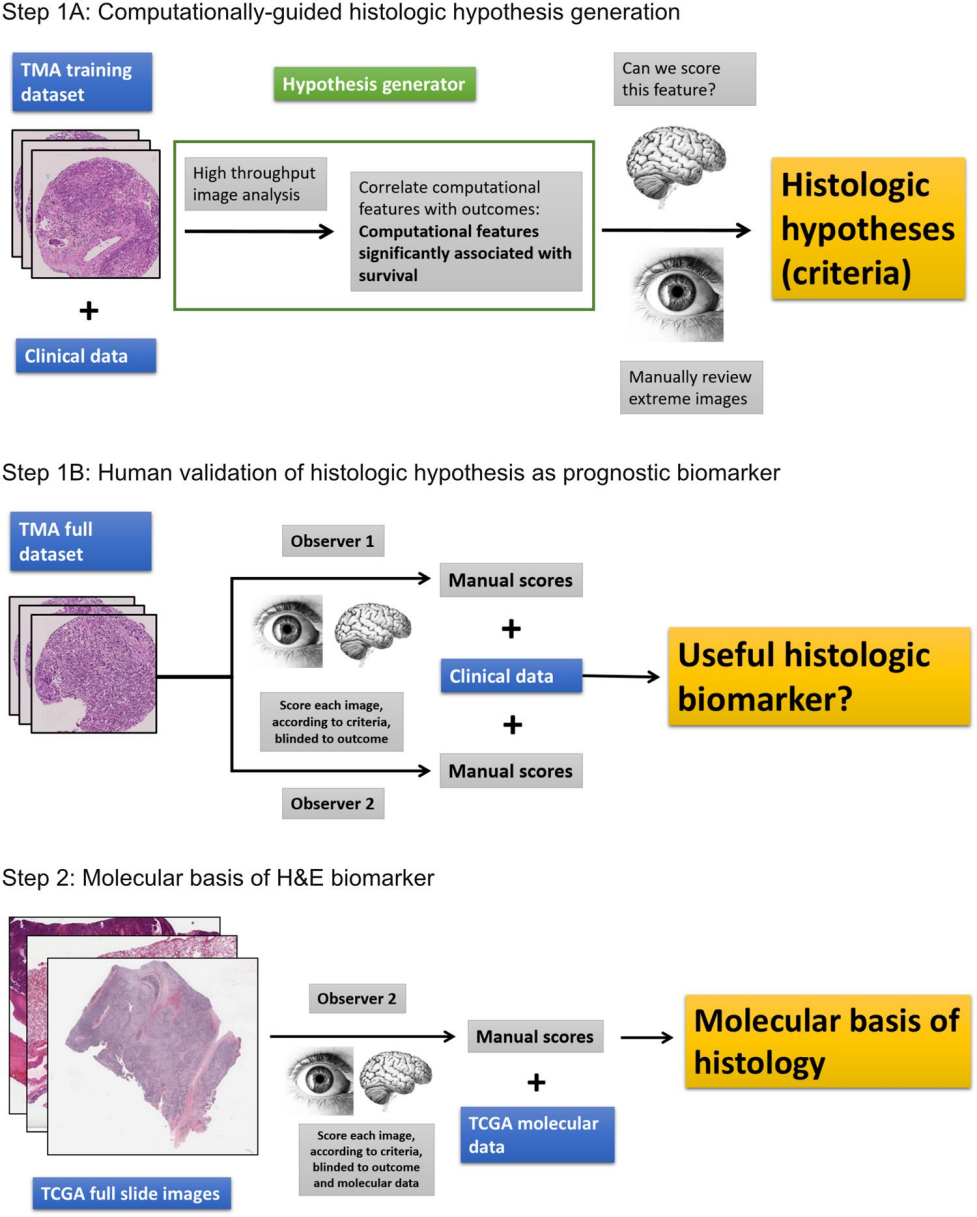
Study Overview. Note: the image of the human brain is in the Public Domain and was obtained from the Wikimedia Commons (https://upload.wikimedia.org/wikipedia/commons/8/88/PSM_V46_D167_Outer_surface_of_the_human_brain.jpg). The image of the human eye was modified from the original image (“A blue iris. A human eye.” created by user 8thstar at the English language Wikipedia) obtained from the Wikimedia Commons (https://upload.wikimedia.org/ wikipedia/commons/8/84/A_blue_eye.jpg) under the Creative Commons (CC) BY-SA 3.0 license (https://creativecommons.org/licenses/by-sa/3.0/). According to the terms of this license, this particular Fig. 1 is also distributed under CC BY-SA 3.0 terms with no additional restrictions. Abbreviations: tissue microarray (TMA), The Cancer Genome Atlas (TCGA).

To carry out this study, we started with high-throughput quantitative computational analysis of digital tissue microarray images (TMA images; small random samples of representative tumor epithelia and stroma, suitable for our image analysis software) from a cohort of lung SCC patients with survival data. The computationally measured epithelial and/or stromal features most significantly associated with survival were then reviewed manually by human researchers. On the basis of this review, we developed a human-based histologic stromal inflammation (SI) score. The intra- and inter-observer reproducibility and prognostic value of the SI score were assessed on SCC TMA cohorts by two blinded human observers. To better understand the biological basis of the histology, one human observer then scored SI on digital H&E images obtained from The Cancer Genome Atlas (TCGA) Network, and correlated histologic SI with molecular features. This research is summarized in Fig. 1.

## Results

### Datasets used for this study

This retrospective study was completed using datasets from two sources (Supplementary Data Table 1). The full tissue microarray (TMA) dataset (composed of patient cohorts 1 and 2, see Materials and Methods) from the University Hospital Zurich had relatively long-term clinical follow-up, with mean and median follow-up times of 83.4 and 83.5 months, respectively, for the censored group. The publically available TCGA clinical dataset had relatively short follow-up, with mean and median follow-up times of 18.3 and 4.7 months, respectively, for the censored group. Because of the relatively short follow-up time in the TCGA dataset, survival analyses were focused on the results from the TMA cohorts, and the TCGA cohort was used primarily for molecular correlation studies.

### Computational generation of a histologic hypothesis

**Figure 2:**
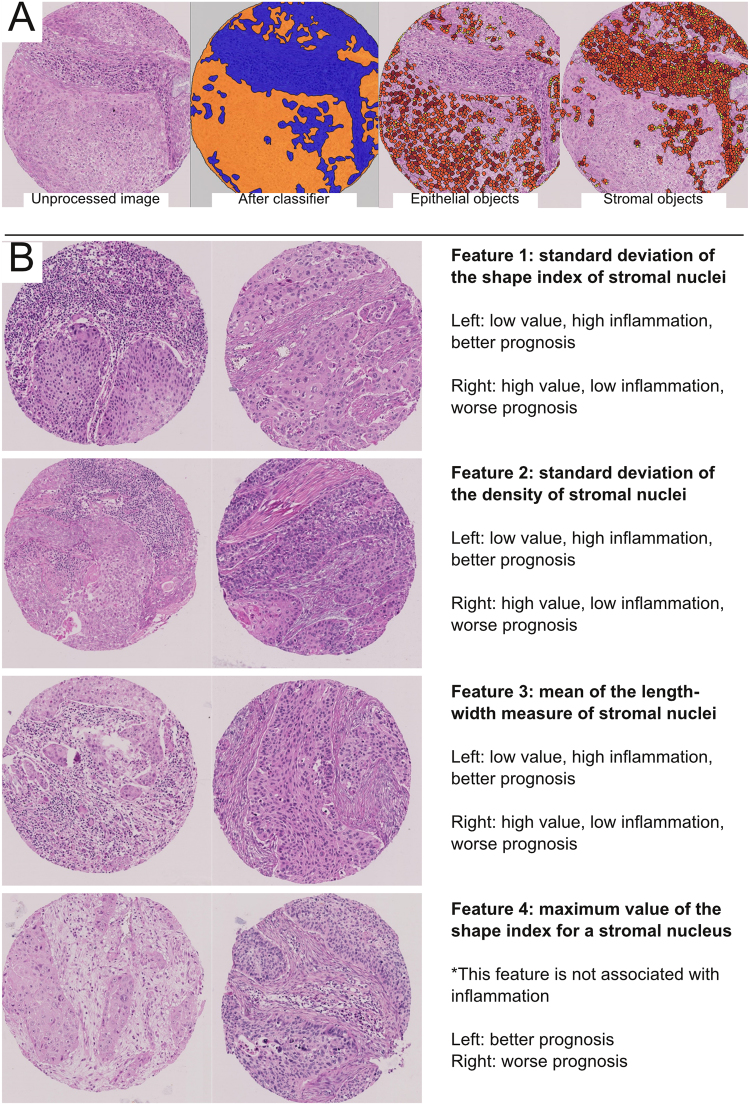
Computationally-guided histologic hypothesis generation. (**A**) Left: unprocessed H&E tissue microarray (TMA) image of lung squamous cell carcinoma. Middle-left: using labeled examples of tumor stroma and epithelia provided by one author, the software learned to divide regions of all TMA images into epithelia (orange) or stroma (blue). The computational classification was correct in many instances, but did have trouble distinguishing inflamed epithelium from inflamed stroma (typically calling all such areas stroma). Middle-right: epithelial objects (yellow = small; orange = medium; brown = large objects); while nearly every tumor nuclei is correctly accounted for, the cytoplasmic borders of some tumors were not appropriately captured, thereby underestimating the extent of at least some tumor cells (i.e. in solid tumor nests [orange areas], there should few to no gaps between epithelial objects). Right: stromal objects. 768 epithelial and 768 stromal features were quantified by the software for each image. After combining with clinical data, four computationally-measured stromal (i.e. from the blue regions) features were found to be significantly associated with overall survival at a cut-off false discovery rate of <0.05. No features from the epithelia (orange region) was significant at this cutoff. (**B**) The four significant features and representative images from the highest and lowest ranked cases (for illustration, only one of the two images for each case) is shown. Three of the four significant features were associated with the amount of stromal lymphoplasmacytic inflammation by visual review, where more inflammation was associated with better prognosis. A more complete manual review of the four features is available in the Supplementary Data (for Features 1–4).

The computational analysis was completed using the subset of digital pathology images from TMA cohort 1 only (see Materials and Methods). The initial step of the image processing was classification of TMA tissue into epithelial versus stromal regions (Fig. 2A). To train the classifier, one author provided the software with manually labeled samples of epithelia and stroma. On retrospective review, the trained classifier correctly separated epithelial from stromal regions in the majority of cases, but did have some difficulty distinguishing inflamed epithelia from inflamed stroma (Fig. 2A). Upon manual review across 8 TMA images, an estimated 77% of epithelial and stromal regions (super-pixels) were correctly classified by the computer (range from 58 to 97%, depending on the image), slightly lower than the C-Path study^4^. Subsequent to epithelial/stromal classification, nuclear segmentation was performed to identify cells within each region. Manual review revealed that the software accurately identified the vast majority of epithelial and stromal nuclei (estimated to be 97%; range from 90 to 100%, depending on the image). However, the borders of some tumor cells were not appropriately delineated (Fig. 2A).

After automated quantitative measurements of epithelial and stromal cells involving more than 1500 features, the image analysis data was combined with overall survival data (overall survival is defined as the time between the date of surgery and date last follow-up or death) from TMA cohort 1 to identify prognostic features with adjustments for multiple testing; since the each patient had two TMA samples, the numerical averages of the computationally measured values from two samples were used to correlate with survival. Interestingly, comparisons of the values from the first and second TMA images showed only weak to moderate correlations for some features (e.g. for the four features significantly associated with survival [see below], the Spearman rho values ranged from 0.32 to 0.64), consistent with significant intratumoral heterogeneity. Despite this, the resulting statistical analysis identified (among of a list of 1,500 candidate epithelial and stromal features) four computational measures of cancer stroma histology significantly associated with survival (Fig. 2B), at the pre-defined false discovery rate (FDR) < 0.05 (i.e. we would expect fewer than 5% false positives from our list of “hits”). Notably, no computational measure of the cancer epithelia was significantly associated with survival at this cutoff.

Following this analysis, we reviewed each of the four significant computational features and asked 1) if there is an obvious difference between the 10 highest and 10 lowest ranked images, and 2) if there is a way for human beings to easily evaluate this histology. An illustration of this translational process is shown in Fig. 2, and the manual review is more completely described in the Supplementary Data for Features 1–4. Notably, three of the four significant computational features demonstrated clear differences in the amount of stromal chronic inflammation between the highest and lowest ranked cases (e.g. Fig. 2B), where more inflammation was associated with better survival.

Guided by this, we designed a stromal inflammation (SI) score as the proportion of cancer desmoplastic stroma occupied by lymphocytes and plasma cells. Similar to the computational analysis, the SI score for each case was defined as the average value from the first and second TMAs, as determined by human observers. The score was intended to be relatively straightforward, and similar to the estimation of bone marrow cellularity on core biopsies, which has high inter-observer reproducibility^5^ and is a familiar task to many pathologists.

### The SI score: assessment of reproducibility and prognostic value

Two blinded human observers determined the SI scores on the full TMA dataset (composed of TMA cohorts 1 and 2). The correlation on TMAs between the two observers was found to be high (Supplementary Figure 1a, Spearman rho = 0.88), and not surprisingly, survival models based on the scores from observers 1 and 2 were very similar (see below). To avoid unnecessary duplication, the results from observer 1 are presented in the main text (Fig. 2C, Tables 2 and 3); the results from observer 2 are in the Supplementary Data unless otherwise indicated. To evaluate intra-observer reproducibility, one blinded observer re-scored a random subset of the TMA cases on a different day, and correlated the new scores with the original ones from that observer. This intra-observer concordance on TMAs was also found to be high (Supplementary Figure 1b, Spearman rho = 0.91 for observer 2).

**Table 2:** The stromal inflammation (SI) score is significantly associated with overall survival in the univariate Cox proportional hazards model for the full TMA dataset (n = 437). This list of variables is ordered by p-values (most significant listed first). The SI score is highlighted. The values for observers 1 and 2 are separated by “/.”

Variable	Type of variable	Hazard ratio (95% CI)	P-value
Overall stage	Clinical	1.64 (1.42–1.90)	1.5 × 10^−11^
Vascular invasion	Histologic	2.27 (1.79–2.89)	1.7 × 10^−11^
T	Clinical	1.53 (1.33–1.77)	3.2 × 10^−9^
Age (years)	Clinical	1.03 (1.02–1.05)	5.5 × 10^−7^
M	Clinical	3.46 (2.10–5.71)	1.1 × 10^−6^
N	Clinical	1.40 (1.21–1.63)	7.5 × 10^−6^
**SI score (observers 1/2)**	**Histologic** (**this study**)	**0**.**30** (**0**.**18–0**.**51**)**/0**.**31** (**0**.**18–0**.**55**)	**7**.**4 × 10**^**−6**^**/5**.**7 × 10**^**−5**^
Tumor size (cm)	Clinical	1.10 (1.05–1.16)	1.1 × 10^−4^
Pleural invasion	Histologic	1.48 (1.17–1.90)	0.0013
Tumor grade	Histologic	1.14 (0.91–1.42)	0.23
Gender (M/F)	Clinical	0.84 (0.64–1.12)	0.25
Pack years	Clinical	1.00 (0.99–1.00)	0.71

**Table 3:** The stromal inflammation (SI) score is significantly associated with overall survival in multivariate Cox proportional hazard (CPH) analyses for the full TMA dataset (n = 423 complete cases). The variables in the table are ordered by p-values (most significant listed first). Only the variables listed are part of the CPH analyses. The SI score is highlighted. The results for observers 1 (this table) and 2 (see Supplementary Table 3) were similar.

Variable	Hazard ratio (95% CI)	P-value
Overall stage	1.59 (1.36–1.85)	2.7 × 10^−9^
Age (years)	1.04 (1.02–1.05)	6.3 × 10^−8^
Vascular invasion	1.84 (1.44–2.37)	1.3 × 10^−6^
**SI score**	**0.32 (0.19–0.54)**	**2.5 × 10** ^**−5**^
Pleural invasion	1.16 (0.90–1.50)	0.26
Tumor grade	1.11 (0.88–1.39)	0.39

Similar to the findings for the most significant computational features, the values of SI from the first and second TMA images were found to be only moderately correlated (Spearman rho = 0.52), consistent with the presence of significant intratumoral heterogeneity in SI.

**Figure 3:**
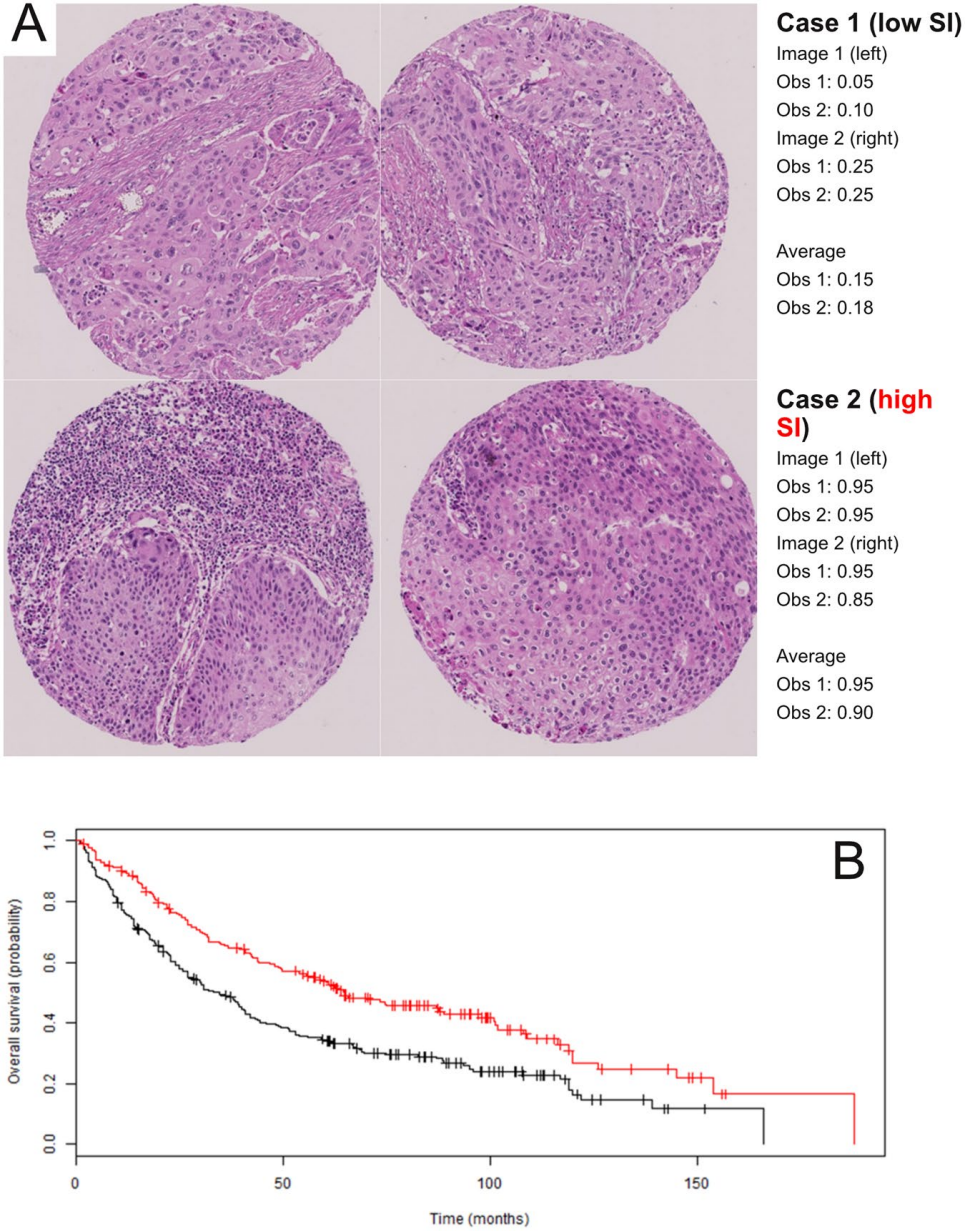
Human validation of the histologic hypothesis as a prognostic biomarker. (**A**) Example of manual scoring of SI from observers (Obs) 1 and 2. Patient 1 was deceased and had a low SI score; patient 2 was living and had a high SI score. (**B**) Kaplan Meier survival analysis for the full TMA dataset. Cases were divided into high SI (red; >median SI score; n = 195) and low SI (black; ≤median SI score; n = 229) groups. Survival was significantly better for the high SI group in comparison to the low group (median survival of 65.0 vs 33.3 months, respectively; log rank p-value = 4.6 × 10^−5^). The results for observers 1 (this Figure) and 2 (see Supplementary Figure 2) were similar.

Despite this, in survival analyses, the SI score was strongly associated with patient outcome in the full TMA cohort based on a univariate Cox proportional hazards (CPH) model (HR = 0.30; 95% CI = 0.18–0.51; p-value = 7.4 × 10^−6^; see Table 2) and Kaplan Meier survival analysis (Fig. 3), and was separately prognostic in both TMA cohorts 1 and 2 (Supplementary Data Table 3). The latter is important because the SI score was developed using cases from cohort 1 only, and the significant association based on data from cohort 2 confirmed the prognostic value of the biomarker in a separate group of patients. In the univariate analysis, overall stage, T-, N-, and M-stage, tumor size, age, vascular invasion, and pleural invasion were also prognostic (Table 2). By contrast, epithelial tumor grade (which includes the assessment of keratinization) was not significantly associated with survival (Table 2).

To address the potential impact of intratumoral heterogeneity on the prognostic value of SI, we defined an additional measure, the delta-value, as the absolute difference between the SI from the first and second TMAs in each case. We then performed correlation and univariate CPH analyses on the delta-high (delta-value > median) and delta-low (delta-value ≤ median) subgroups. These studies show that while delta-high cases demonstrated much greater heterogeneity in SI than delta-low cases (Spearman rho of −0.004 versus 0.91 between the first and second TMA images, respectively), the prognostic values of the SI scores were similar between the two groups (HR = 0.44; 95% CI = 0.17–1.17 versus HR = 0.43; 95% CI = 0.21–0.89 for delta-high and low, respectively).

The SI score was also significantly associated with overall survival in the multivariate CPH model (HR = 0.32; 95% CI: 0.19–0.54; p-value = 2.5 × 10^−5^; see Table 3), confirming that it contributes important prognostic information independently of other clinical and pathologic biomarkers currently in use. Please note that only a subset of the biomarkers from the univariate analysis was included in this analysis to avoid redundancy (e.g. we included overall stage, but not T-, N-, or M-stage). In this multivariate model, the strongest independent predictors of survival were (in order of statistical significance) overall stage, patient age, vascular invasion, and SI score. Pleural invasion and tumor grade were not significant in this multivariate model, after controlling for other variables.

### The relationship between SI and other biomarkers in the tumor microenvironment

To better understand the biological underpinnings of the histology, we studied the molecular correlates of the SI score. SI scores (see Materials and Methods) were determined on TCGA digital H&E full slide images by one human observer (observer 2). On molecular correlation studies using TCGA RNA-seq data from the corresponding cases, we found that SI scores were significantly and positively correlated with the expression of 265 genes at a pre-defined FDR of <0.05 (see Supplementary RNA-seq Correlation Data); not surprisingly, the list of genes were enriched for those involved in the immune system, including the activation of alpha-beta effector T-cells and regulatory T-cells. The score was not significantly associated with the number of non-silent mutations (Supplementary Figure 3), germline single nucleotide polymorphisms (SNPs), or somatic mutations in individual genes (after adjusting for multiple testing in the cases of SNPs and mutations).

**Figure 4:**
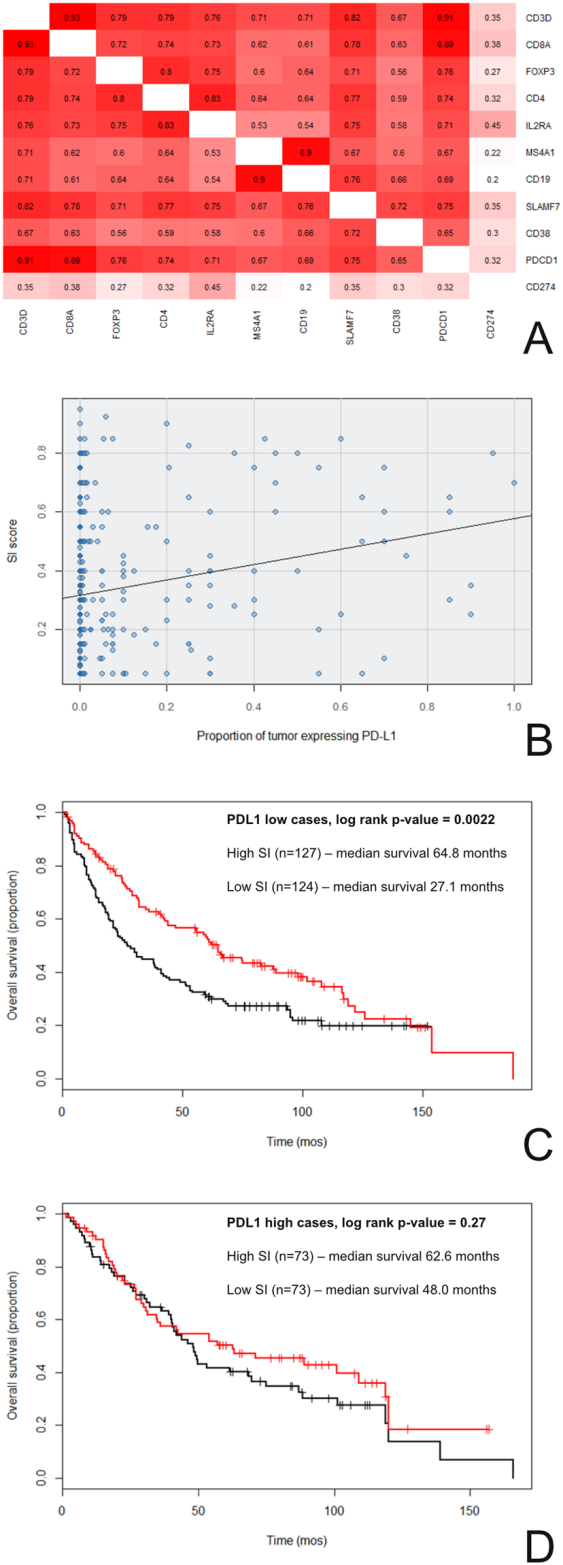
The relationship between SI, PD-L1 expression, and overall survival. (**A and B**) PD-L1 expression was not strongly associated with inflammation. (**A**) By gene expression profiling, *CD274* (PD-L1) RNA levels did not correlate strongly with RNA levels of genes expressed by immune cells in lung SCC TCGA cases. The numbers in each box are the Spearman rho values for the expression levels of the gene-pair combinations (red = high correlation; white = low correlation). (**B)** PD-L1 protein expression by immunohistochemistry did not correlate strongly with histologic SI scores in lung SCC TMA cases (Spearman rho = 0.20). (**C and D**) The prognostic value of the SI score is modified by epithelial PD-L1 expression. Cases from the TMA cohorts were separated by PD-L1 expression (high versus low). (**C**) SI and survival when PD-L1 expression was low. (**D**) SI and survival when PD-L1 expression was high. The interaction term for this Cox proportional hazards model was trending towards significance (interaction p-values = 0.056). The results from observer 1 (this Figure) and observer 2 (Supplementary Figure 4) were similar.

To further study the relationship between candidate prognostic and predictive biomarkers in the tumor microenvironment, we focused on the RNA expression levels of selected genes using the TCGA dataset. *CD3D* and *CD8A* were chosen to represent cytotoxic T cells, *MSA4* (CD20 surface antigen) and *CD19* to represent B cells, *CD38* and *SLAMF7* (CD319 surface antigen) to represent plasma cells (*SDC1*, coding for syndecan 1/CD138 surface antigen, was not used because it is also expressed by the SCC tumor epithelia), *CD3*, *CD4*, *FOXP3* and *IL2RA* (CD25 surface antigen) to represent regulatory T-cells, and *PDCD1* (PD-1) and *CD274* (PD-L1) as targets of cancer immune checkpoint blockers. We determined the Spearman correlations between the expression levels of all possible pairs from this selected gene-list, and identified interesting patterns (Fig. 4A). First, the immune cell type markers and *PDCD1* (PD-1) were moderately to very strongly correlated with one another. Second, *CD274* (PD-L1) did not follow this trend, and was only weakly to moderately correlated with the other markers. We hypothesized that this weaker correlation was due to the fact that PD-L1 (unlike the other markers) is expressed not only on immune cells, but also to varying degrees on the tumor epithelia of some but not all lung SCCs.

Because of the apparently orthogonal nature of SI and PD-L1 expression from the TCGA analysis, and the role of PD-L1 in tumor immune evasion, we investigated whether epithelial PD-L1 protein expression as determined by immunohistochemistry (IHC, see Materials and Methods) modified the prognostic value of the SI score in our TMA cohorts. Consistent with the TCGA findings, the SI score was weakly associated with epithelial PD-L1 protein expression (Spearman rho = 0.20, Fig. 4B). Accordingly, we stratified the TMA lung SCC cases into PD-L1 protein expression high (proportion of tumor cells expressing PD-L1 > 0.00; n = 147) and protein expression low (proportion of tumor cells expressing PD-L1 = 0.00; n = 260) groups, and formally tested for a statistical interaction between PD-L1 protein expression (high versus low) and the SI score in a CPH survival model. This analysis revealed that epithelial PD-L1 expression likely modified the prognostic value of the SI score (interaction p-value = 0.056). Not surprisingly, the SI score was highly prognostic when PD-L1 expression was low (Fig. 4C, log rank p = 0.0022), and lost much of this prognostic value when PD-L1 expression was high (Fig. 4D, log rank p = 0.27).

## Discussion

The routine prognostic stratification lung SCC is accomplished by considering multiple parameters at diagnosis, including the anatomical extent and spread of the cancer (e.g. stage), the microscopic features of the cancer (e.g. the tumor grade), and the patient’s demographic and other clinical characteristics (e.g. age)^6^. Tumor grade, pleural invasion, and lymphovascular invasion are standard histologic features evaluated by pathologists using H&E stained slides at the microscope. This evaluation is focused on features of the neoplastic epithelia and its relationship to the surrounding structures (e.g. pleural membranes and vessels). By contrast, features from the non-neoplastic cancer stroma are not a part of this routine microscopic examination at present time.

For this study, we employed computational pathology as a guide for the translational development of a stromal histologic biomarker for potential routine use by human pathologists. The computational screen we performed is analogous to screens in genomics research, and permitted the rapid and efficient prioritization of candidate histologic features (or “hits”) for downstream evaluation by human researchers, thereby improving the efficiency of pathology research.

Before proceeding further, it is necessary to point out there were a number of significant factors that limited the sensitivity of our screen. First, some potentially important histologic features were not assessed. These include features related to higher-order tissue architecture (e.g. the pattern of cancer infiltration^7^), relational features (e.g. the presence of tumor in blood vessels), features that cannot be assessed on small TMAs (e.g. the overall extent of the tumor), and other features not measured by the software (e.g. the number of mitotic figures). Second, some features included in the screen may not have been measured accurately, potentially due to errors involving the epithelial-stromal classifier and the delineation of cytoplasmic boundaries. The impact of these errors on the screen is difficult to define. While our software had difficulties separating inflamed epithelia from inflamed stroma (Fig. 2A), it nevertheless correctly highlighted the prognostic importance of stromal inflammation. While inaccurate cell borders likely adversely impacted the measurement of cytoplasmic features, there are no suitable cytoplasmic features that could have served as reliable positive controls (e.g. keratinization is not reliably associated with survival, and there is no reason to believe that our analysis should have identified features related to keratinization as “hits”). Overall, while our screen can be considered high-throughput and systematic, it is by no means a perfect or comprehensive analysis of cancer microscopic anatomy.

The limitations just discussed are highly dependent on the performance of the image analysis software. More recently, sophisticated algorithms have been developed to better quantify complex histologic patterns on whole slide images (i.e. the analyses are no longer limited to small TMAs), including the spatial heterogeneity of different cell types (e.g. stromal, lymphocyte, and tumor) in the cancer and its environment^8–10^. Further, recent applications of deep learning approaches to pathology have led to diagnostic algorithms with accuracies comparable to that for human pathologists^11–13^. While the computational screening we performed for this study is less sophisticated and less accurate than the best-available tools, our overall approach is distinct from recent publications and remains relevant. Despite technological advances, there are still significant hurdles for the routine application of computational tools in clinical practice (some of which are outlined in Table 1). By contrast, H&E biomarkers developed for human use (guided by computational pathology screening approaches) should find far easier paths towards clinical acceptance, at least for the more immediate future, until computational methods are more accepted and better integrated into daily clinical workflows^11^.

The human-based SI score performed well in our preliminary validation studies. The inter- and intra-observer concordances of the SI score were high (Supplementary Figure 1a and b, respectively) on TMA samples, at values that compare favorably to published literature for similar histologic biomarkers^14^. This is not surprising since we modeled our score after the assessment of bone marrow cellularity, which is a practice that is widely-accepted clinically and highly reproducible between pathologists^5^. It is important to note, however, that the TMA samples used in this study (best considered as random small samples of representative cancer epithelia and stroma), while ideally suited for our image analysis methods, are not the same as real samples that come from the operating room (i.e. lung cancer small biopsies and full slide section of tumor). In addition, the moderate correlation in SI between the first and second TMA samples indicates that there is significant intratumoral heterogeneity in SI within lung SCCs. The implication of this heterogeneity is that the SI score (based on two small TMA samples), while precise in the setting of our study, may fail to accurately capture the “true” average SI in large tumor specimens. Further, the intra- and inter-observer concordance in SI could be considerably lower for full slide sections (as different observers can choose different areas with different amounts of inflammation to score). On the other hand, we are hopeful that the concordance in SI would remain high for lung cancer biopsies (the most common type of specimens for lung cancer diagnoses) since these are relatively small samples, and thus more similar to TMAs than full slide sections in this regard. One potential remedy for improving concordance on larger full sections would be to sample a larger number of areas to reduce the errors in the means and account for heterogeneity. Alternatively, quantitative computational research methods that systematically measure histologic heterogeneity in the microenvironment can be applied to guide suitable scoring strategies^9^. Such follow-up studies would require real clinical samples (i.e. biopsies and full slide sections rather than TMAs), however, and are therefore beyond the scope of the current manuscript.

Remarkably, SI scores based on two small TMA samples proved highly prognostic (even in cases that show high heterogeneity in SI), and it is conceivable that the correlation between SI and survival may strengthen further with additional sampling^4,9^. The SI score outperformed tumor grade and pleural invasion in univariate (Table 2) and/or multivariate survival models (Table 3). The lack of significance for tumor grade is not surprising given that data from other groups showed that epithelial keratinization may be of limited prognostic relevance^3^. In contrast to tumor grade as it is currently defined, the large effect size for SI in the multivariate model (Table 3; hazard ratio of 0.32; 95% CI of 0.19 to 0.54) underscores the independent prognostic value of histologic features from the tumor microenvironment, and supports their potential inclusion in revised prognostic grading schemes for primary lung SCC.

Overall, our findings about the prognostic impact of SI are in keeping with existing literature (reviewed by Remark *et al*.^15^). Using a variety of methods, other groups have described the prognostic importance of CD3/CD8 positive T lymphocytes^16–19^, B lymphocytes^19–21^, dendritic cells^22–24^, and macrophages^25,26^ in patients with NSCLCs. The majority of the published literature, however, did not specifically address inflammation in lung SCCs. In addition, some of the technologies used to evaluate the tumor microenvironment (e.g. C-Path) are not yet widely available or validated, and may involve complex algorithms that are often not easily amenable to human interpretation and verification. By comparison, our relatively straightforward score was developed for routine H&E histology, requires no immunohistochemical stains, and could be easily and robustly adapted by researchers and pathologists in anatomical pathology and many research laboratories, with no additional investment of monetary and technical resources.

The ubiquitous nature of H&E histology also allowed us to easily extend our efforts to other datasets. On correlation studies with other biomarkers in the stroma, we identified relatively weak associations between PD-L1 expression and measures of inflammation, in both the TCGA and our TMA datasets (Fig. 4A and B). Consistent with this and the role of PD-L1 in tumor immune evasion, our interaction survival analyses demonstrated that epithelial PD-L1 expression likely modified the prognostic significance of SI in the lung SCC TMA cohorts. As expected, SI was only strongly prognostic when epithelial PD-L1 expression was low (Fig. 4C and D).

Beyond prognostic considerations, the largely independent relationship between SI and PD-L1 has implications for research into predictive biomarkers for immune checkpoint blockers. Current research mostly focused on single biomarker classes in isolation: e.g. those related to tumor immune evasion (e.g. PD-L1)^27–31^, inflammation in the tumor microenvironment (e.g. expression profiling of immune cells)^31–33^, or tumor mutational/neo-antigen burden^34^, although multi-parameter approaches have also been proposed^35^. Our data suggested that PD-L1 and SI could be integrated, and raised the possibility that a dual-biomarker integrating both SI and PD-L1 could outperform biomarkers based on either inflammation or PD-L1 alone. Unfortunately, we could not evaluate the SI score as a predictive biomarker for checkpoint blockers in this study, because our patients did not receive this type of treatment.

The present study does have a number of other limitations. First, our characterizations of the molecular correlates of cancer stromal inflammation were likely negatively impacted by extreme multiple testing adjustments in some analyses (e.g. for 20 thousand genes and 650 thousand SNPs evaluated), and by the use of different tissue fragments for histologic and molecular analyses (see Materials and Methods). Second, the PD-L1 antibody we used (E1L3N) is not currently approved by the Food and Drug Administration and some of our archival blocks were older; these considerations likely limit the clinical applicability of our SI/PD-L1 interaction analysis. Third, our survival analyses were largely based on the retrospective evaluation of a large lung SCC dataset with good follow-up from a single institution, and replication of the results in other prospective cohorts would be ideal. Finally, our patients were not part of a randomized study and treatment data were not systematically analyzed; as such, differences in treatments and other unaccounted-for variables could limit our univariate and multivariate models.

In conclusion, we performed a systematic and high-throughput computational screen for prognostic histologic features in lung SCCs. Based on the results of the screen, we developed and successfully validated a reproducible and strongly prognostic stromal inflammation H&E biomarker for TMAs, one that requires no immunohistochemistry and no special software. While additional validation on real clinical specimen is required, our translational efforts confirm that computational image analysis is an efficient hypothesis generator for human pathologists, and implies that such approaches can be used for biomarker discovery in other tumor types and in other clinical settings (e.g. predictive biomarker research). Finally, the large impact of SI on survival in our cohorts supports its further investigation, potentially as a component of a revised prognostic grading score for lung SCCs, in combination with measures of tumor budding/fragmentation and keratinization^3,7,36^.

## Materials and Methods

### Ethics statement

The study was approved by the Ethical Commission of the Canton of Zurich under reference number KEK ZH-Nr. 29-2009/14. All methods were performed according to relevant guidelines and regulations.

### Experimental design summary

This retrospective study was completed using two TMA lung SCC datasets from the University Hospital Zurich (Zurich, Switzerland) and full-slide lung SCC digital images publically available through TCGA (Supplementary Data Table 1). TMA cohort 1 consisted of 232 consecutive de-identified patients with surgically resected primary lung SCC from 1993–2002. TMA cohort 2 consisted of 205 consecutive de-identified patients with surgically resected primary lung SCC from 2003–2008. The only survival measure reported in this study is overall survival, defined as the time from the date of surgery to the date of last follow-up or death. Aside from the SI score and PD-L1 protein expression, all other biomarkers (e.g. tumor grade, age, and stage) were determined at the time of diagnosis for the TMA cases. The study was completed in two parts (Fig. 1). In part 1A, the computational screen and histologic score development (detailed below) was conducted using TMA cohort 1 cases only. In part 1B, the manual evaluation of the SI score (defined below) was performed by two blinded observers using digital images from both TMA cohorts 1 and 2, where cohort 2 served as an independent validation dataset for the prognostic value of the histologic SI score developed using TMA cohort 1. For part 2, the histologic-molecular correlations study was completed using publically available TCGA data. Digital full slide images from 350 TCGA cases were accessed online through the Digital Cancer Slide Archive^37^. TCGA molecular datasets were downloaded from the Broad GDAC database (https://gdac.broadinstitute.org/). Of note, TCGA images from formalin-fixed paraffin embedded (FFPE) sections were preferentially selected for scoring due to better tissue histology; by contrast, the molecular analyses by TCGA were performed primarily on frozen unfixed tissue. As such, the histologic scoring and molecular analyses were often performed on different samples obtained from the same tumor.

### Case inclusion and exclusion criteria and TMA construction

Cases with adenocarcinoma, large-cell, adeno-squamous, neuroendocrine, and sarcomatoid features on histology, cases of cancer metastases, relapsed tumors, and patients with less than one month survival post-surgery were excluded from this study. Treatments administered were neither evaluated for this study, nor used for including/excluding cases. For each TMA case, a pathologist selected two representative areas of tumor by evaluating full slides; within each selected area of the corresponding paraffin embedded blocks, one randomly selected 0.6 mm diameter tissue core was taken. As such, there are two digital pathology TMA images from each case, and these are best regarded as two random samples of representative tumor epithelia and stroma. Digital images from the Digital Cancer Slide Archive were filtered to include only the higher quality “DX” or “diagnostic” (mostly FFPE tissue) images.

### Computational scoring of tissue microarray images, histologic hypothesis generation, and retrospective technical assessments of image analysis

We performed computational analysis of H&E stained lung SCC TMA digital images using the Definiens Tissue Studio image analysis software (Version 3.6.1). One author (DX) trained the software to segment the images into epithelial and stromal regions and to perform nuclear identification and segmentation (this is the only part of the computational image analysis that involved human intervention). The software then automatically extracted summary measures (mean, standard deviation, maximum, and minimum) of size, shape, and texture from nuclear and cytoplasmic objects from both the tumor epithelia and stroma. In total, 1,536 features (half stromal, half epithelial) were analyzed in each image from TMA cohort 1 (464 TMA images from 232 patients). Since each case has two TMA images, we used the average value from the first and second TMA images to correlate with survival. We used significance analysis of microarrays analysis^38^ to identify computational histologic features significantly associated with overall survival (based on a Cox proportional hazards model) at a pre-defined false discovery rate (FDR) < 0.05.

One observer retrospectively assessed the accuracy of the epithelial-stromal classifier by comparing computationally labeled epithelial and stromal regions (i.e. super-pixels) against human classifications across 8 TMA images. The accuracy of the nuclear identification was assessed by comparing computationally labeled nuclei against human labels across the same 8 TMA images. To avoid bias, the observer assessed all super-pixels and nuclei in contact with the horizontal diameter of each TMA image.

### Scoring of cancer stromal inflammation and PD-L1 expression

We visually reviewed the extreme cases (with the 10 highest and lowest values) for each of the computational features significantly associated with survival, and found that three of the four features were associated with stromal chronic inflammation (SI). The SI score was defined as the proportion of cancer desmoplastic stromal area (the denominator for score) occupied by chronic inflammatory cells (the numerator) on H&E stained digital images. Since there were two TMA images for every case, the average of the SI values from the first and second TMA images for each case was used to correlate with survival in most analyses. For each image, score values ranged from 0.00 to 1.00, with increments of 0.10; cases with values in between increments were recorded as a middle value (e.g. a score estimated to be between 0.30 and 0.40 is recorded as 0.35). Only lymphocytes and plasma cells were counted towards the score, since based on our manual review, other stromal elements (neutrophils, macrophages, and endothelial cells) were not obviously different between the highest and lowest ranked cases; only inflammatory cells in the peri-tumoral stroma were scored because the significant computational features were identified as “stromal” by the screen (i.e. intraepithelial lymphocytes were not scored); because the significant computational features associated with inflammation were standard deviation and mean measures, we defined the stromal inflammation as a proportion, a statistical measure of average.

The human-based SI score was determined on digital images from both TMA cohorts 1 and 2 from the University Hospital Zurich (437 patients, 874 TMA images). Manual scoring was performed by one observer from University Hospital Zurich and one observer from the Beth Israel Deaconess Medical Center, blinded to all clinical data. For intra-observer correlation studies, one observer (DX) scored a random subset of 50 cases from the full TMA cohorts on a different day, and correlated the results with the original scores. The score for each patient/case was the mean of the scores for the two TMA images for each observer. If neither image for a patient was scored (e.g. not enough tumor cells), the case was NA (not available). For the TCGA cohort, one observer (DX) determined the SI score based on two randomly selected areas of tumor stroma at high magnification (to simulate the SI score from the TMAs) from 350 cases, blinded to all clinical and molecular data. If a case had more than one image, only the first image was scored. Cases that could not be scored were classified as NA.

Epithelial PD-L1 expression was scored on the SCC TMAs from cohorts 1 and 2 by immunohistochemistry performed using the E1L3N anti-PD-L1 antibody (Rabbit monoclonal antibody #13684, Cell Signaling Technology, Danvers, MA). One observer (DX) scored epithelial PD-L1 expression on TMA digital images as the proportion of viable tumor cells with circumferential PD-L1 staining of any intensity (range from 0.00 to 1.00). Stromal PD-L1 expression was not scored for this study. Each TMA must have at least 100 viable tumor cells. The score for each patient/case was the mean of the scores for the two TMA images for the single observer. If neither image for a patient was scored (e.g. not enough tumor cells), the case was NA.

### Statistical analyses

The Spearman rho was used to assess the correlation 1) between the SI scores from the two observers, 2) between the SI scores from the same observer on different days, 3) between SI and transcript levels of genes characteristic of specific immune cell types, and 4) between the SI score and the number of non-silent mutations. Kaplan Meier and univariate/multivariate Cox proportional hazards analyses were performed to assess the prognostic significance of the SI score in multiple settings; complete details of survival analyses are described in the figure and table legends, and the main text. Significance of microarray analyses^38,39^ were performed to identify 1) computational features significantly associated with survival (using a Cox proportional hazards model), and 2) gene transcript levels significantly associated with the SI score; both analyses used a pre-defined false discovery rate of < 0.05 as a significance cut-off. All statistical analyses were performed using R (version 3.2.2^40^).

### Data availability

The datasets generated and/or analyzed during the current study are available from the corresponding authors on reasonable request.

## Electronic supplementary material

Supplementary data

List of genes most strongly correlated with SI score
