# Supplementary material for: Computationally-Guided Development of a Stromal Inflammation Histologic Biomarker in Lung Squamous Cell Carcinoma
Source: Sci Rep. 2018 Mar 2;8:3941. doi: 10.1038/s41598-018-22254-4 (PMC5834457; doi:10.1038/s41598-018-22254-4)
Supplement: Supplementary file 1 — Supplementary data [file 41598_2018_22254_MOESM1_ESM.pdf]

Title: Computationally-Guided Development of a Stromal Inflammation Histologic Biomarker in Lung Squamous Cell Carcinoma

Authors: Daniel Xia MD, Ruben Casanova MSc, Devayani Machiraju MSc, Trevor D. McKee PhD, Walter Weder MD/Prof, Andrew H. Beck MD/PhD, Alex Soltermann MD/Prof

Supplementary Data

**Title**	**Page**
Supplementary Figure 1. SI scores show high intra- and inter-observer correlation	2
Supplementary Figure 2. Kaplan Meier survival analysis for SI score, observer 2	3
Supplementary Figure 3. The SI score was not significantly associated with the number of non-silent mutations in the TCGA dataset	4
Supplementary Figure 4. The relationship between SI, PD-L1 expression, and survival (based on observer 2's SI scores)	5
Supplementary Table 1. Comparison of tissue microarray (TMA) and The Cancer Genome Atlas (TCGA) datasets.	7
Supplementary Table 2. The SI score was consistently associated with overall survival in the univariate Cox proportional hazard model in multiple analyses for both observers	8
Supplementary Table 3. The stromal inflammation (SI) score for observer 2 was significantly associated with overall survival in multivariate Cox proportional hazard (CPH) analyses, for the full TMA dataset	9
Pathologist Review for Feature 1	10
Pathologist Review for Feature 2	36
Pathologist Review for Feature 3	61
Pathologist Review for Feature 4	86

Supplementary Figure 1. SI scores show high intra- and inter-observer correlation. **a.** Inter-observer concordance is high (Spearman rho = 0.88, n = 429). The equation for line of best fit (observer 1 = observer 2 * 0.99 – 0.00) suggests that the scores between the two observers are very close numerically. **b.** Intra-observer concordance is high (Spearman rho = 0.91, n = 47). 50 random cases were selected for intra-observer correlation. The equation for the line of best fit is: day 2 = day 1 * 0.97 - 0.05, suggesting that the scores from the two different days are very close numerically.

a.

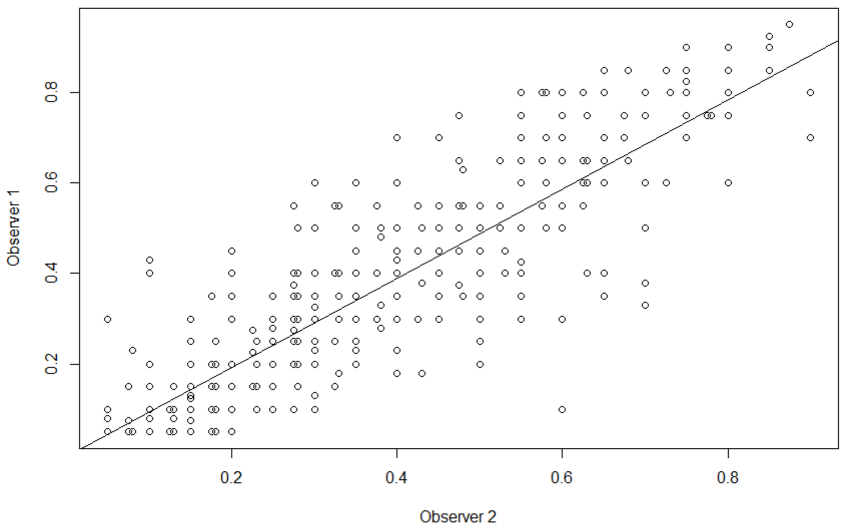

b.

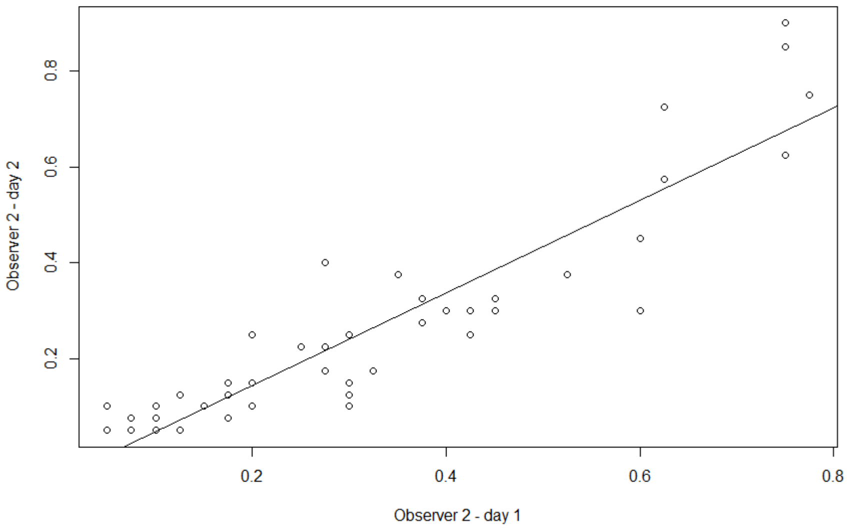

Supplementary Figure 2. Kaplan Meier survival analysis for SI score, observer 2. SI scores were divided into high (red; > median SI score; n= 210) and low (black; < median SI score; n= 213) groups. Survival was significantly better for the high than the low group (median survival of 63.3 vs 34.9 months, respectively; log rank p-value = 0.0034). The results were similar to observer 1 (main text Figure 2c).

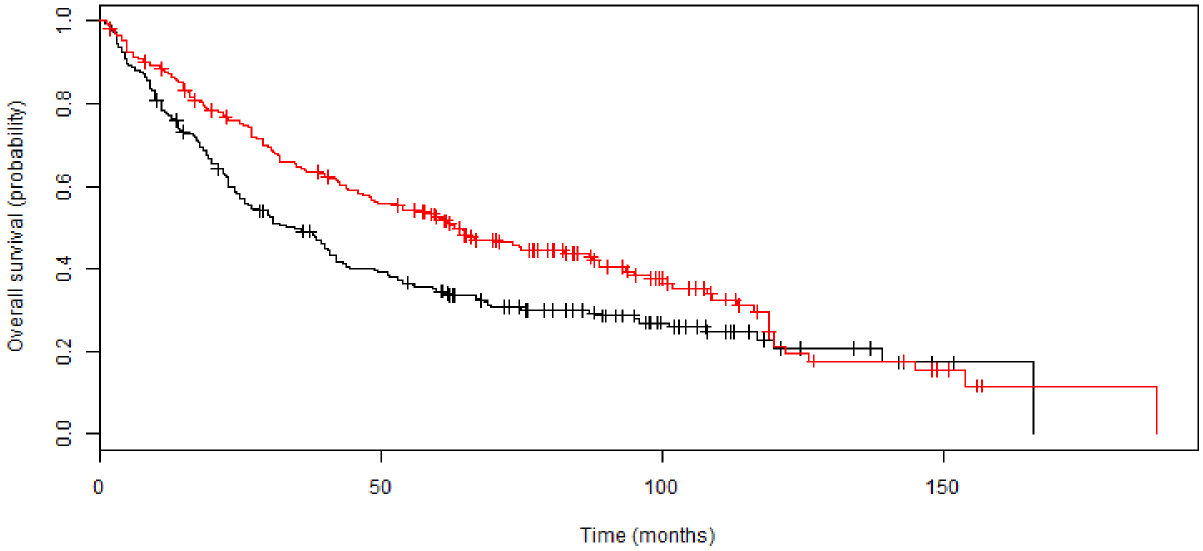

Supplementary Figure 3. The SI score was not significantly associated with the number of non-silent mutations in the TCGA dataset (complete cases = 120, Spearman rho = 0.032, p = 0.75).

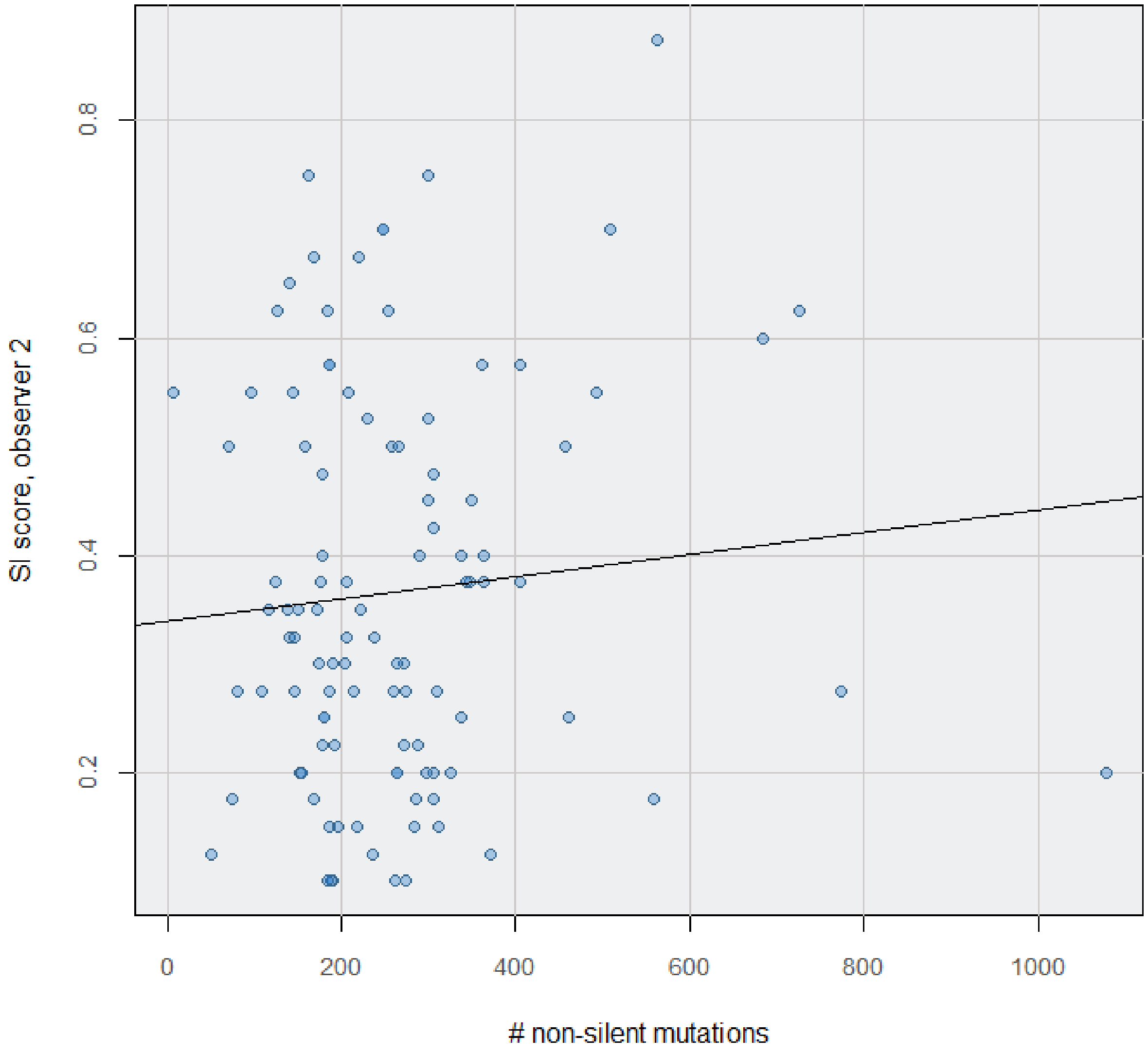

Supplementary Figure 4. The relationship between SI, PD-L1 expression, and survival (based on scores from observer 2). a. PD-L1 expression on immunohistochemistry was not strongly associated with the SI score (Spearman rho = 0.23). **b-c.** The prognostic value of SI depended on PD-L1 expression. Cases from the TMA cohorts were separated by PD-L1 expression (high versus low). High and low SI scores were defined as for Figure 1 of the main text. **b.** SI and survival in when PD-L1 expression was low. **c.** SI and survival when PD-L1 expression was high. The interaction term was borderline significant (interaction p-value = 0.048, respectively). The results from observers 1 (main text Figure 3) and 2 (this Supplementary Figure) were similar.

a.

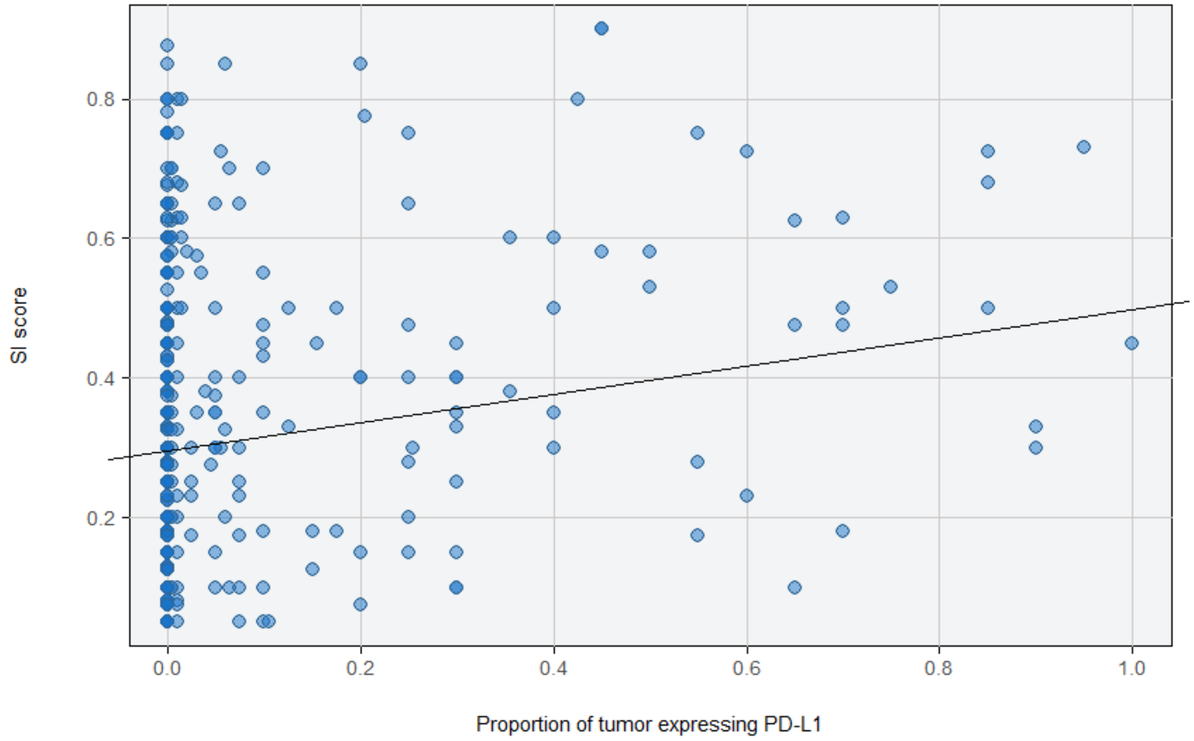

b.

PDL1 low cases, log rank p-value = 0.007
High SI (red, n=123) – median survival 65 months
Low SI (black, n=130) – median survival 27 months

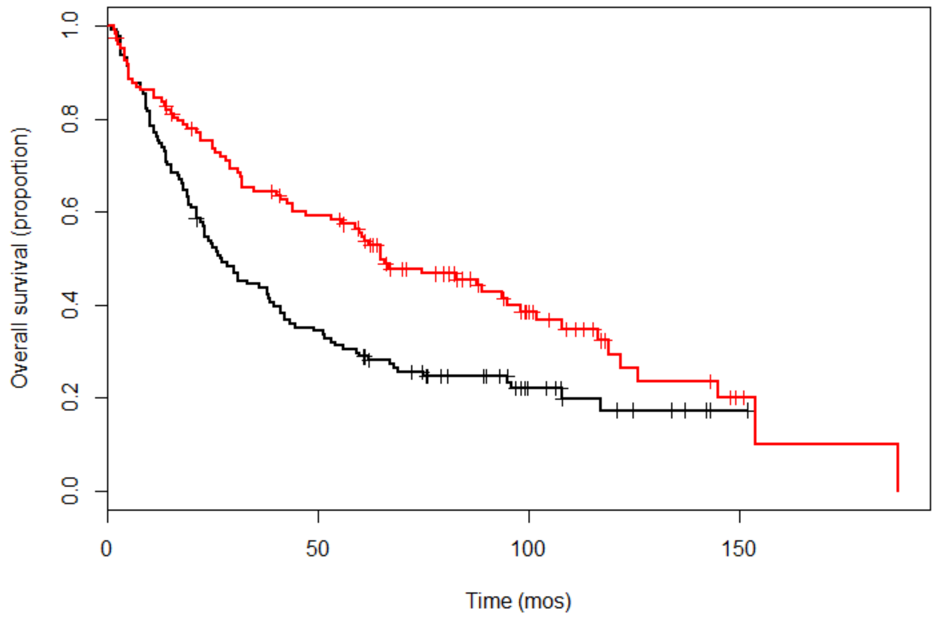

c.
PDL1 high cases, log rank p-value = 0.77
High SI (red, n=73) – median survival 54 months
Low SI (black, n=72) – median survival 44 months

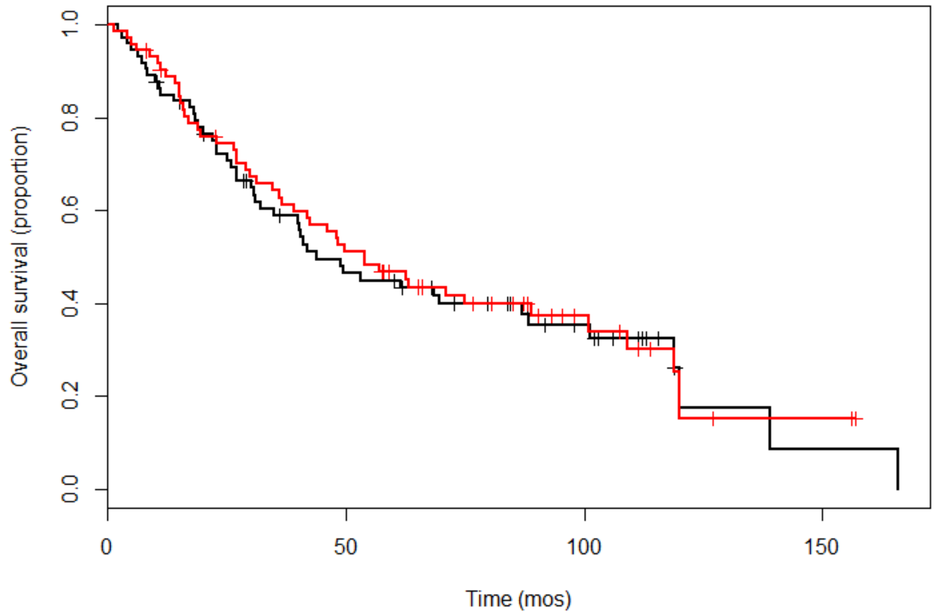

Supplementary Table 1. Comparison of tissue microarray (TMA) and The Cancer Genome Atlas (TCGA) datasets. The mean and median follow-up times for the TMA datasets were longer than that for the TCGA dataset.

	TMA training (cohort 1)	TMA validation (cohort 2)	TMA full (training + validation)	TCGA
**Type(s) of study performed**	Computational screen, manual validation of SI score, correlation between manual scores and survival data	Manual validation of SI score, correlation between manual scores and survival	See TMA training and validation datasets	Manual scoring only, correlation between manual scores and molecular data
**No. of cases total**	232	205	437	350
**No. of cases scored as "NA" by observer 1 (%)**	5 (2.1%)	7 (3.4%)	12 (2.7%)	30 (8.6%)
**No. of deaths (%)**	166 (72%)	130 (63%)	296 (68%)	85 (24%)
**Gender (M / F)**	184 / 48	154 / 51	338 / 99	262 / 88
**Mean / median follow-up for censored observations (mos)**	86.4 / 85.0	80.7 / 82.7	83.4 / 83.5	18.3 / 4.7
**Mean / median age (years)**	65.5 / 66.0	65.0 / 65.0	65.3 / 66.0	66.9 /68
**Stage 1 (%)**	77 (32%)	71 (35%)	148 (34%)	170 (49%)
**Stage 2 (%)**	83 (36%)	74 (36%)	157 (36%)	113 (33%)
**Stage 3 (%)**	60 (26%)	58 (28%)	118 (27%)	62 (18%)
**Stage 4 (%)**	12 (5.2%)	2 (1.0%)	14 (3.2%)	3 (0.86%)

Supplementary Table 2. The SI score was consistently associated with overall survival in the univariate Cox proportional hazard model in multiple analyses for both observers. Although the SI score was not significantly associated survival in the TCGA dataset, the magnitude and direction of the hazard ratio was compatible with findings in the TMA datasets. The follow-up time for the TCGA dataset was considerably shorter (see Supplementary Data Table 1).

SI score for	Dataset (n)	Hazard ratio observer 1 (95% CI)	P-value observer 1	Hazard ratio observer 2 (95% CI)	P-value observer 2
**Patient**	TMA training (232)	0.31 (0.15-0.61)	7.7×10^{-4}	0.25 (0.12-0.54)	4.4×10^{-4}
**Patient**	TMA validation (205)	0.30 (0.13-0.67)	0.0036	0.40 (0.17-0.94)	0.036
**Image 1 of 2 only**	Full TMA (437)	0.41 (0.25-0.67)	3.5×10^{-4}	0.41 (0.24-0.70)	0.0011
**Image 2 of 2 only**	Full TMA (437)	0.43 (0.27-0.69)	4.1×10^{-4}	0.40 (0.24-0.67)	5.2×10^{-4}
**Patient**	TCGA (350)	NA	NA	0.49 (0.13-1.70)	0.26

Supplementary Table 3. The SI score (from observer 2) was significantly associated with overall survival in multivariate Cox proportional hazard (CPH) analyses, for the full TMA dataset (n = 422 complete cases). The variables in the table were ordered by p-values (most significant listed first). Only the variables listed were part of the CPH analyses. The results between observer 1 (main text Table 3) and 2 (this Supplementary Table) were similar.

Variable	Hazard ratio (95% CI)	P-value
**Overall stage**	1.59 (1.37-1.86)	3.2×10^{-9}
**Age (years)**	1.04 (1.02-1.05)	1.4×10^{-7}
**Vascular invasion**	1.92 (1.50-2.46)	2.5×10^{-7}
**SI score**	**0.34 (0.19-0.61)**	$\mathbf{2.6 \times 10^{-4}}$
**Tumor grade**	1.12 (0.89-1.41)	0.32
**Pleural invasion**	1.11 (0.86-1.44)	0.41

Stroma.Nucleus.Std..Dev..Shape.index

What is Stroma.Nucleus.Std..Dev..Shape.index?

The Shape index describes the smoothness of an image object border. The smoother the border of an image object is, the lower its shape index. It is calculated from the Border Length feature of the image object divided by four times the square root of its area.

Parameters

- b_v is the image object border length
- $4\sqrt{\#P_v}$ is the border of square with area $\#P_v$

Expression

$$\frac{b_v}{4\sqrt{\#P_v}}$$

Feature Value Range

$[1, \infty]$; 1 = ideal.

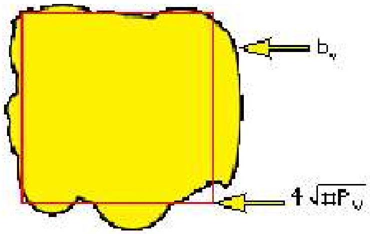

Figure 24.17. Shape index of an image object v

High 1

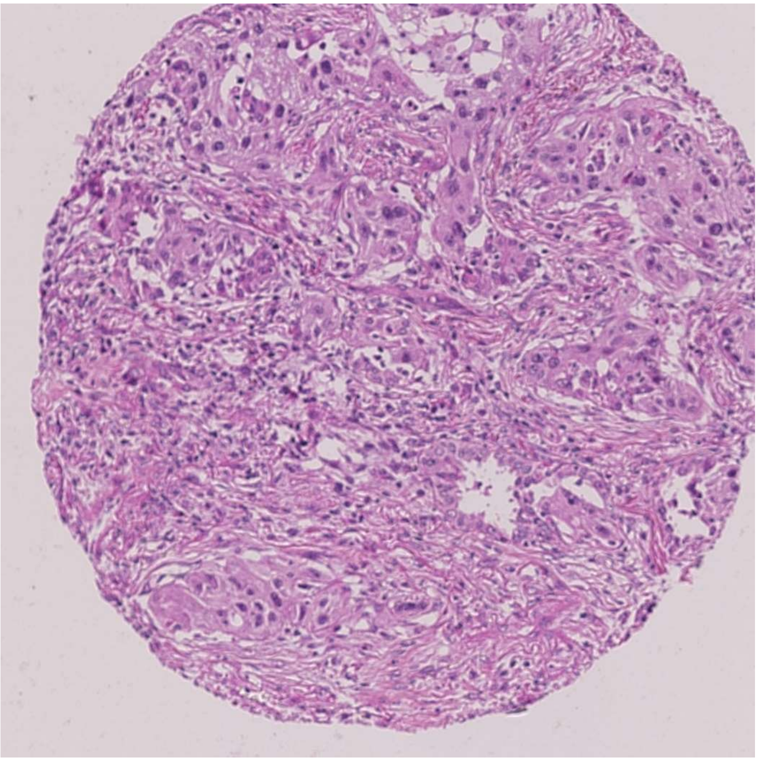

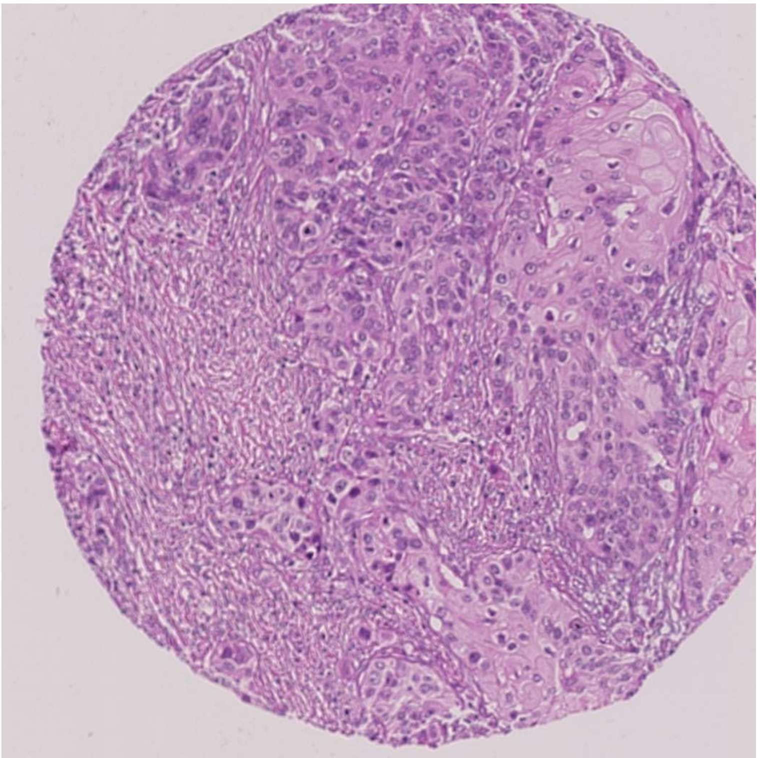

High 2

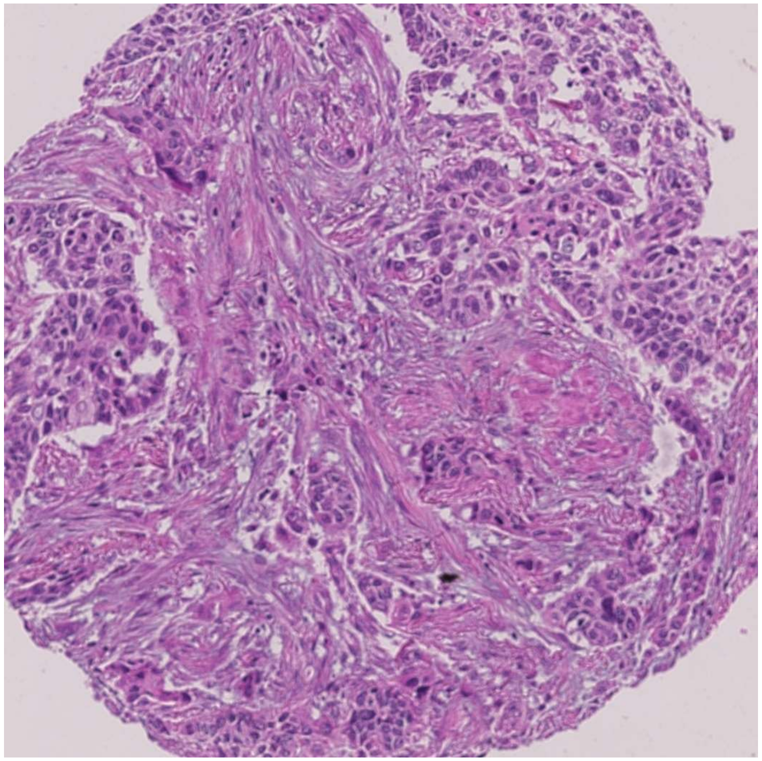

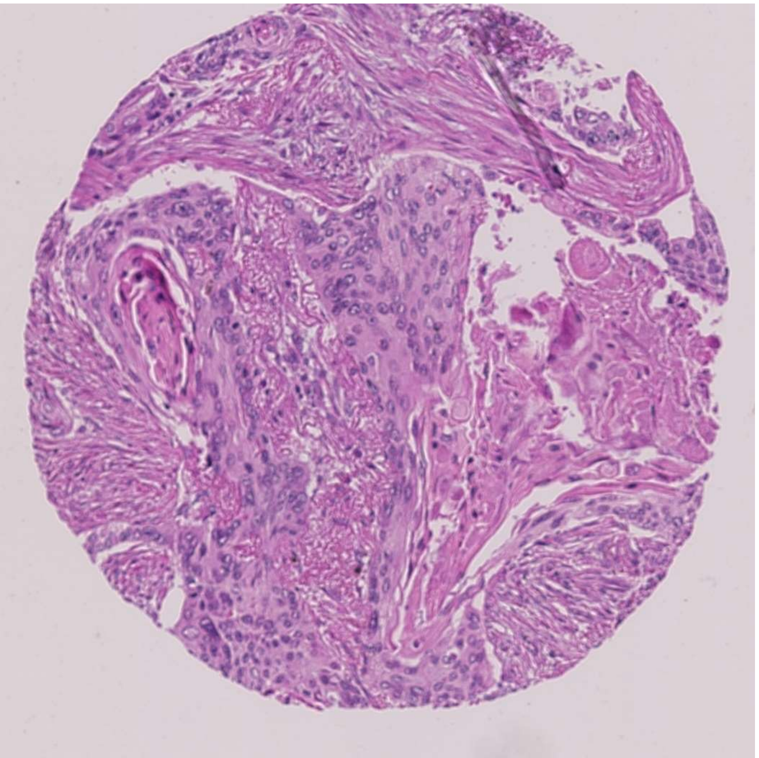

High 3

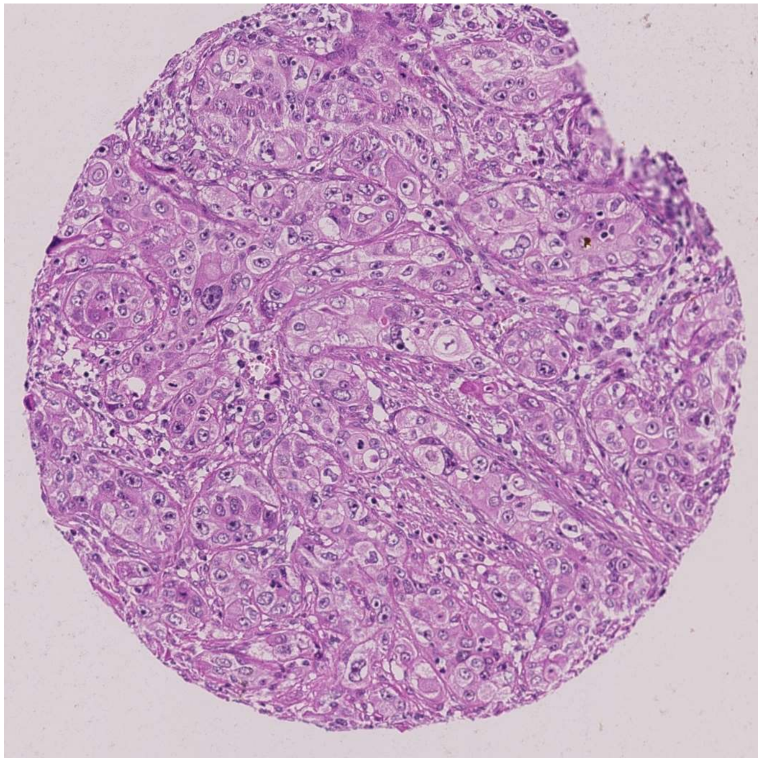

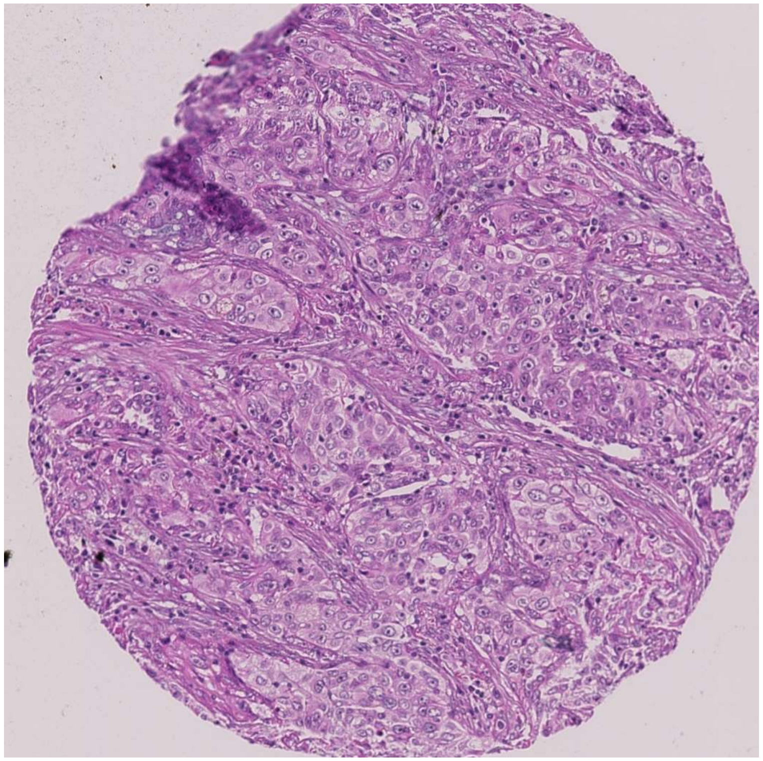

High 4

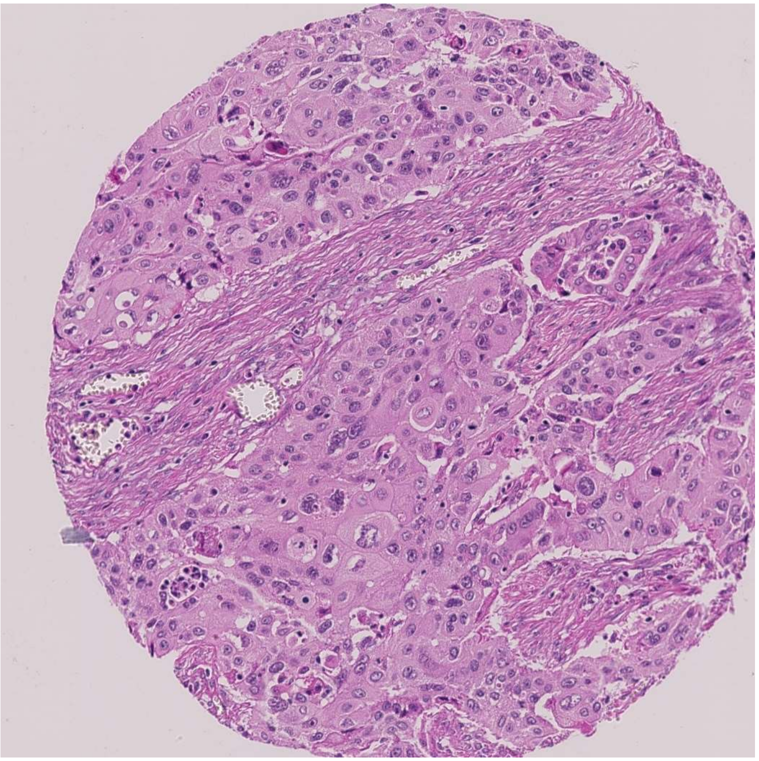

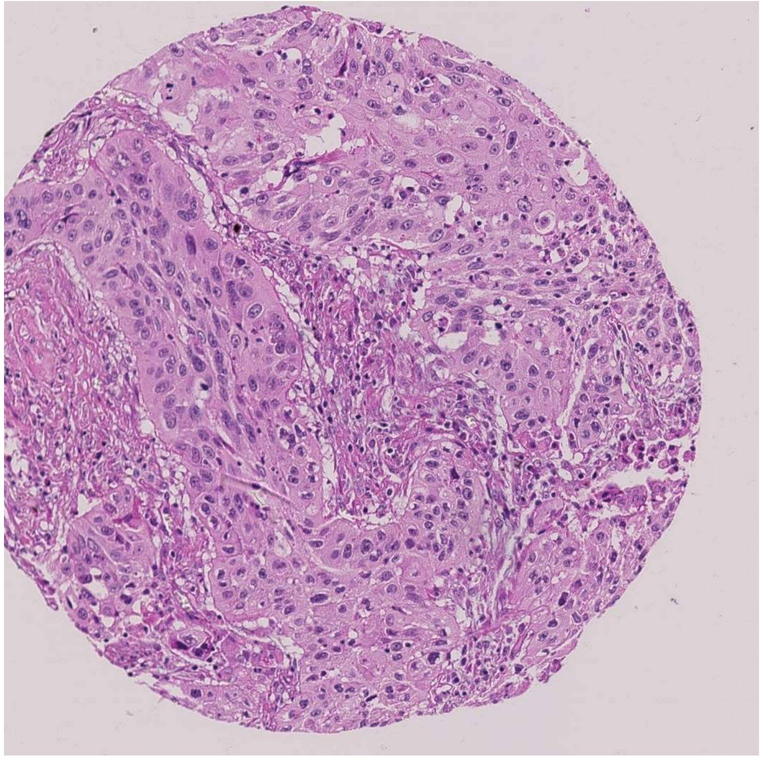

High 5

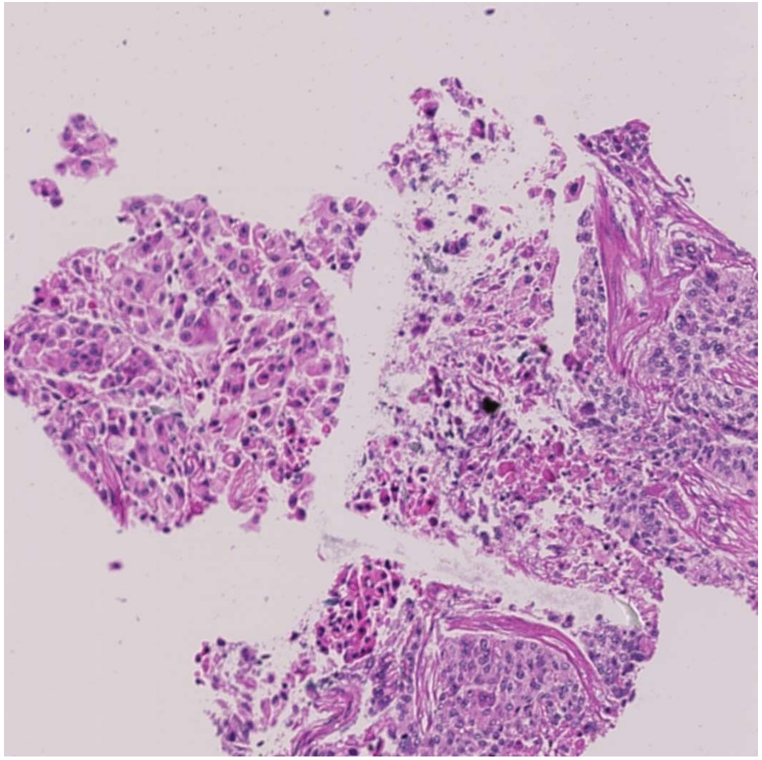

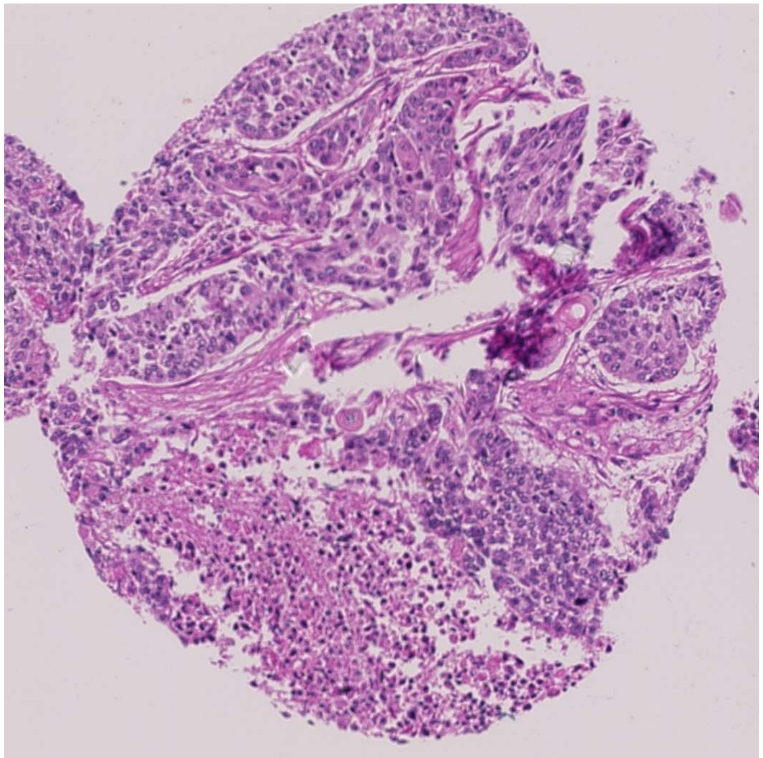

High 6

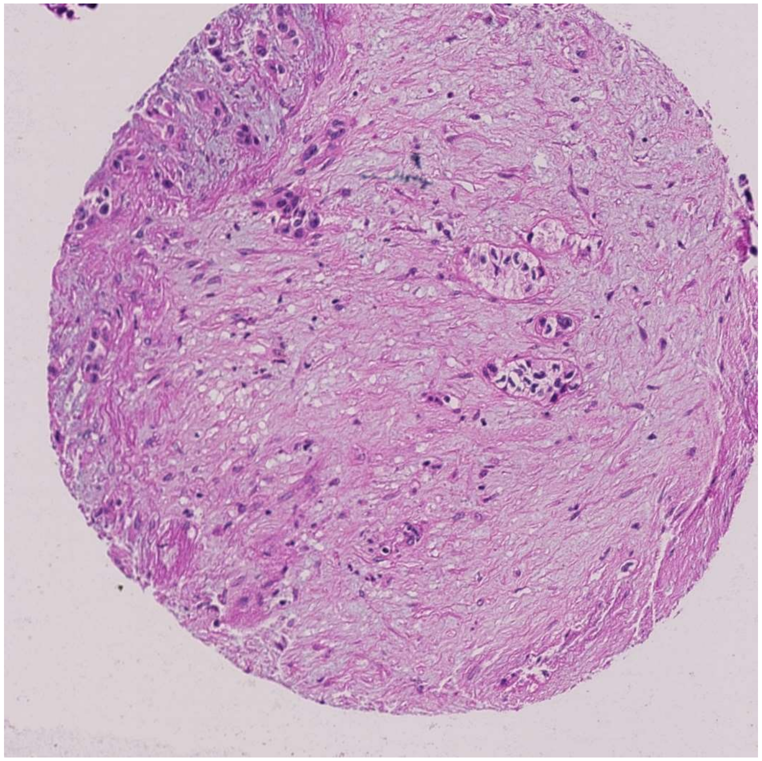

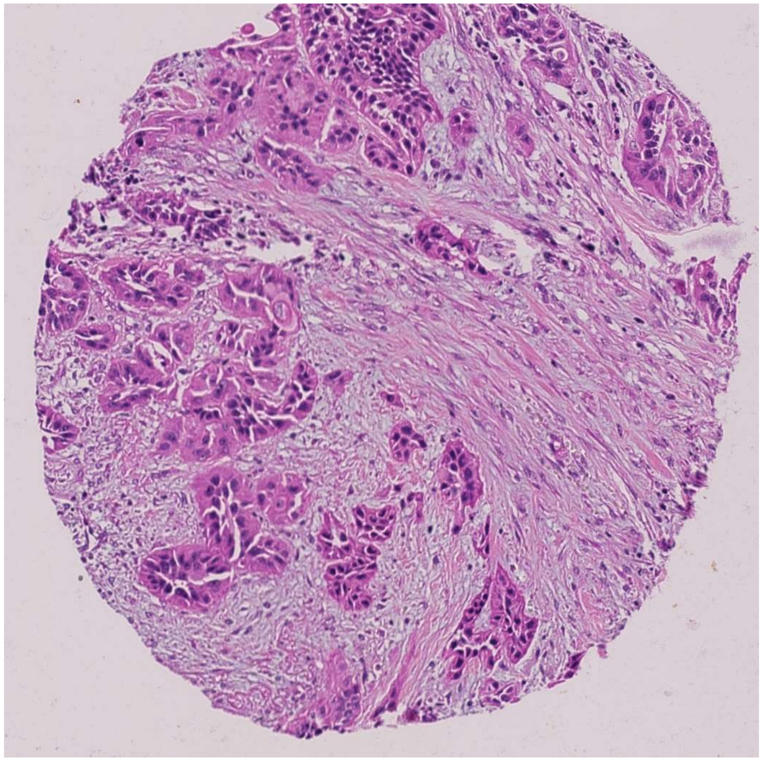

High 7

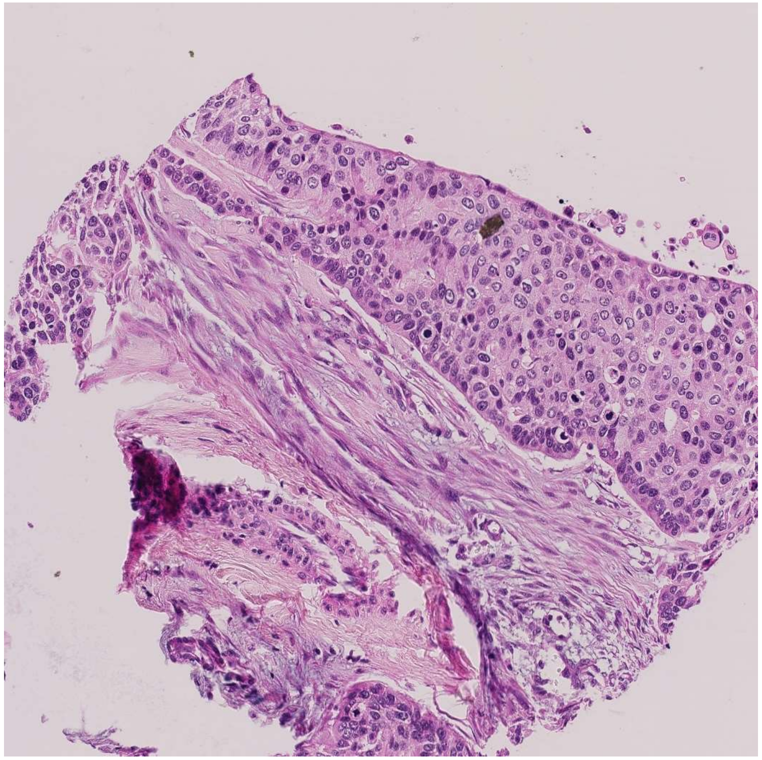

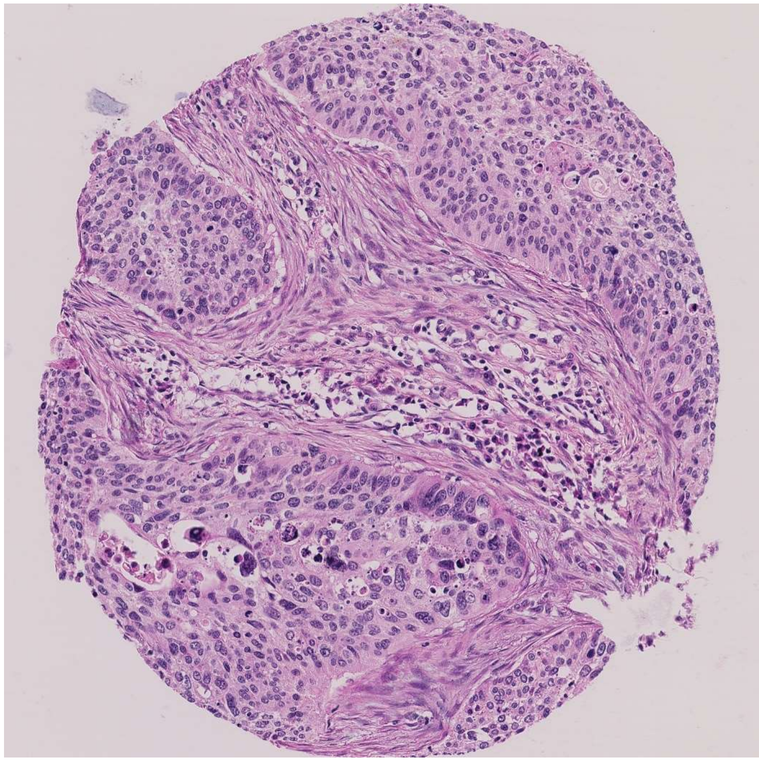

High 7

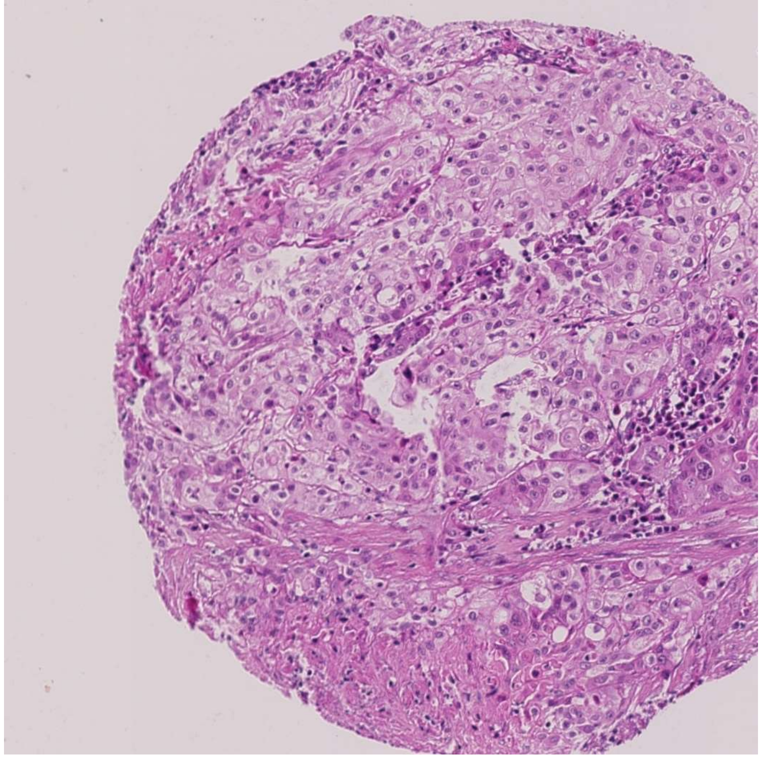

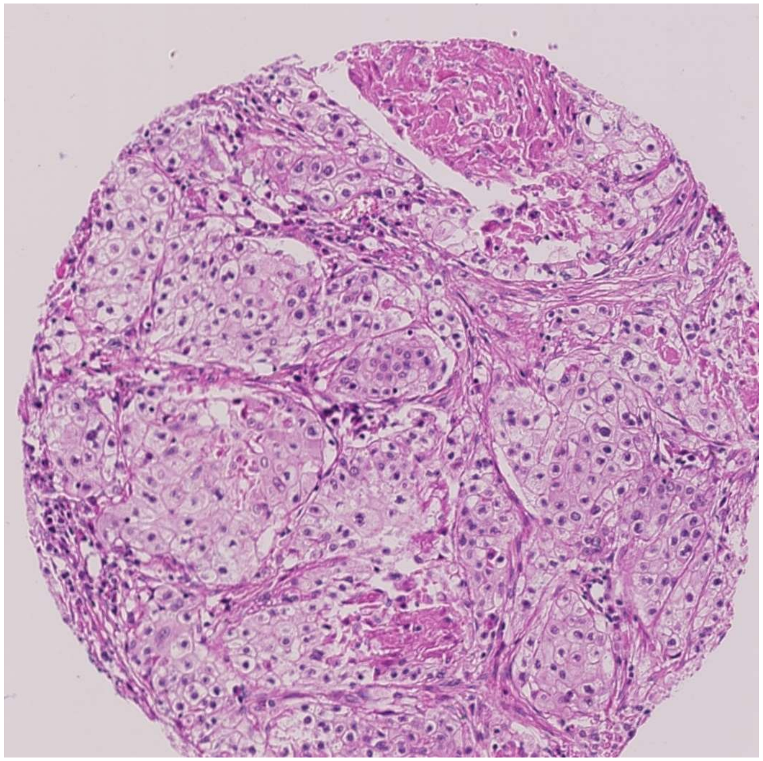

High 8

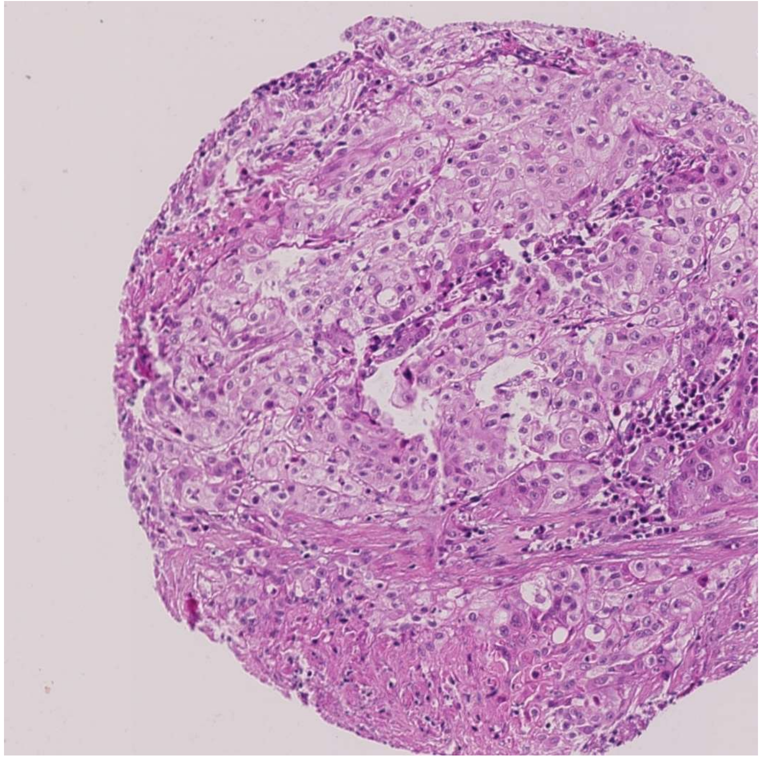

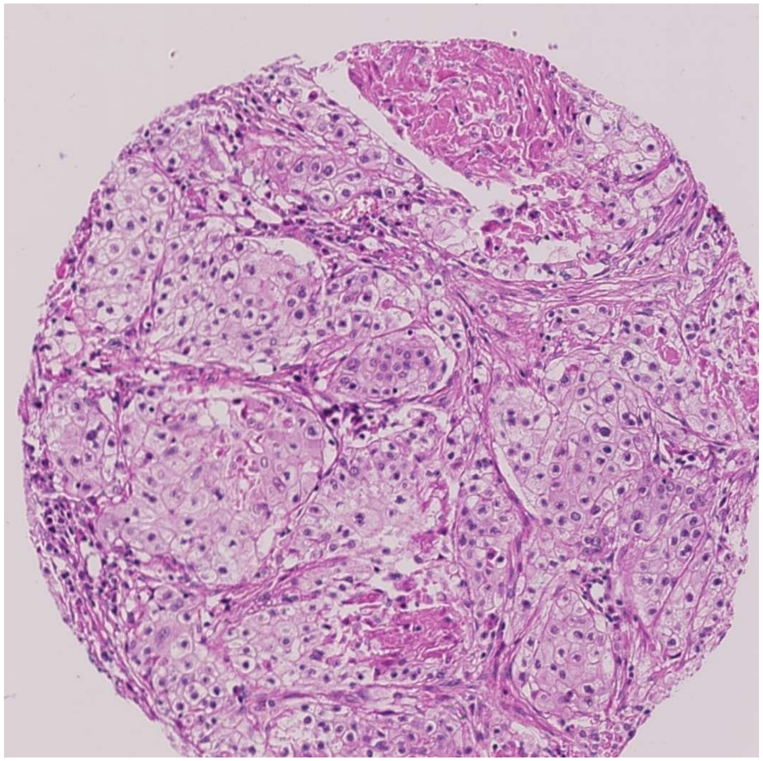

High 9

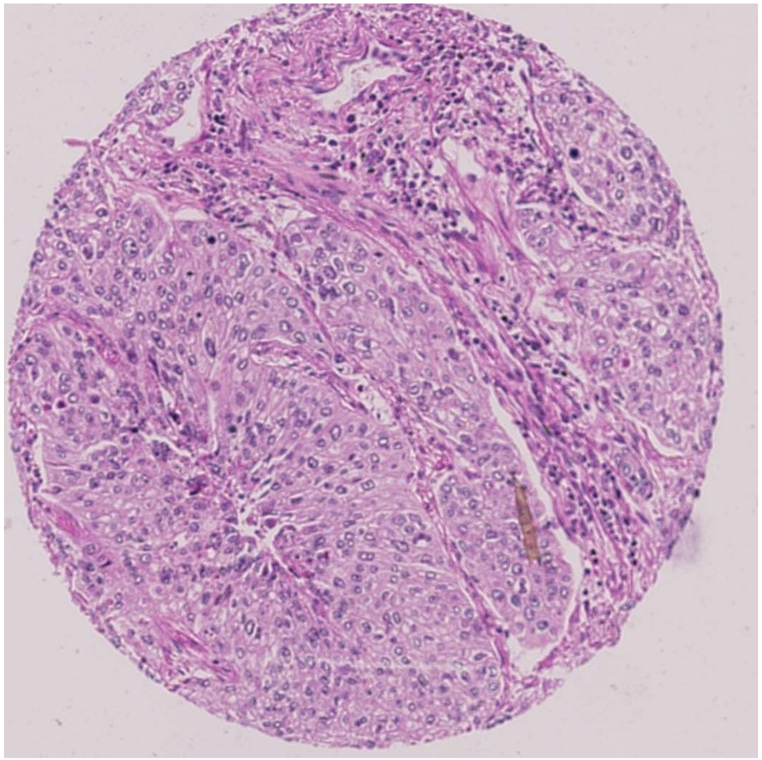

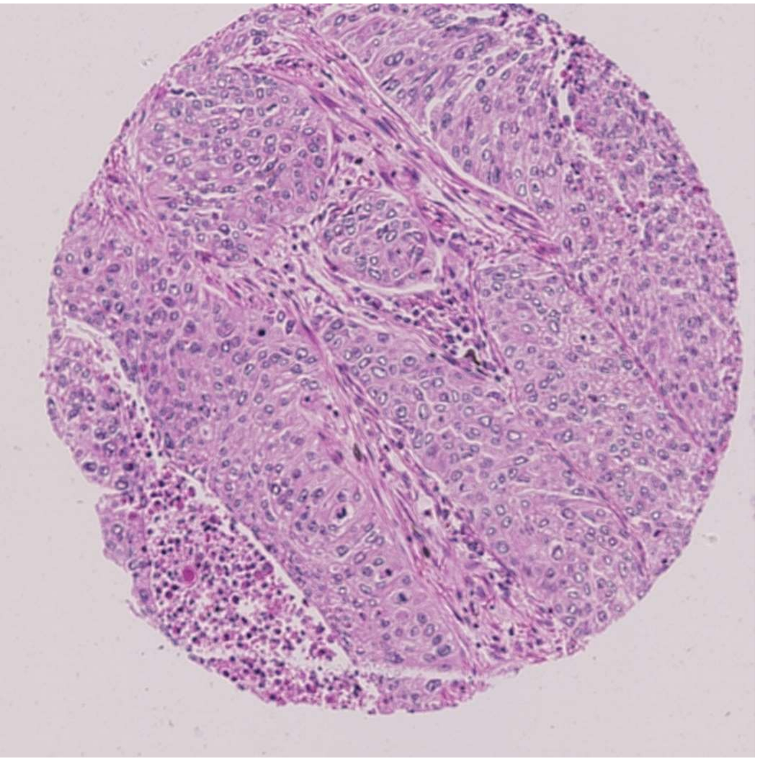

High 10

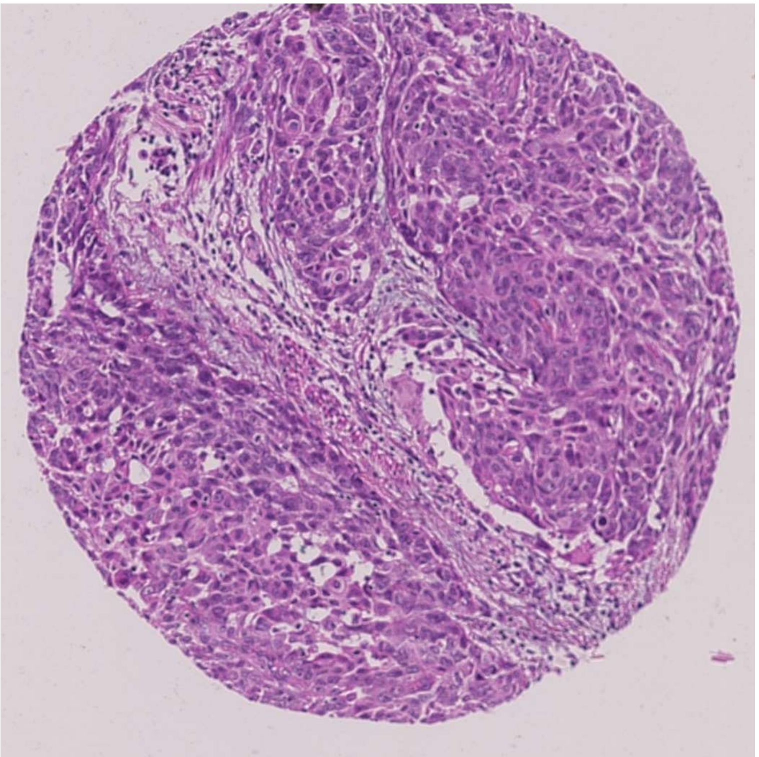

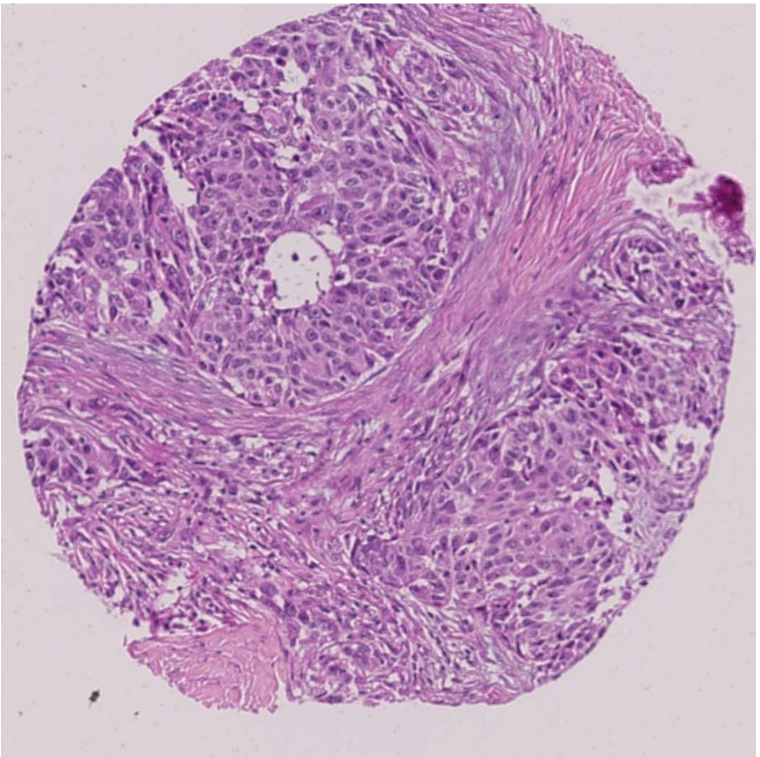

Low 1

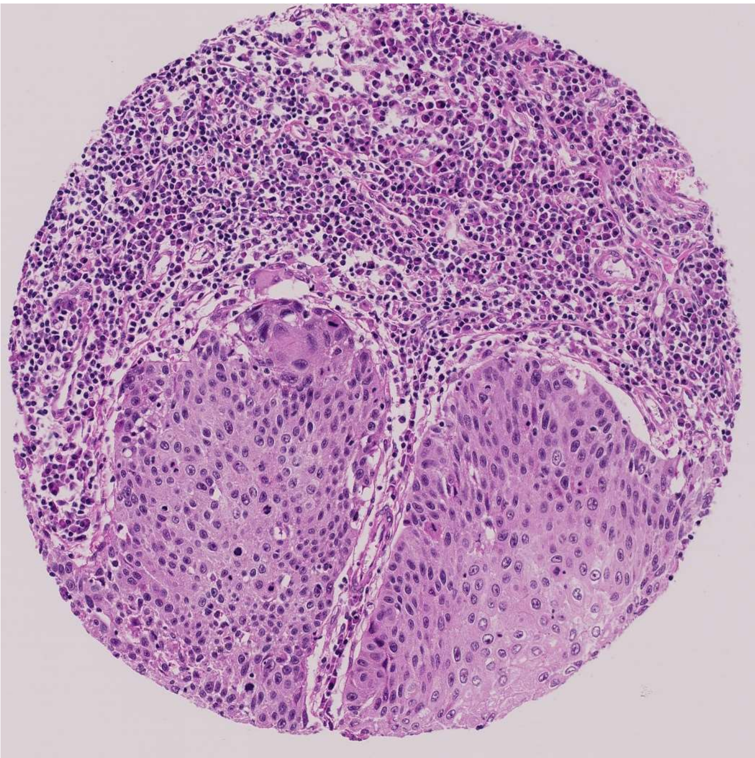

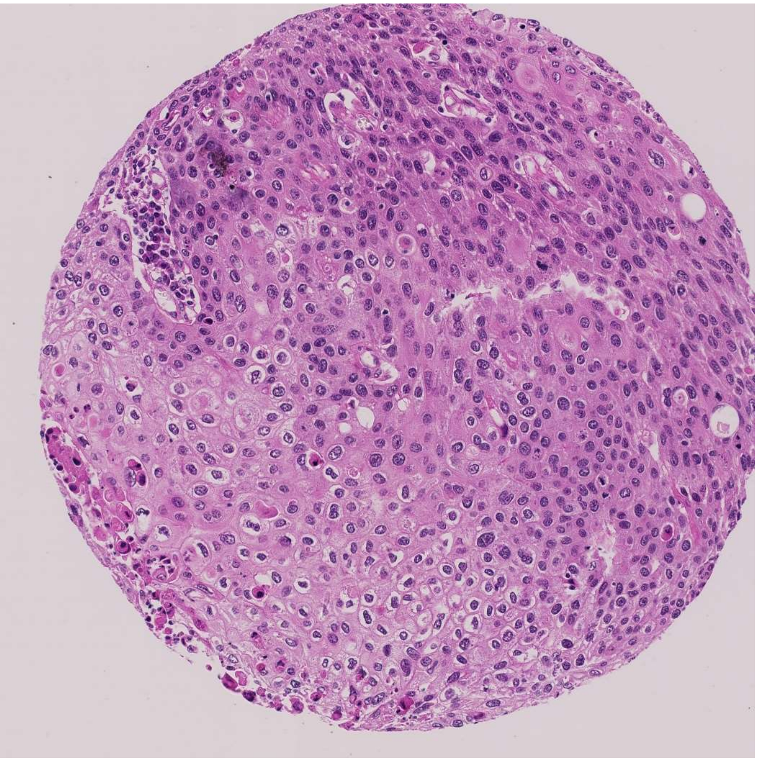

Low 2

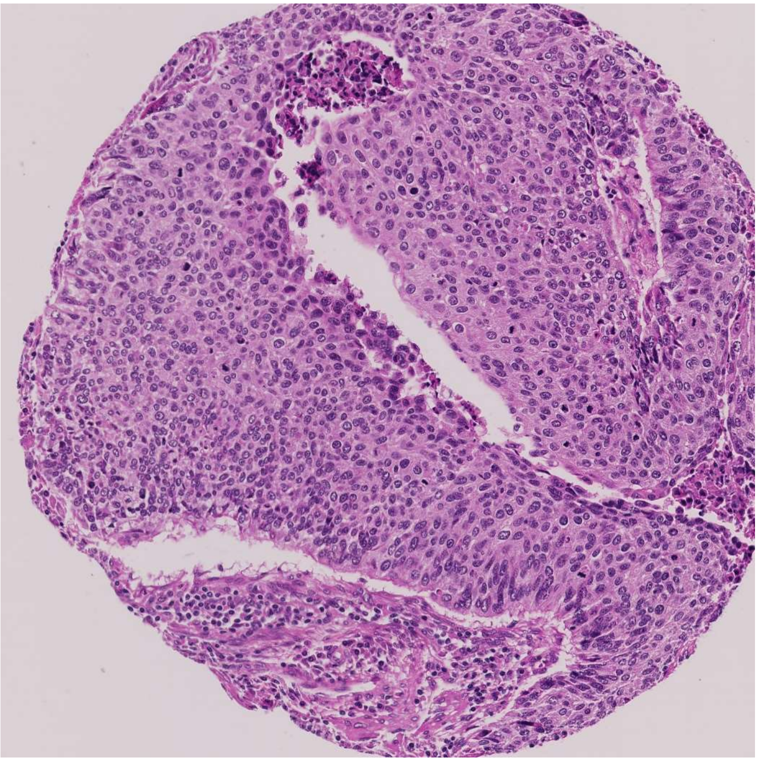

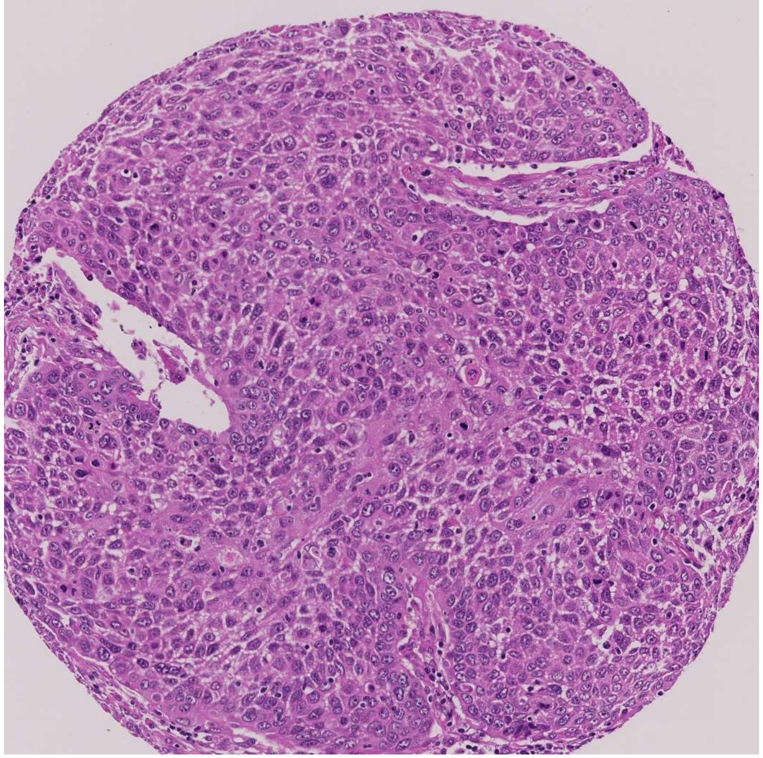

Low 3

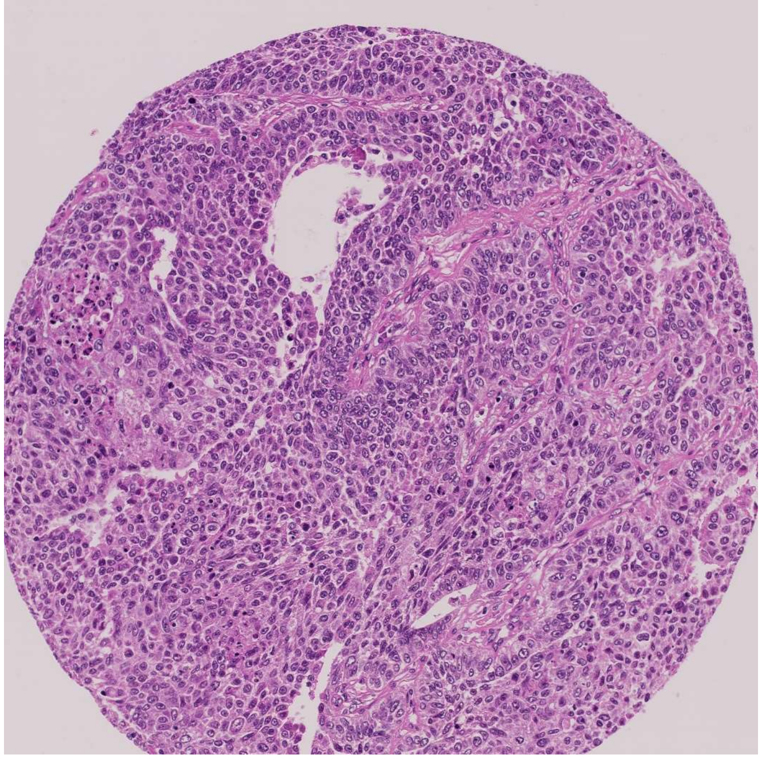

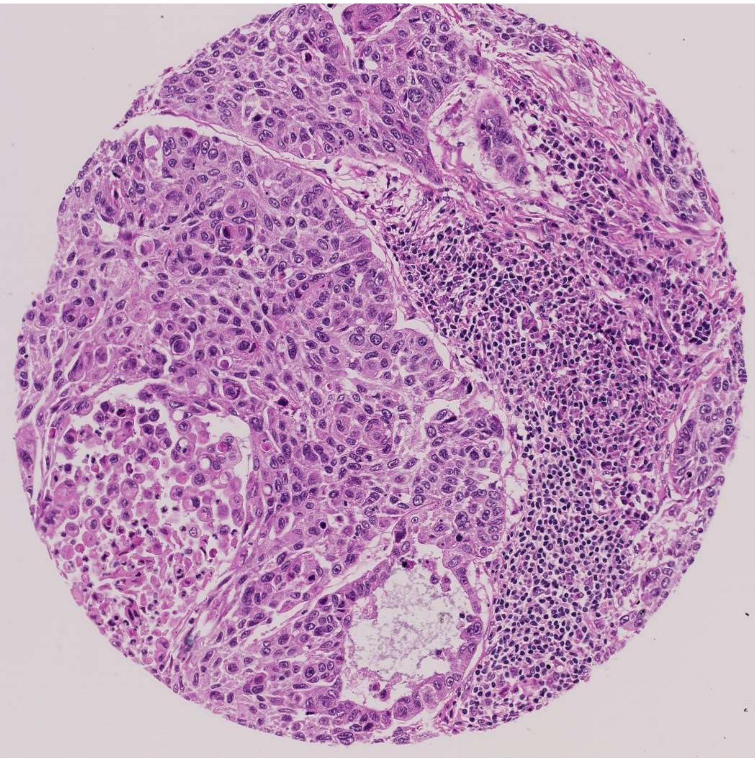

Low 4

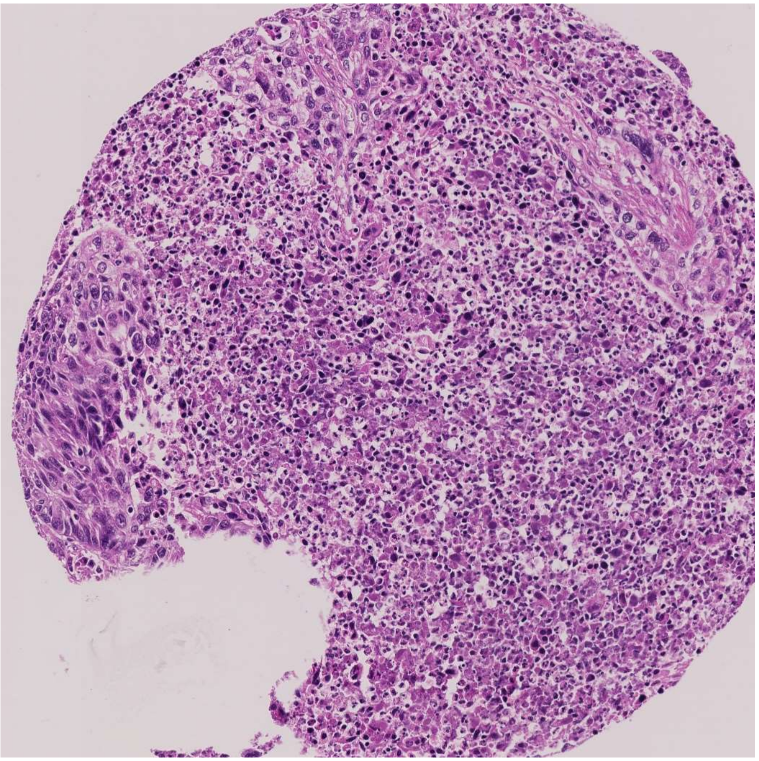

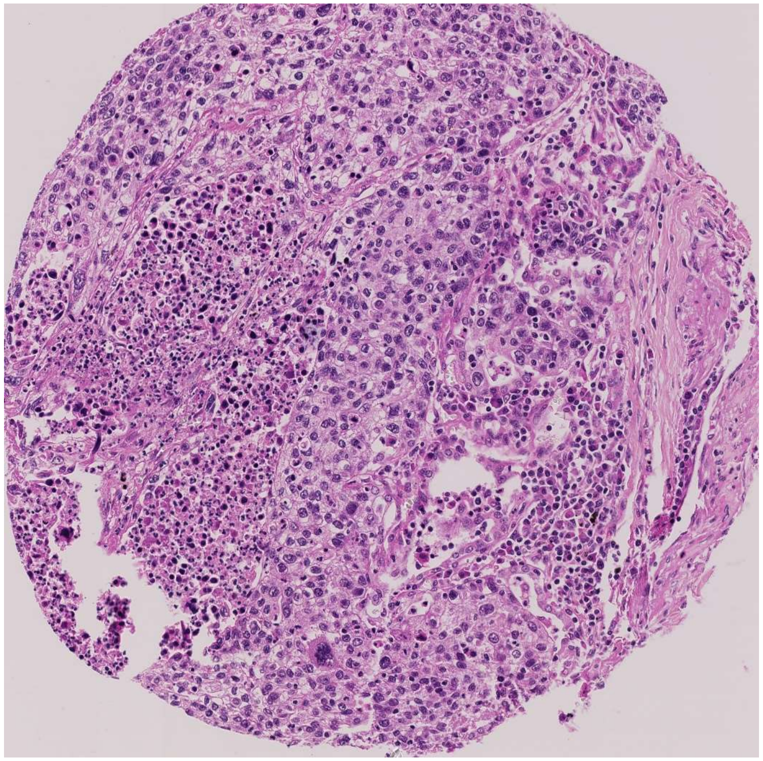

Low 5

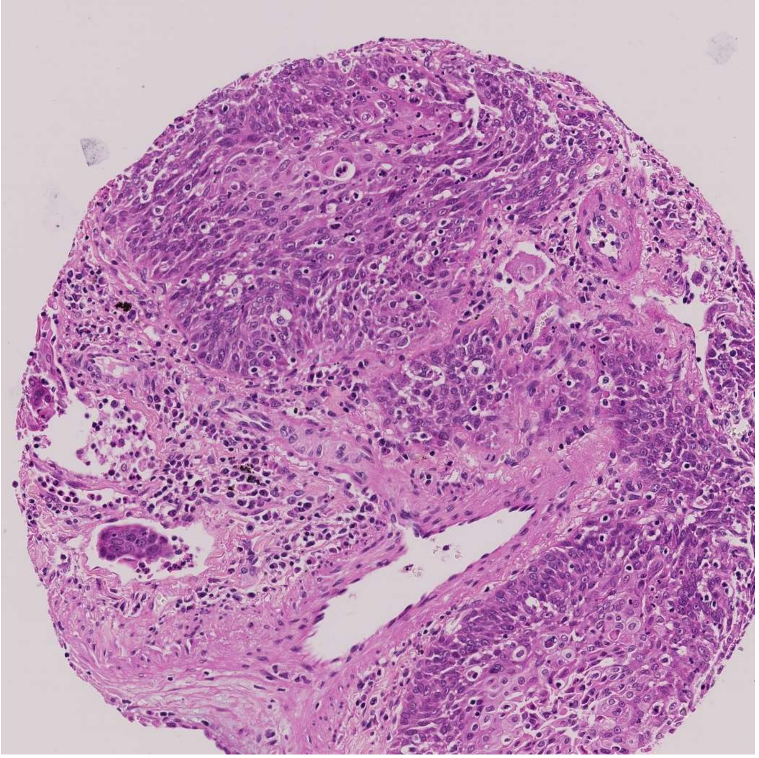

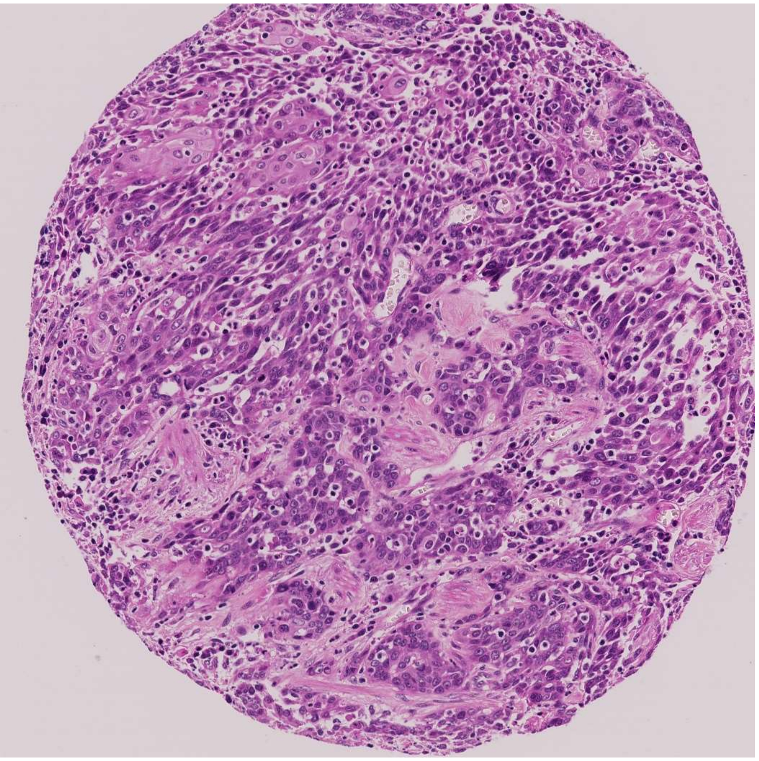

Low 6

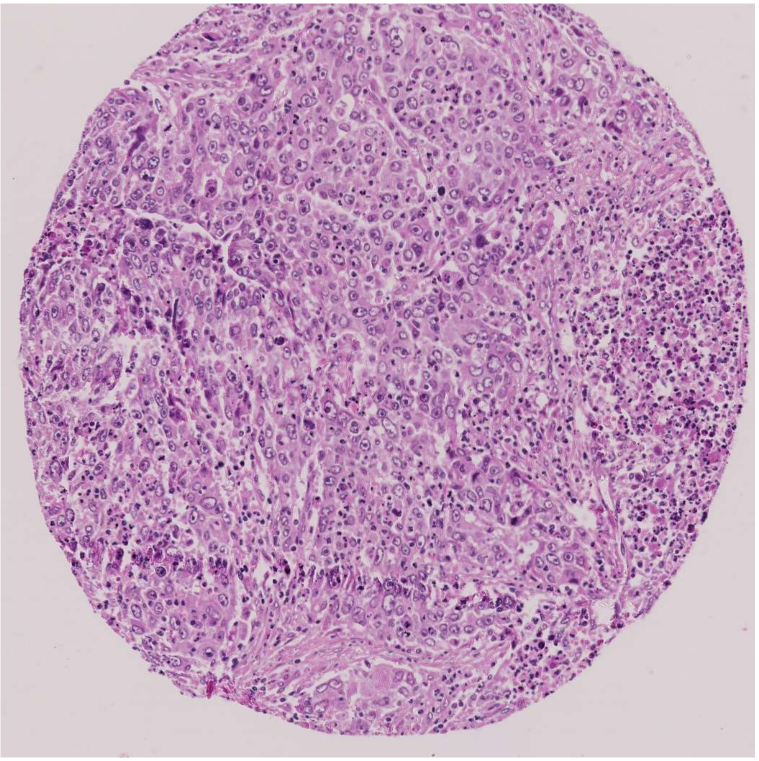 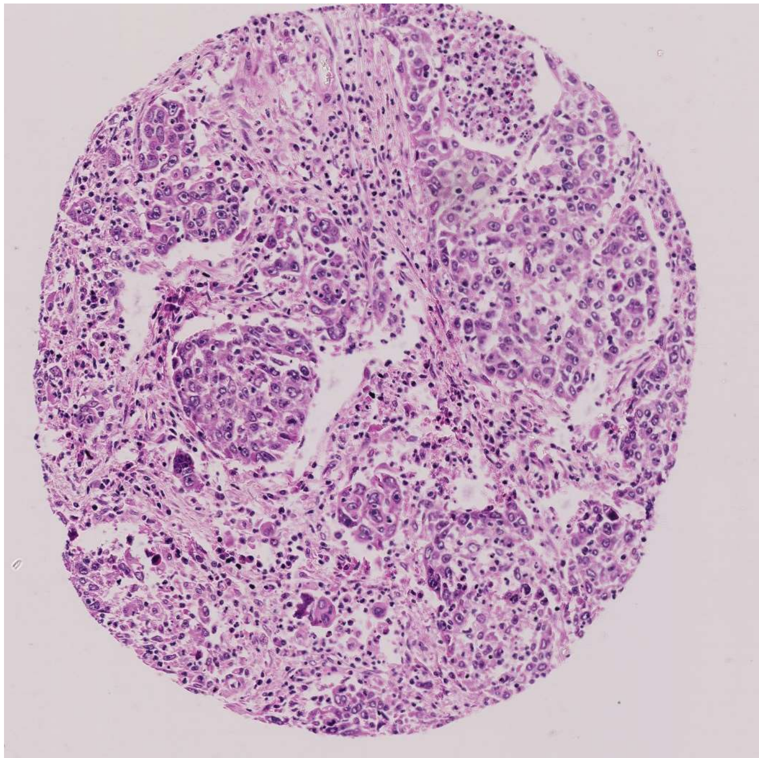

Low 7

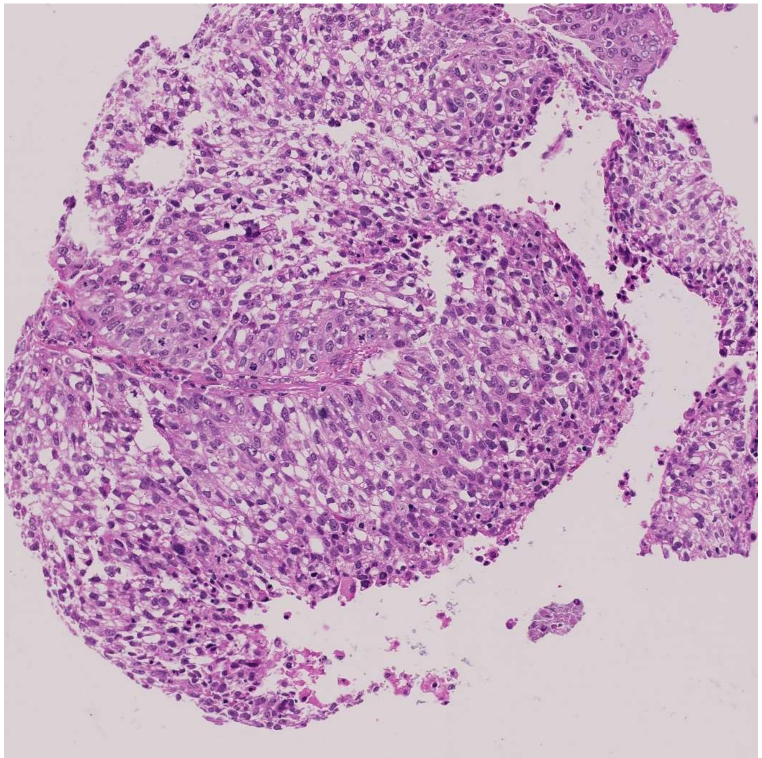

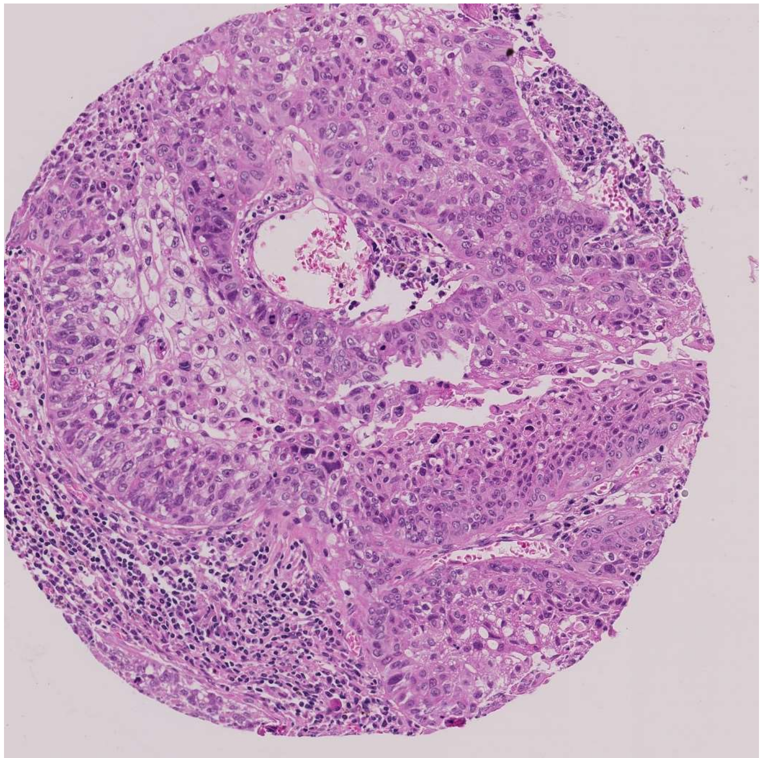

Low 8

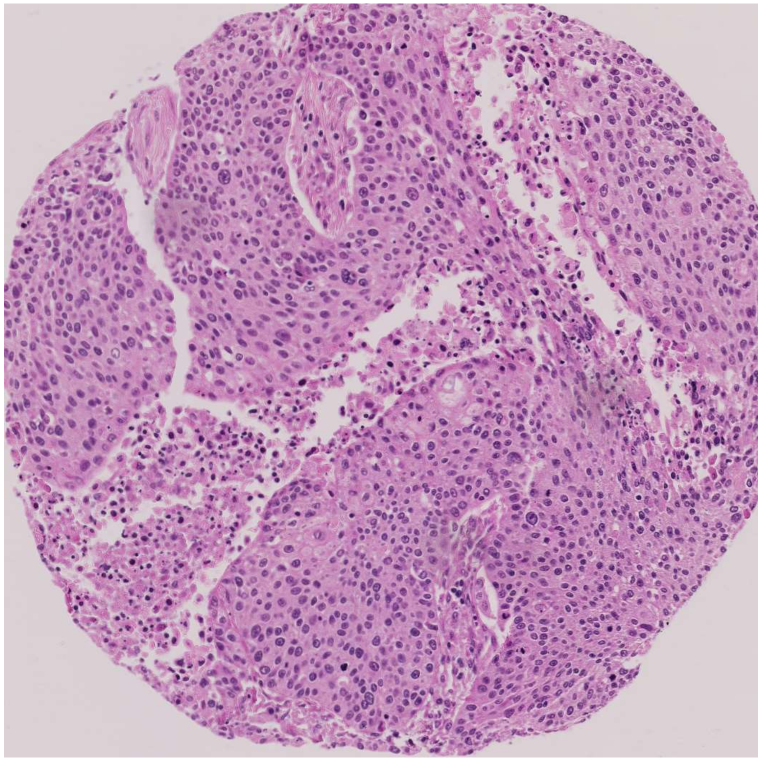

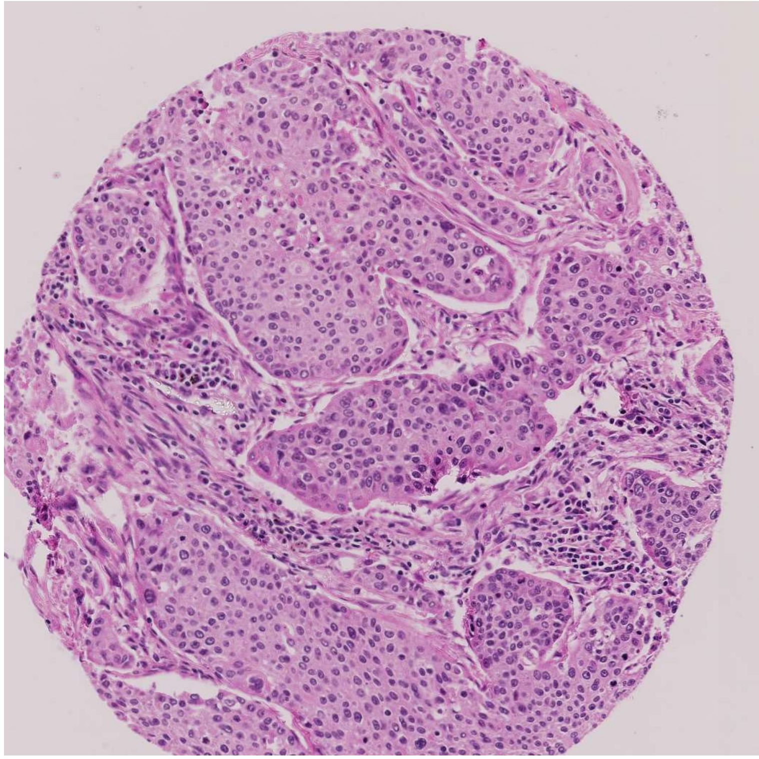

Low 9

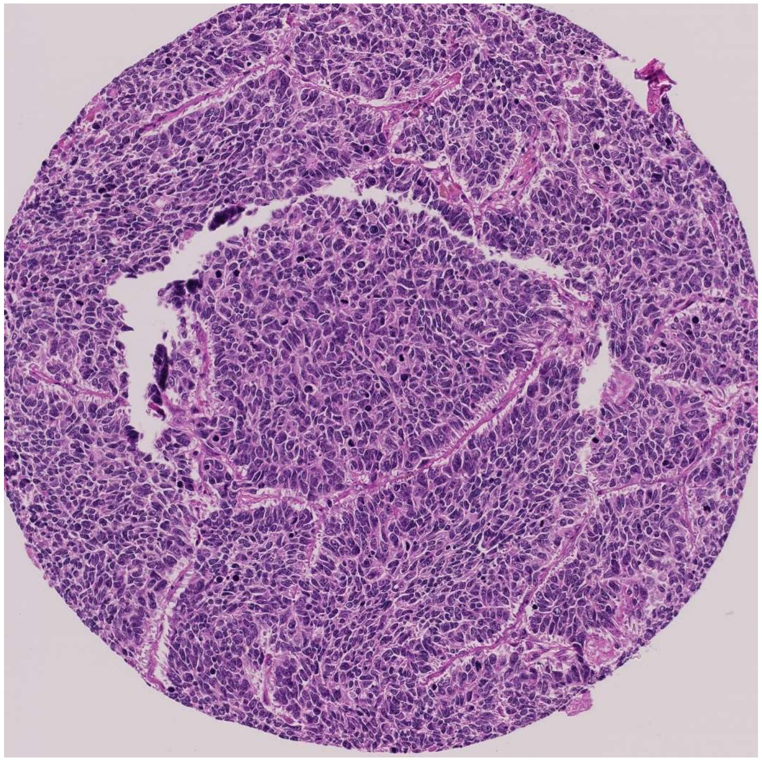

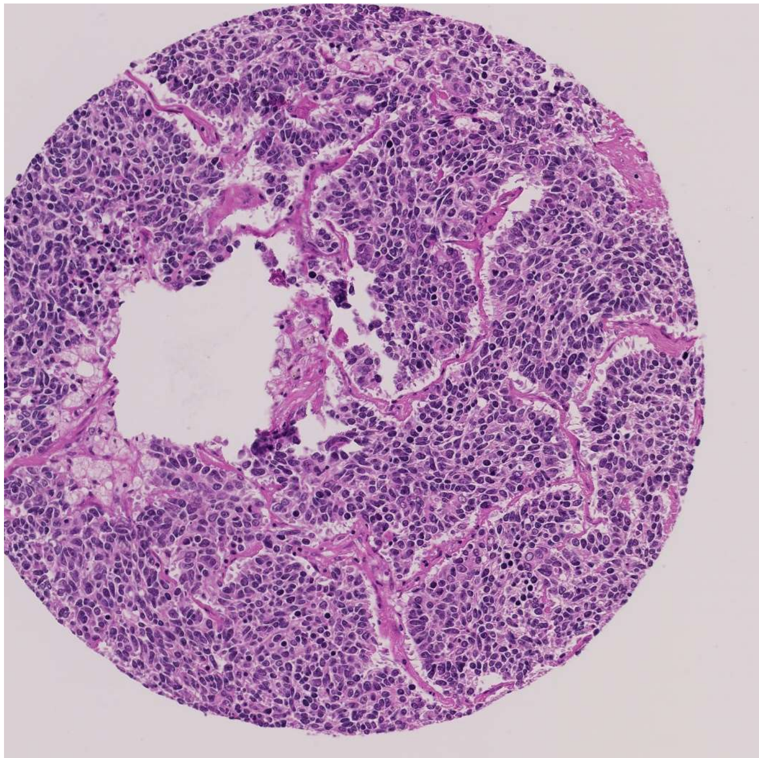

Low 10

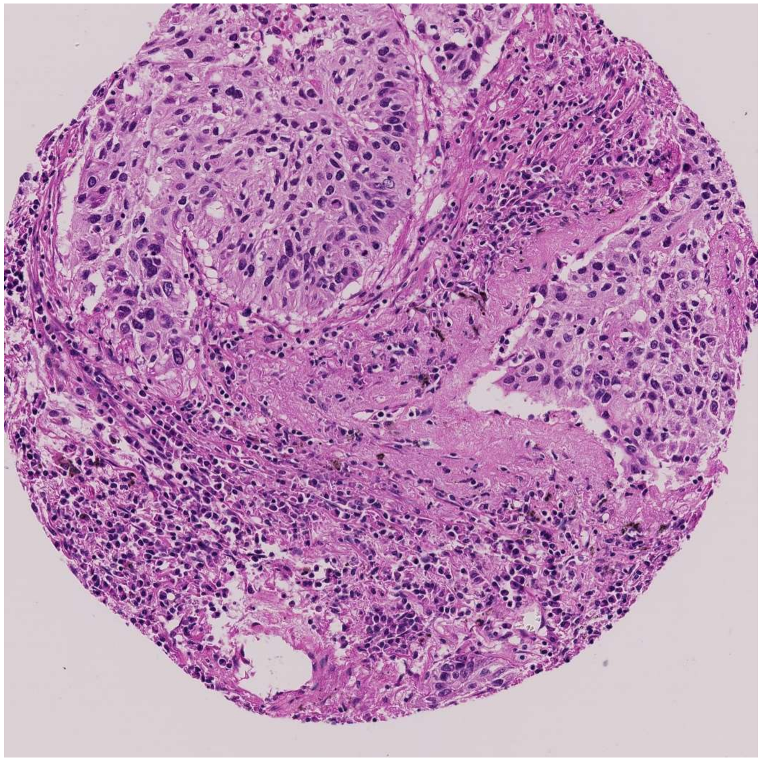

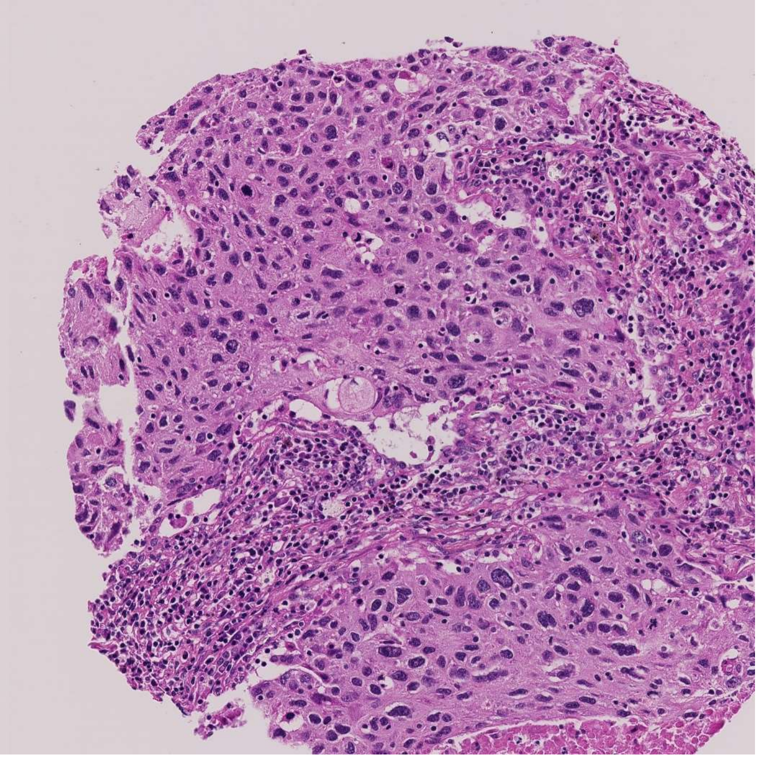

Summary of this feature

- **Shape index** measures the border length divided by four times the square root of the area of an object, and is a measure of the smoothness of an object
- **Stromal standard deviation shape index** measures the uniformity of the shape index feature across all nuclei in the stroma of an image
- As one might imagine:
  - A stroma rich in lymphocytes would be more uniform and have a **lower** St Dev
  - A stroma rich in spindle cells cut at different angles with respect to the plane of section would be less uniform have a **high** St Dev
- Consistent with this, the highest 10 cases have relatively less **stromal chronic inflammation** than lowest 10 by manual scoring (see next slide)

Inflammation (manual score)

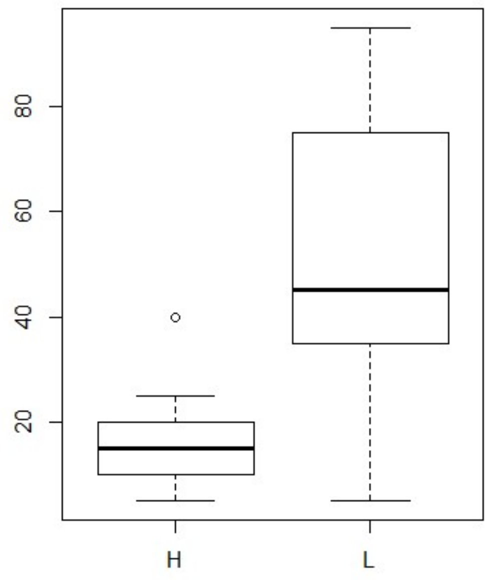

- L=lowest 10 cases
- H=highest 10 cases
- P=0.00109

Survival

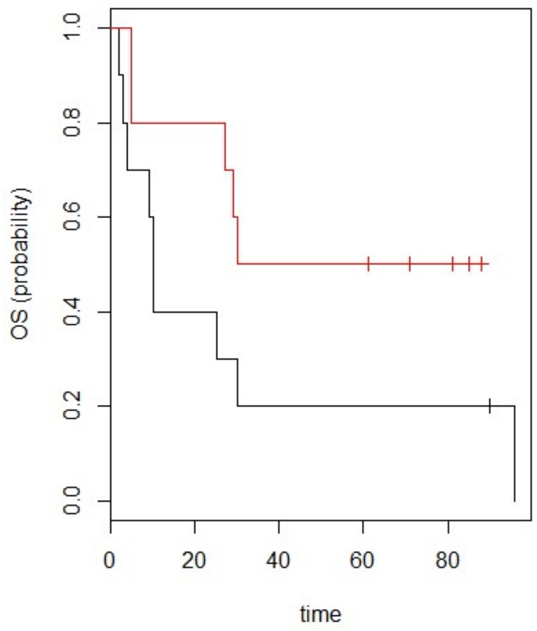

- Red = lowest 10
- Black = highest 10
- P=0.094

Stroma.Nucleus.Std..Dev..Density

What is Stroma.Nucleus.Std..Dev..Density?

The Density feature describes the distribution in space of the pixels of an image object. In Definiens Developer XD 2.0.4 the most “dense” shape is a square; the more an object is shaped like a filament, the lower its density. The density is calculated by the number of pixels forming the image object divided by its approximated radius, based on the covariance matrix.

Parameters

- $\sqrt{\#P_v}$ is the diameter of a square object with $\#P_v$ pixels
- $\sqrt{VarX + VarY}$ is the diameter of the ellipse

Expression

$$\frac{\sqrt{\#P_v}}{1+\sqrt{VarX+VarY}}$$

Feature Value Range

$[0, \text{depending on shape of image object}]$

High 1

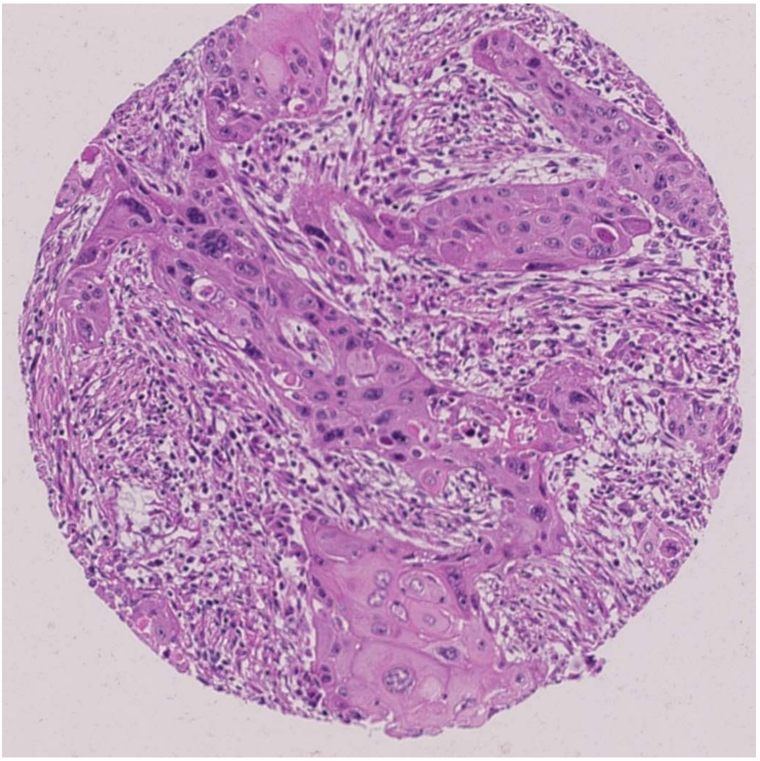

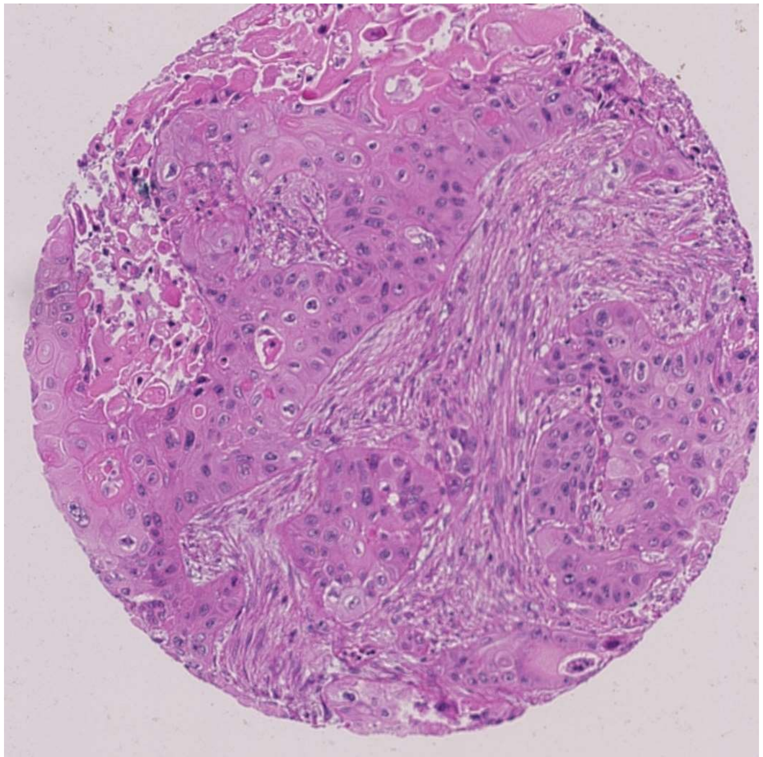

High 2

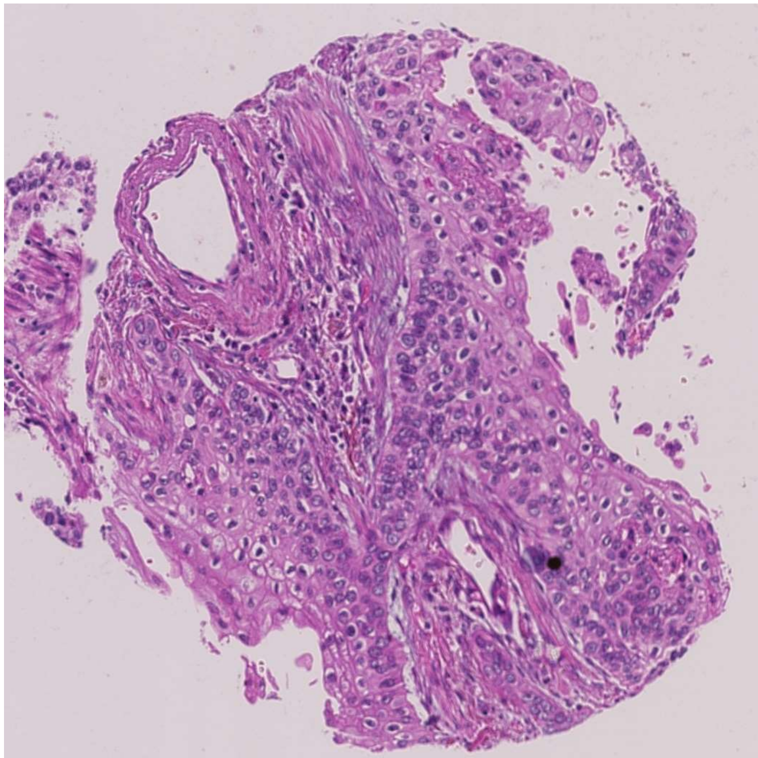

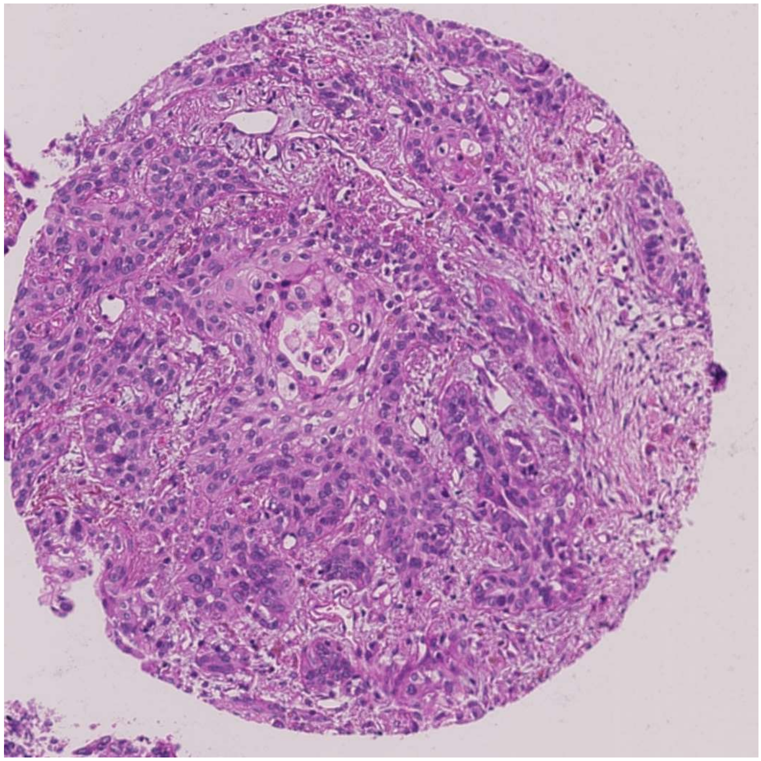

High 3

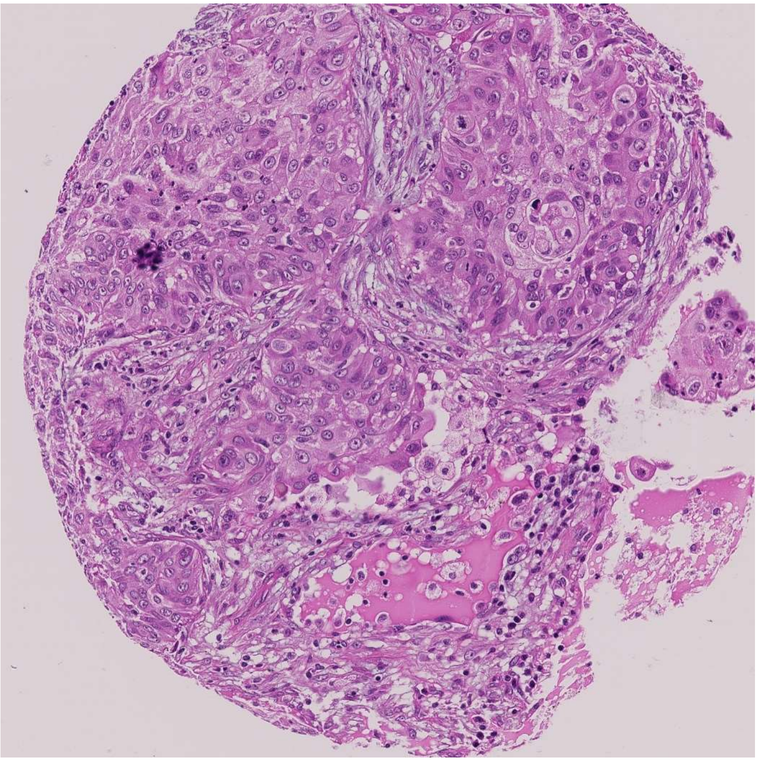

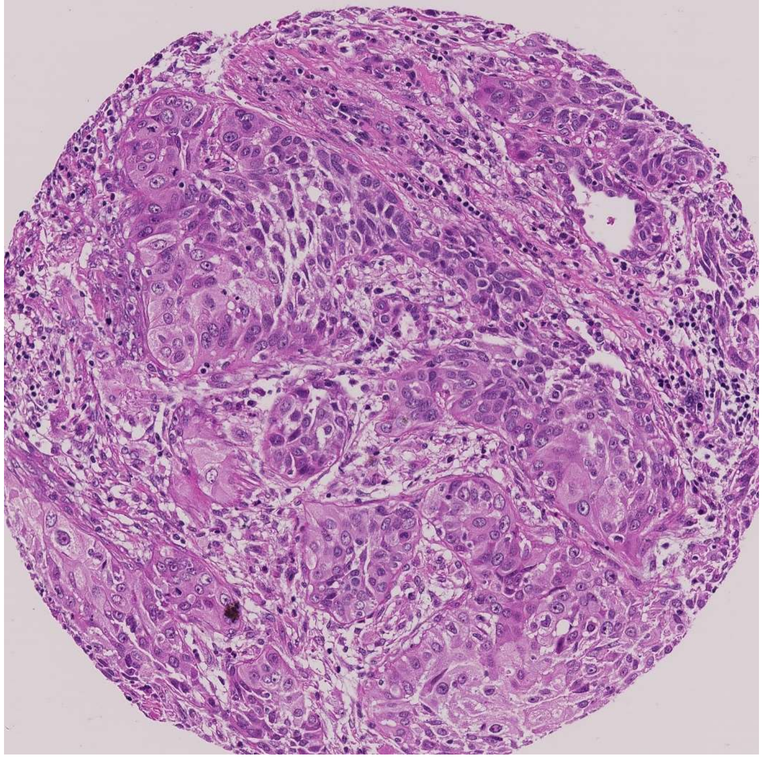

High 4

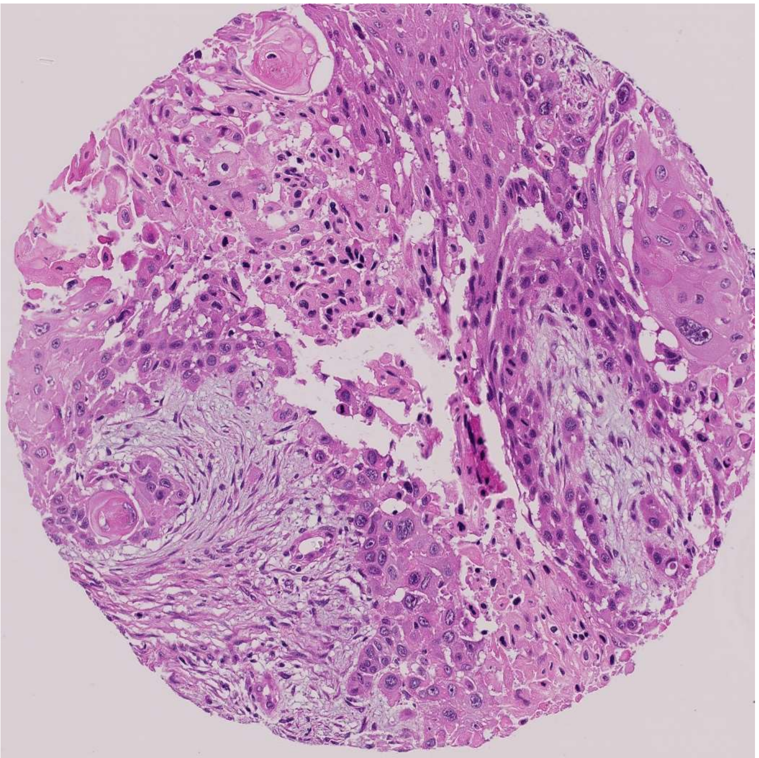

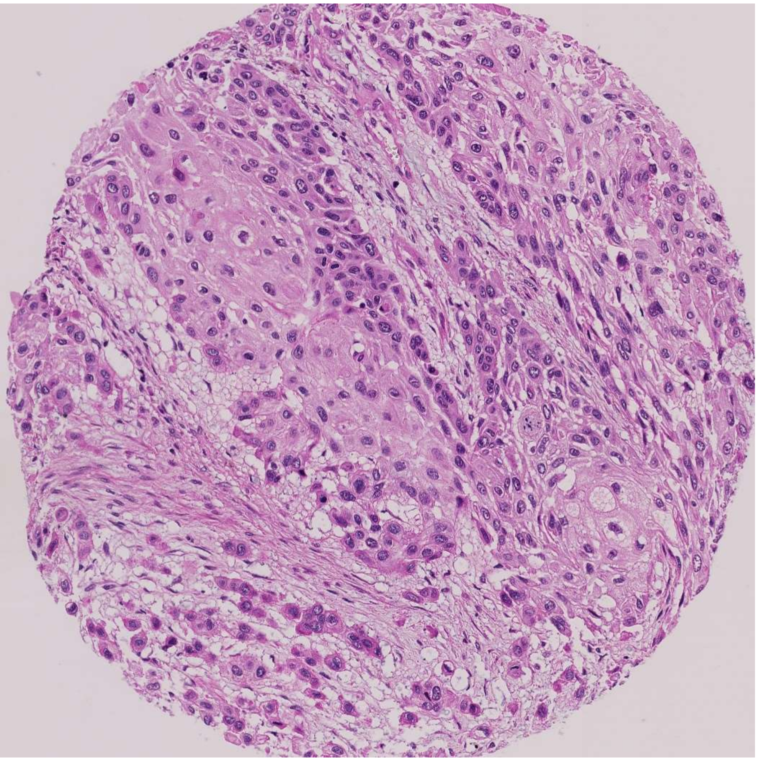

High 5

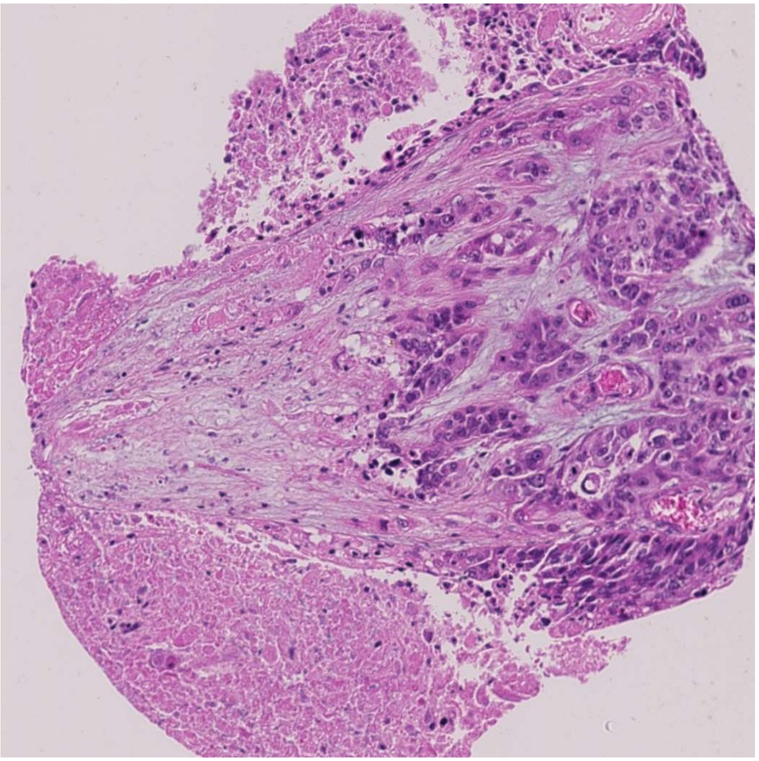

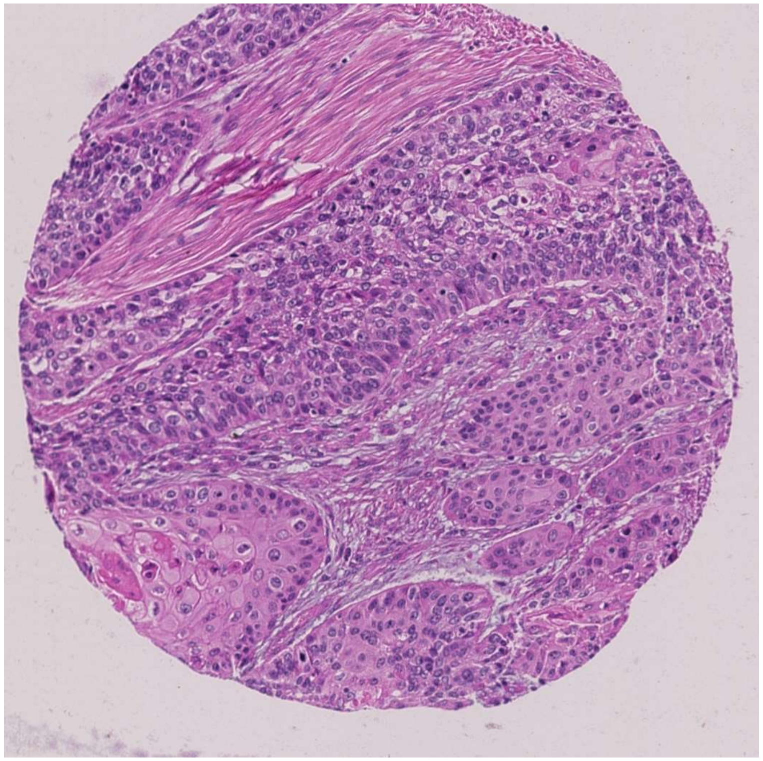

High 6

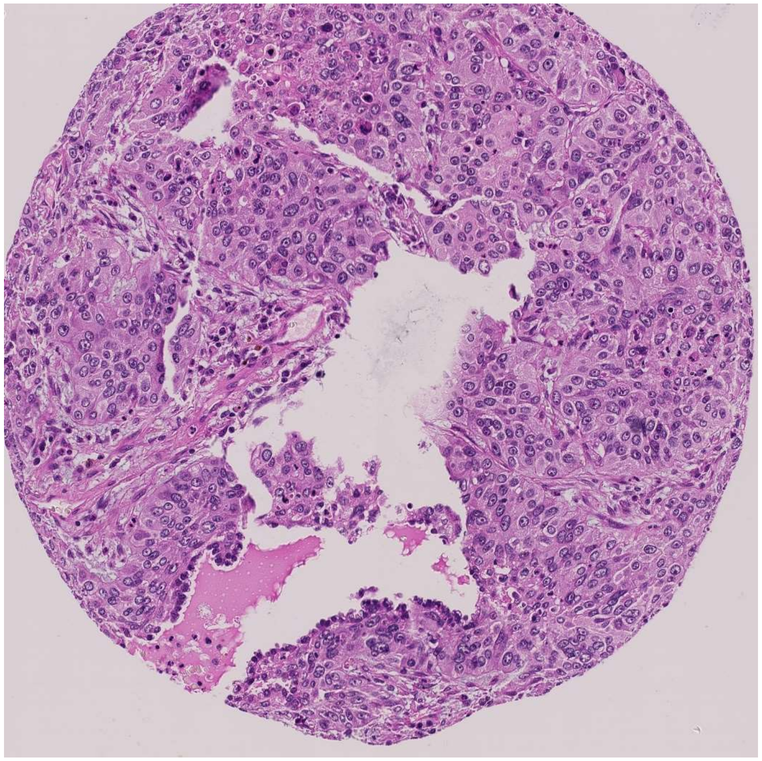

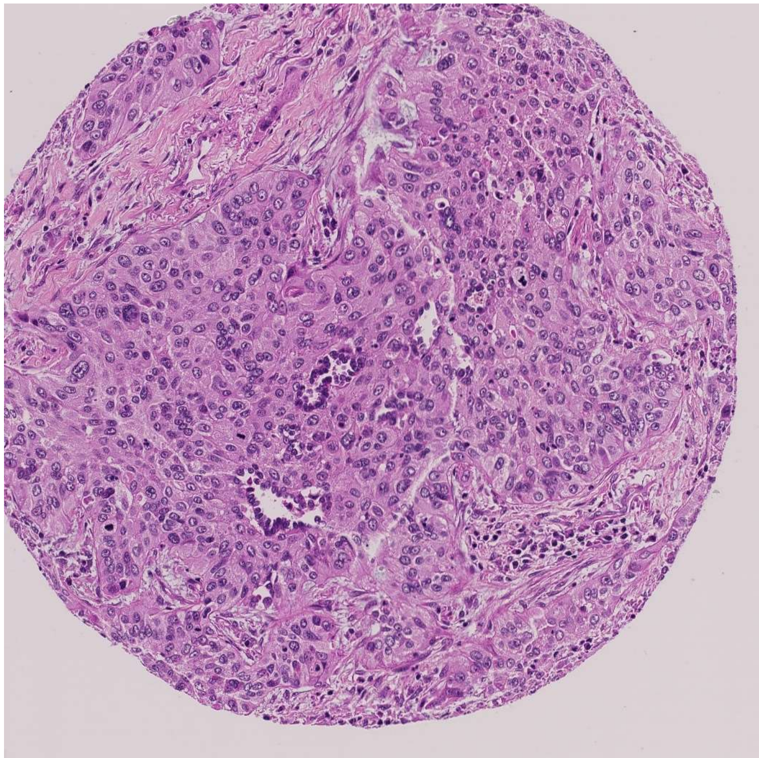

High 7

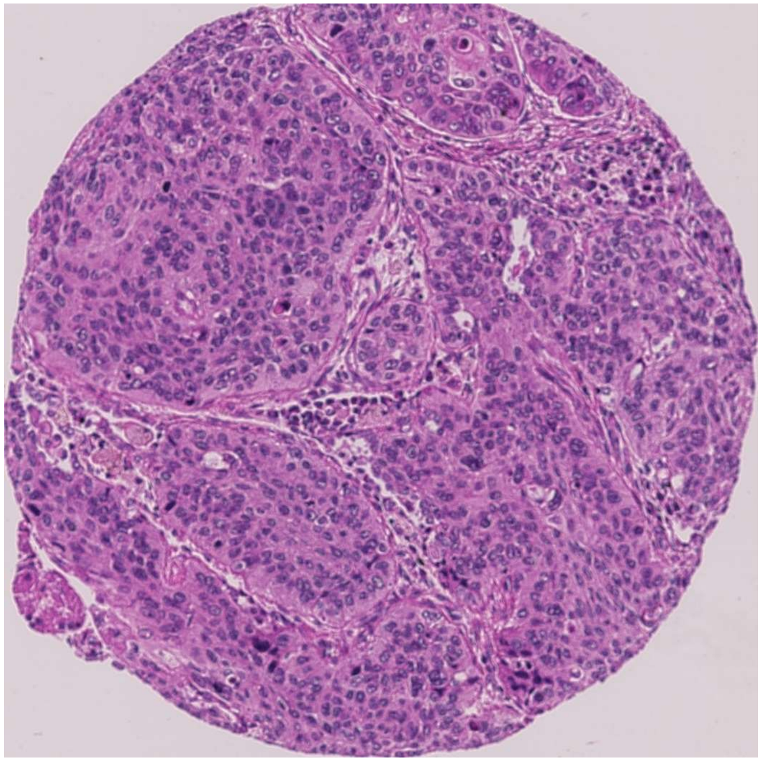

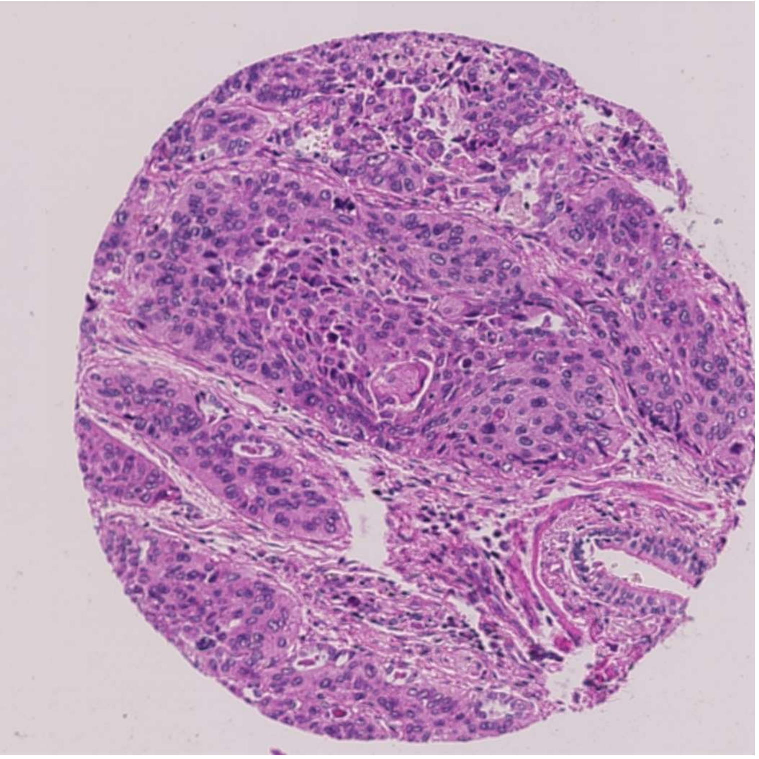

High 8

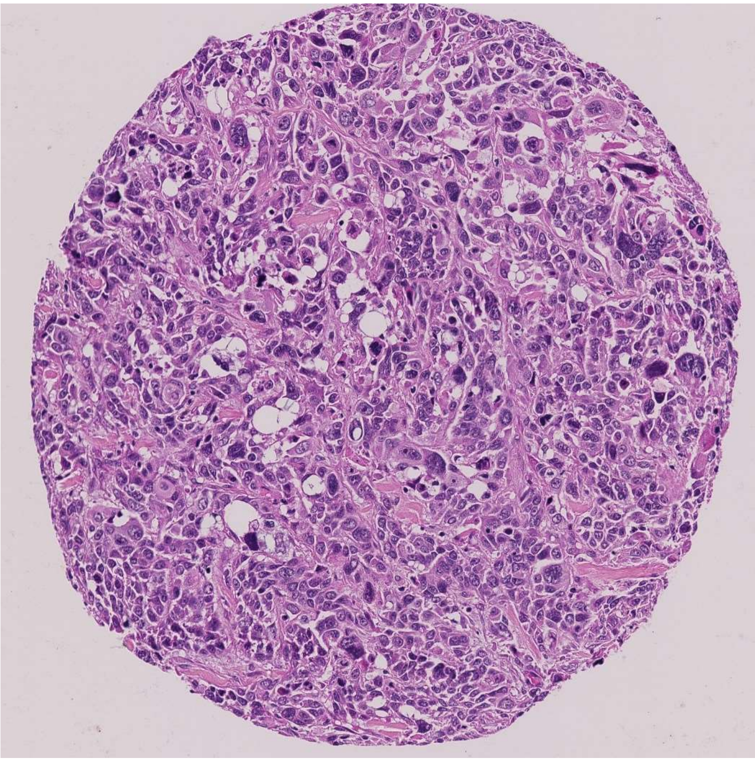

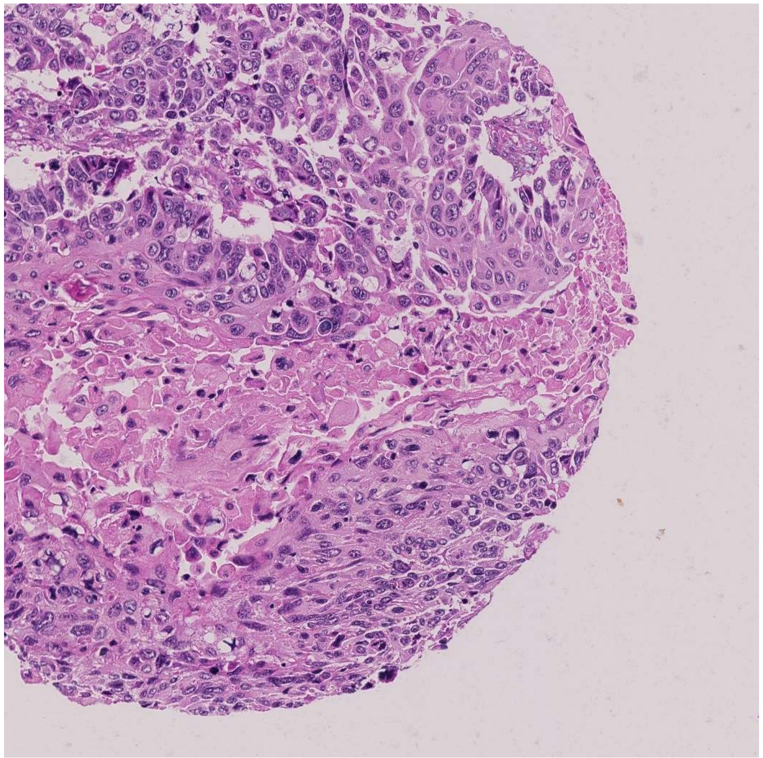

High 9

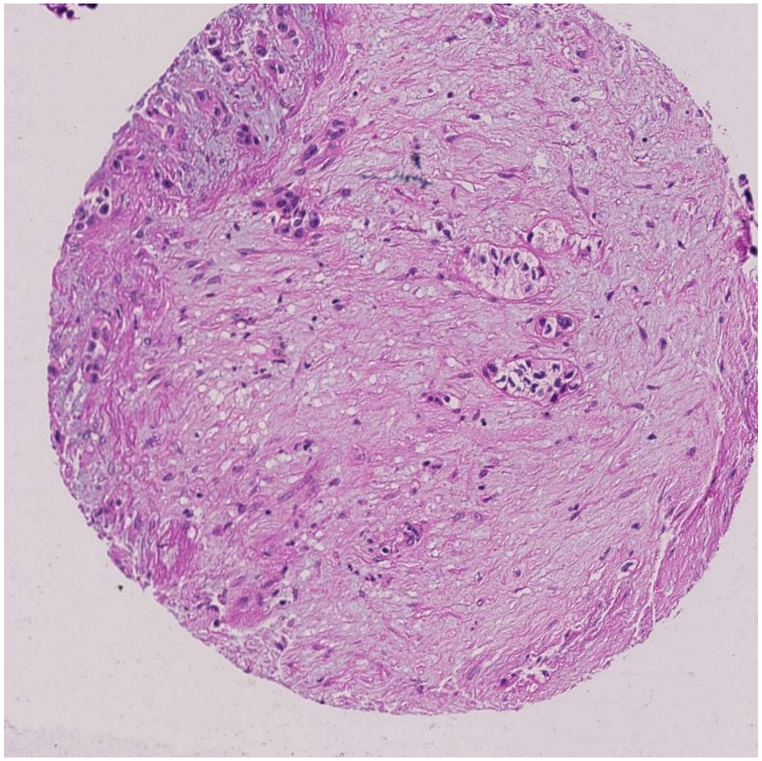

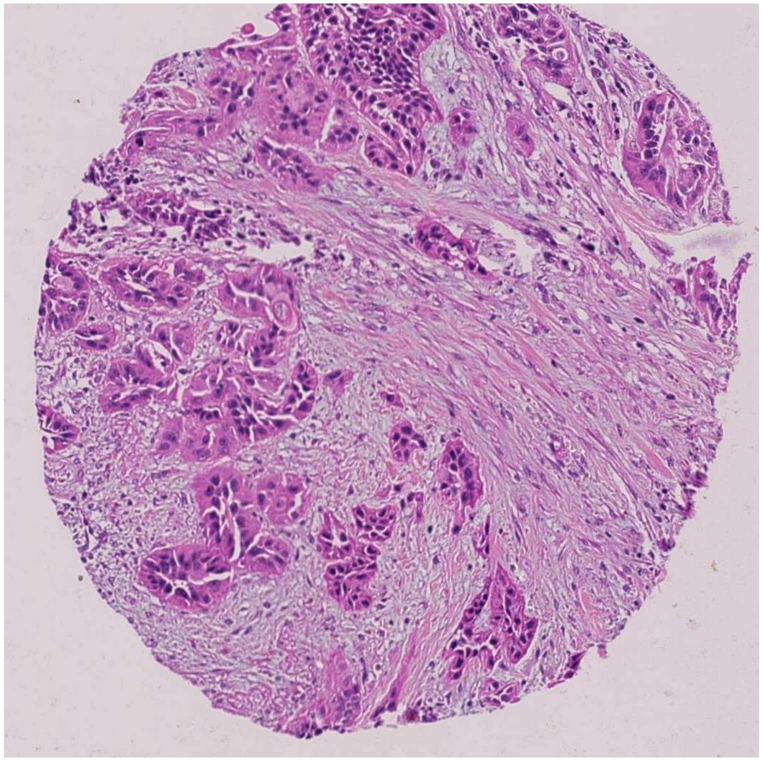

High 10

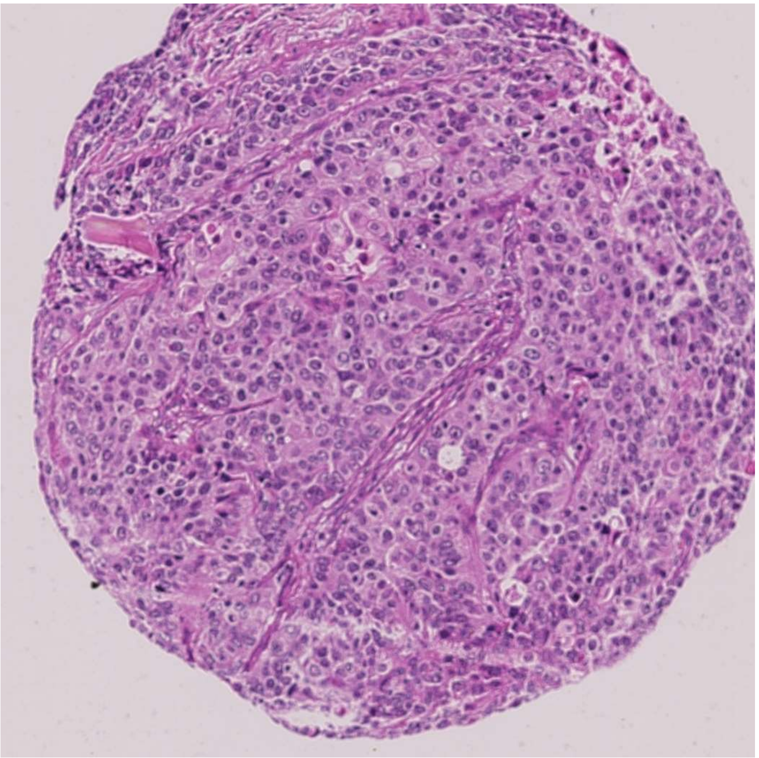

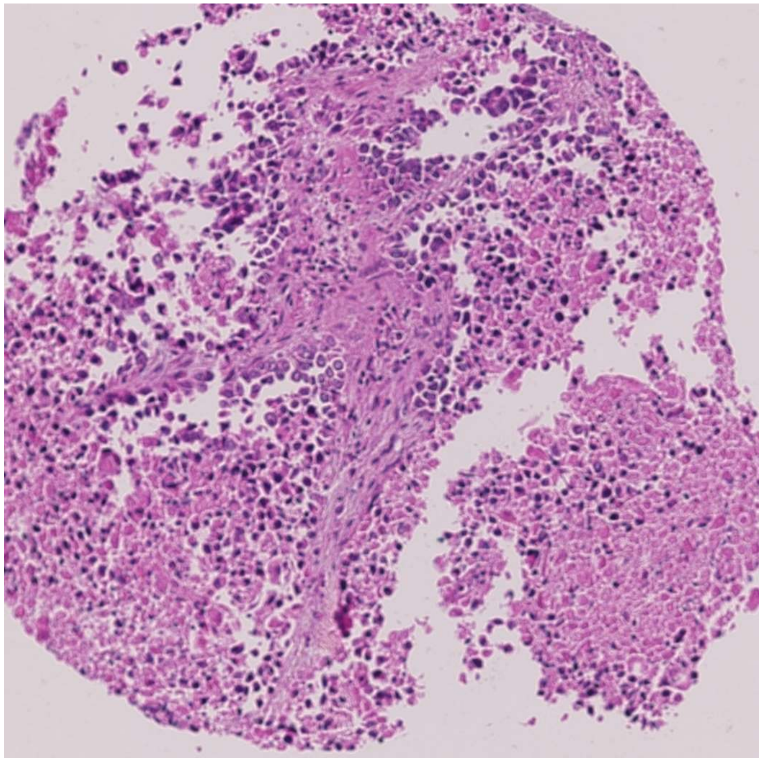

Low 1

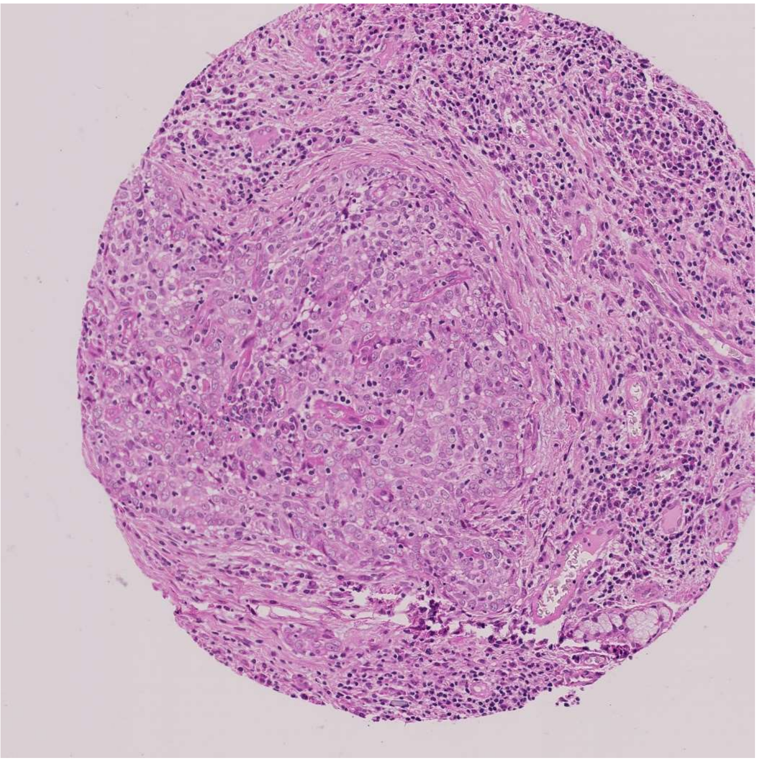

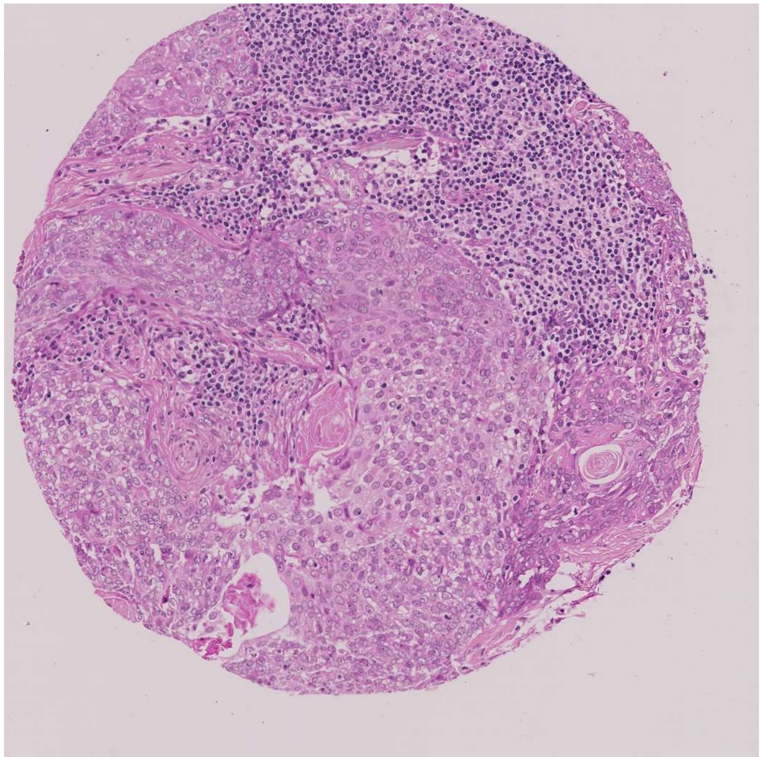

Low 2

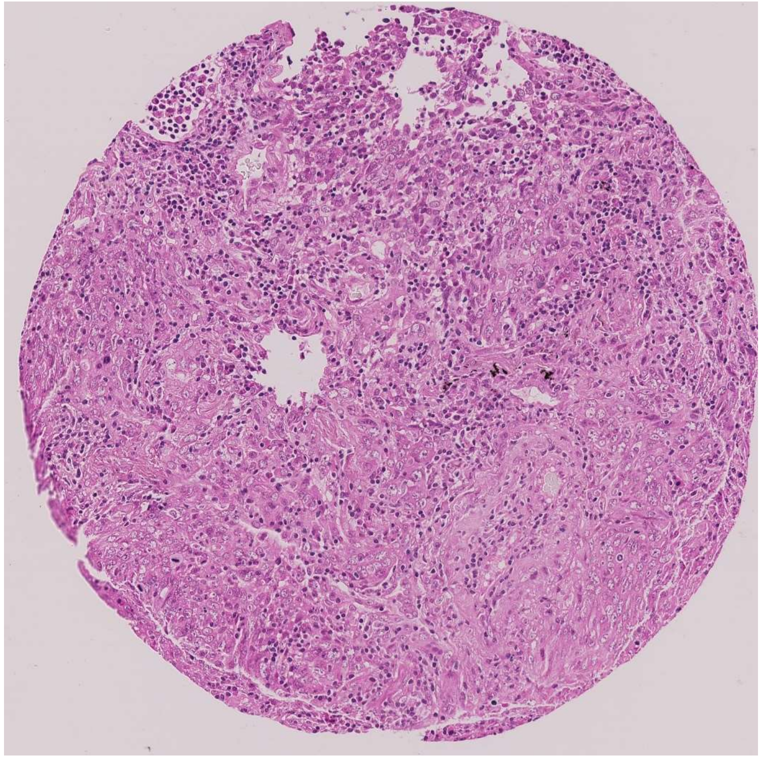

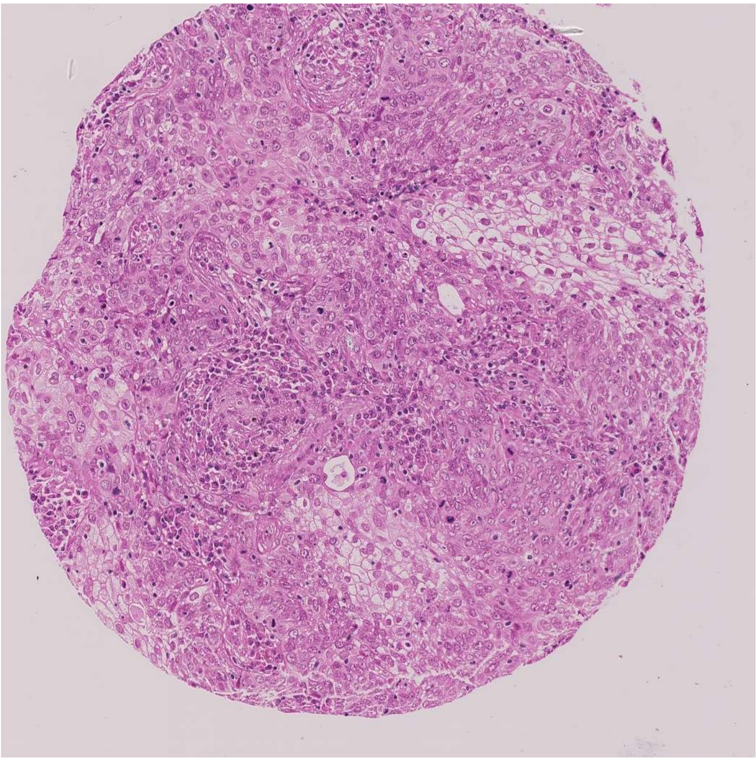

Low 3

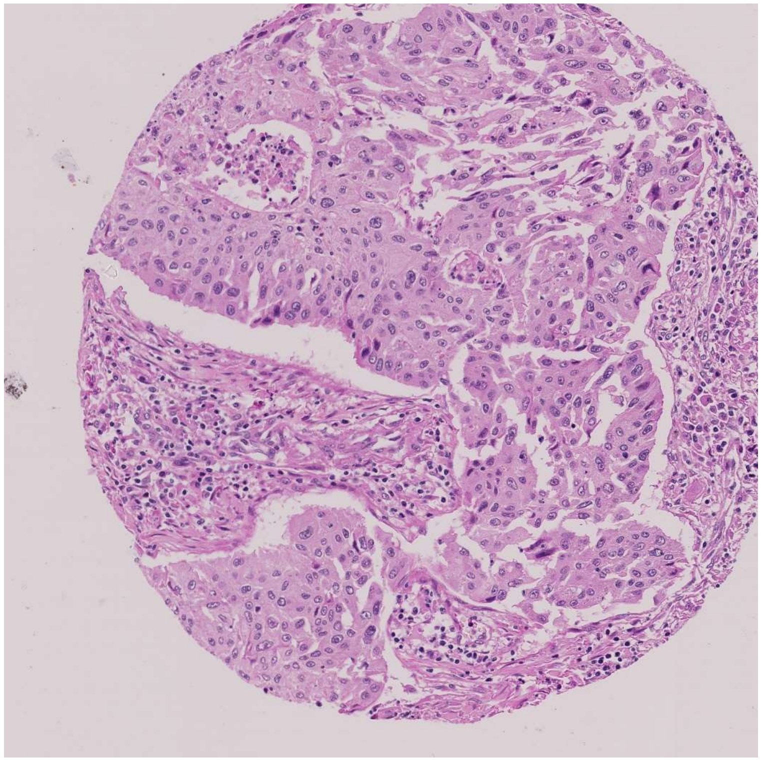

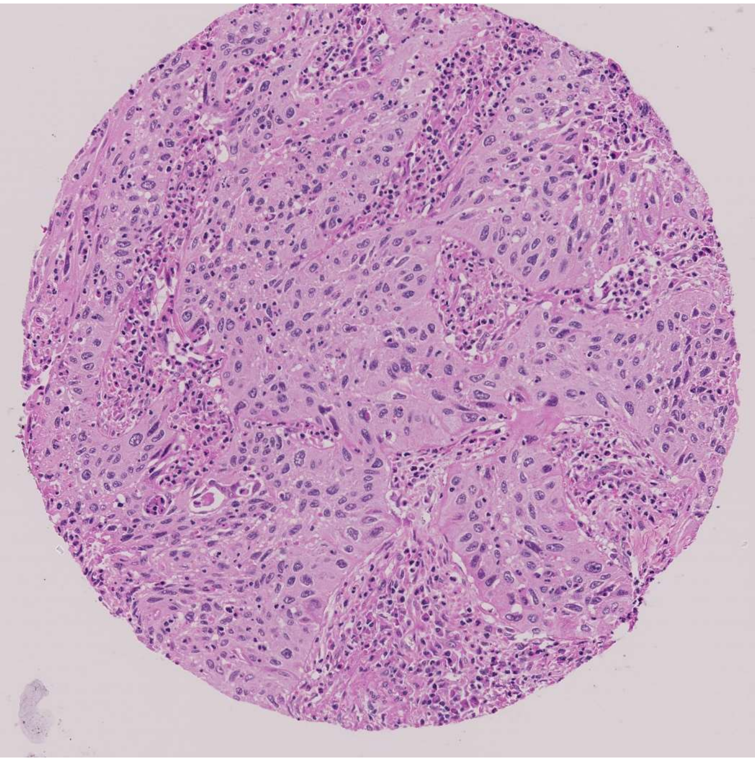

Low 4

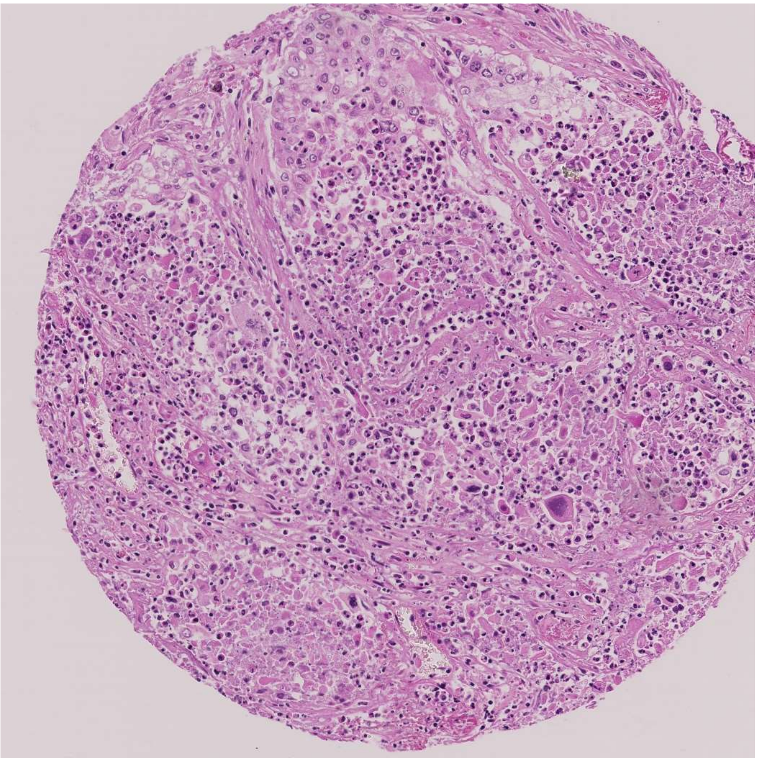

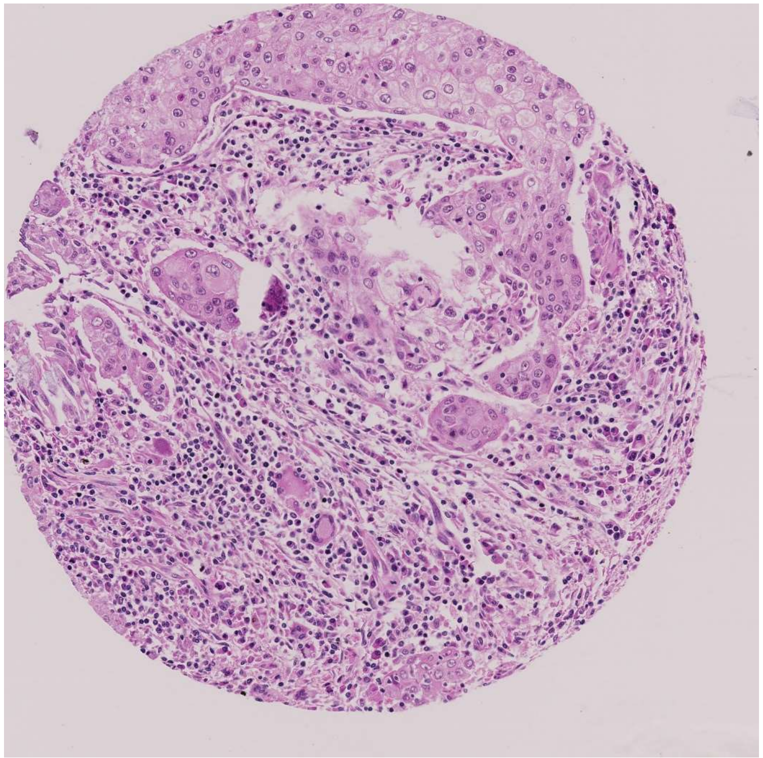

Low 5

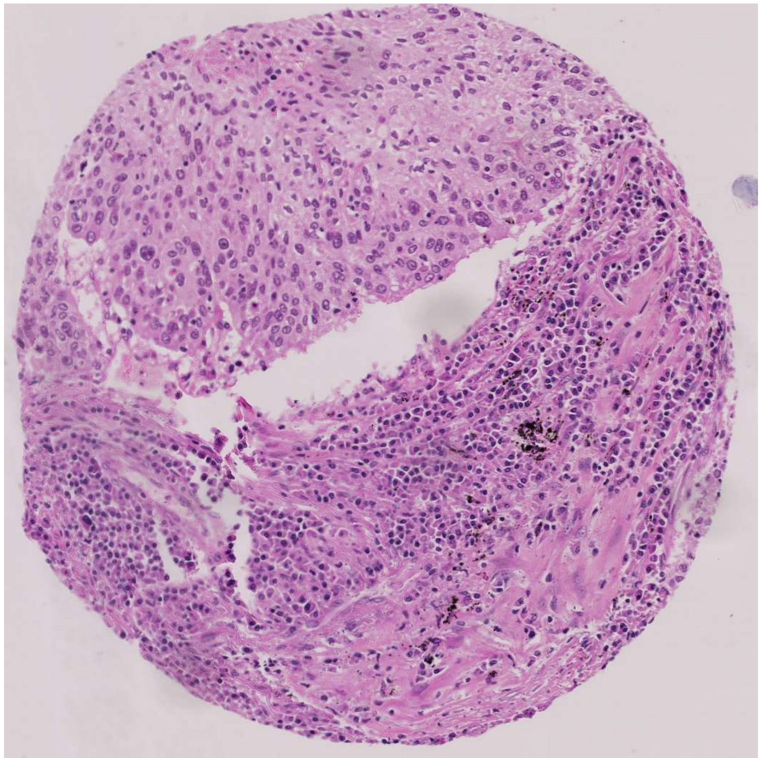

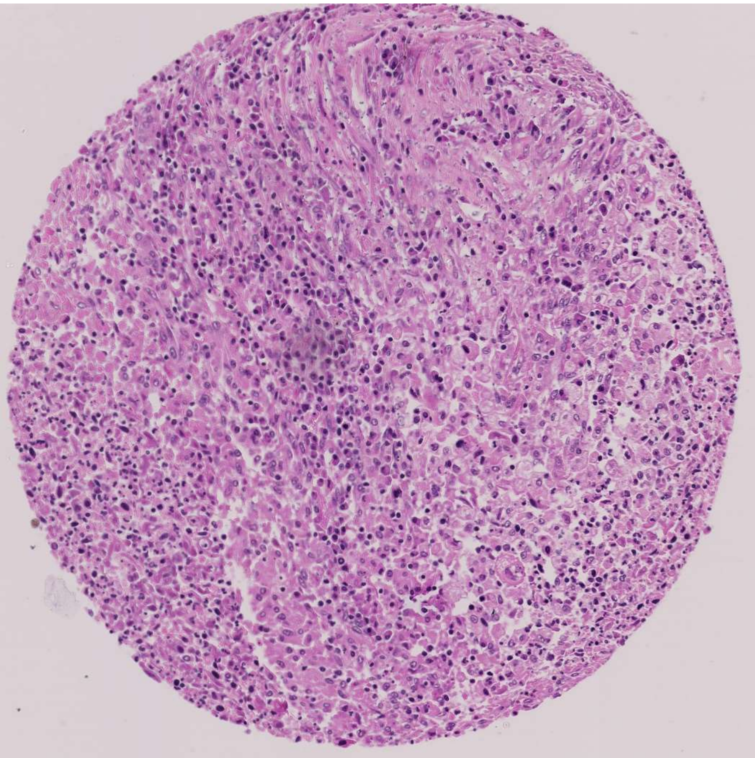

Low 6

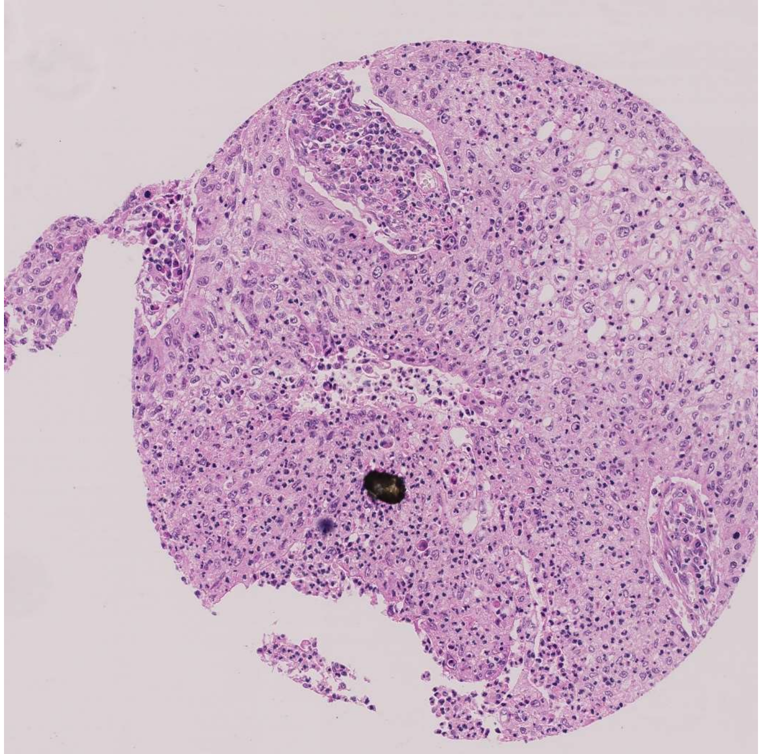

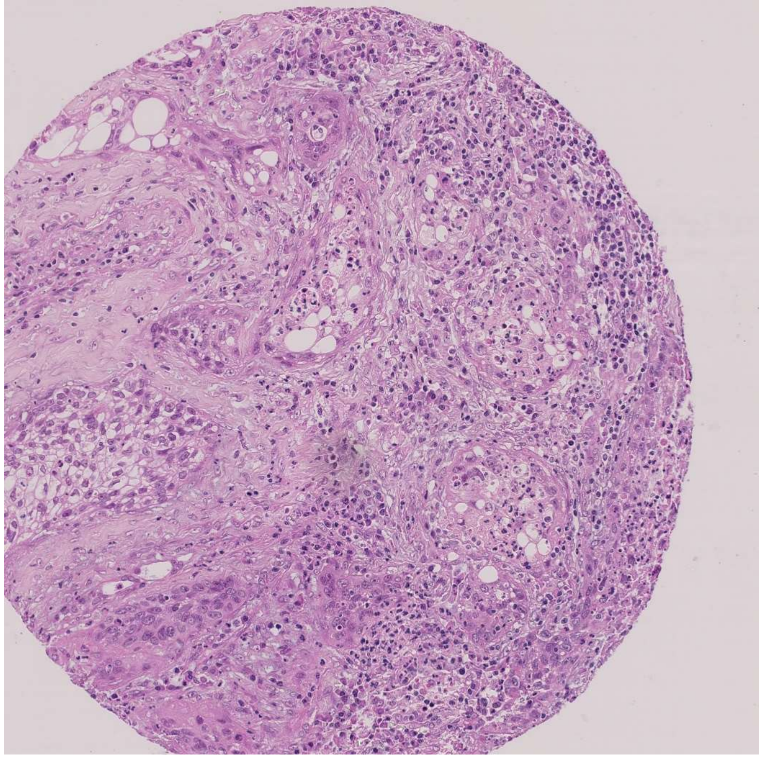

Low 7

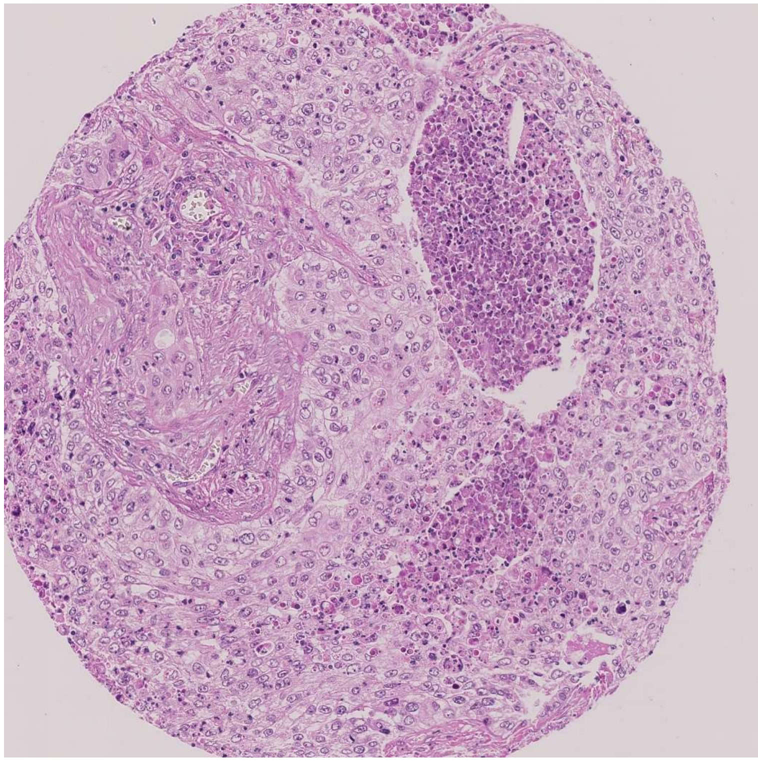

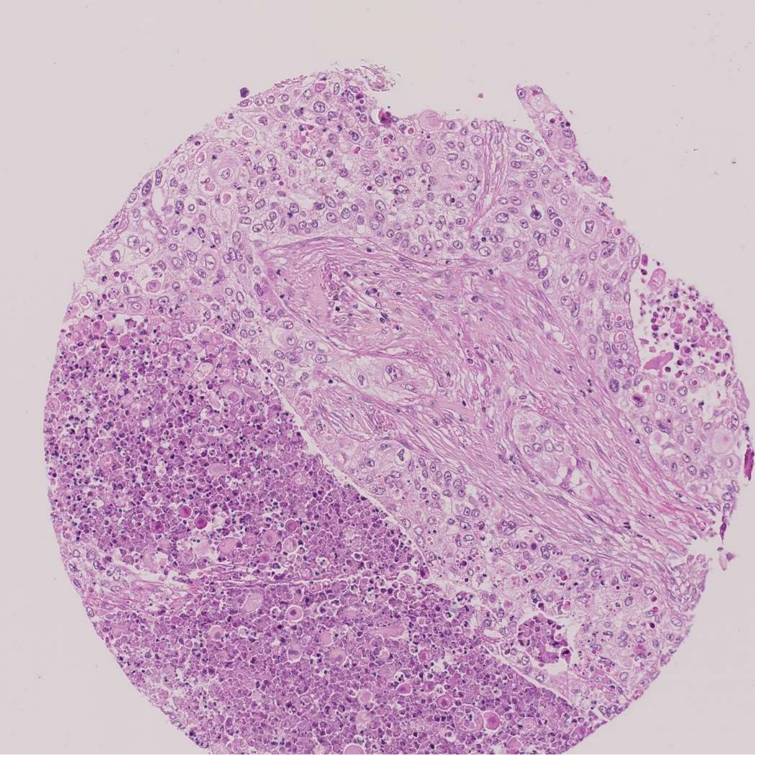

Low 8

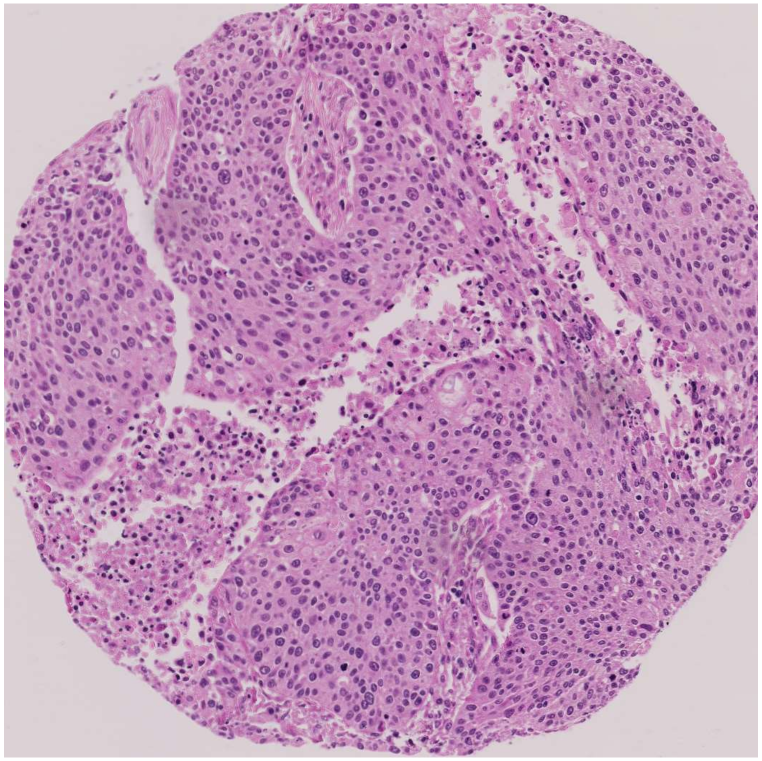

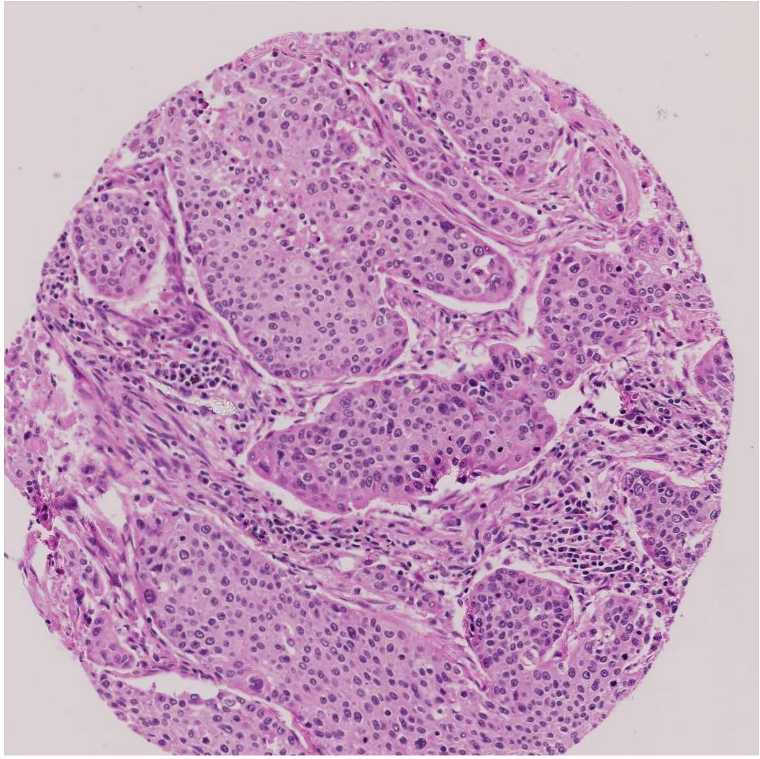

Low 9

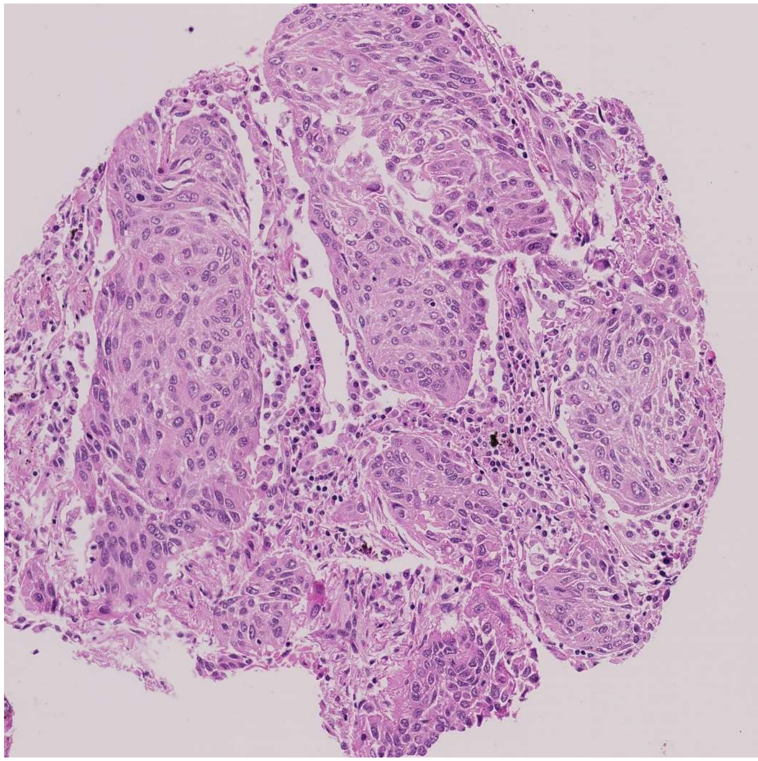

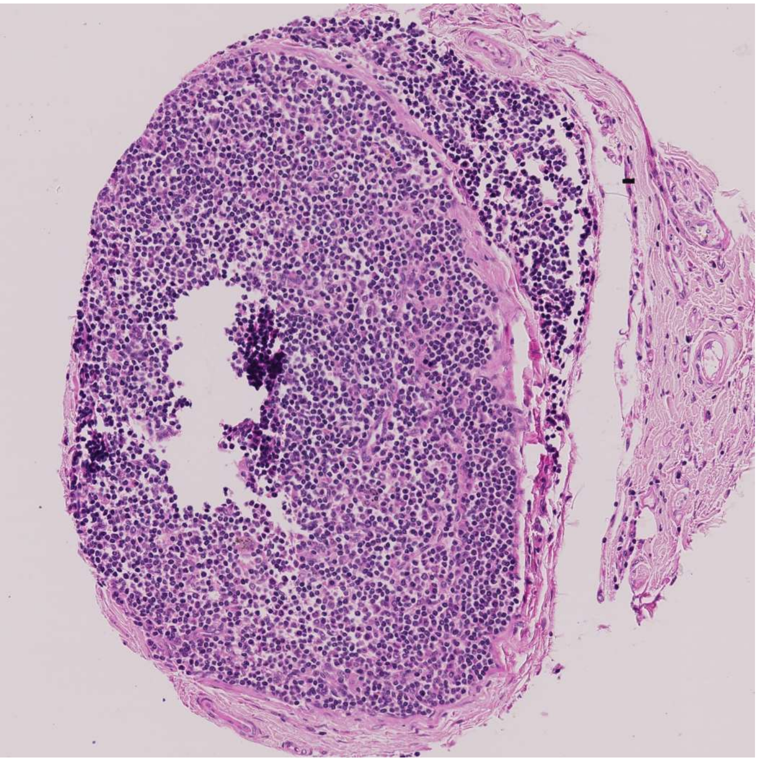

Low 10

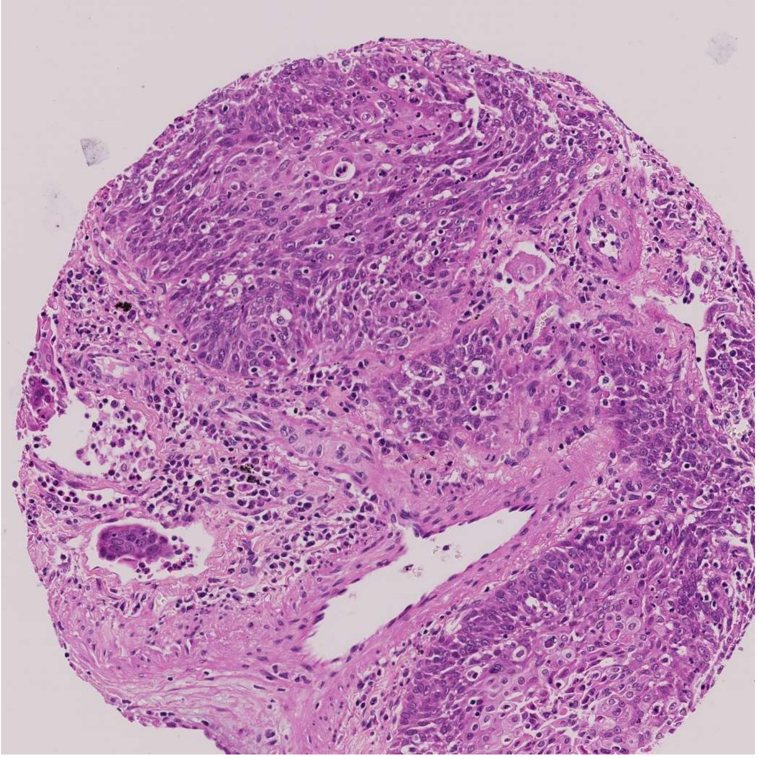

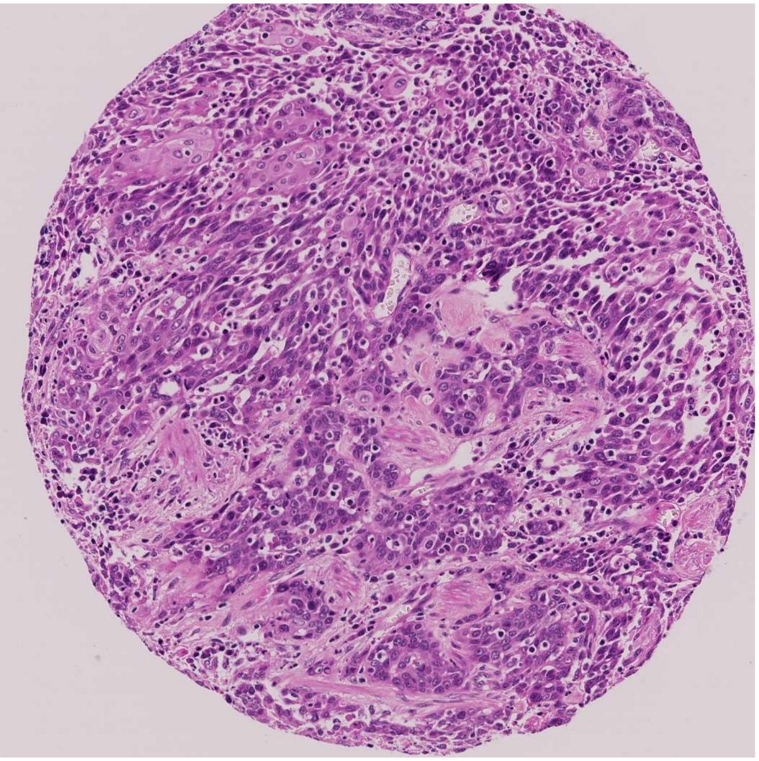

Summary of this feature

- **Density** is a measure of number of pixels in an object divided by the approximate radius of the object; a square is the most dense, an elongated object is less dense
- **Stroma Nucleus Standard Deviation Density** measures the uniformity of the density feature across all nuclei in the stroma of an image
- As one might imagine…
  - A stroma rich in lymphocytes would be more uniform and have a **lower** Std Dev
  - A stroma rich in spindle cells cut at different angles relative to the plane of section would be less uniform and have a **higher** Std Dev
- Consistent with this, the highest 10 cases have less **stromal chronic inflammation** than lowest 10 cases by manual scoring (see next slide)

Inflammation

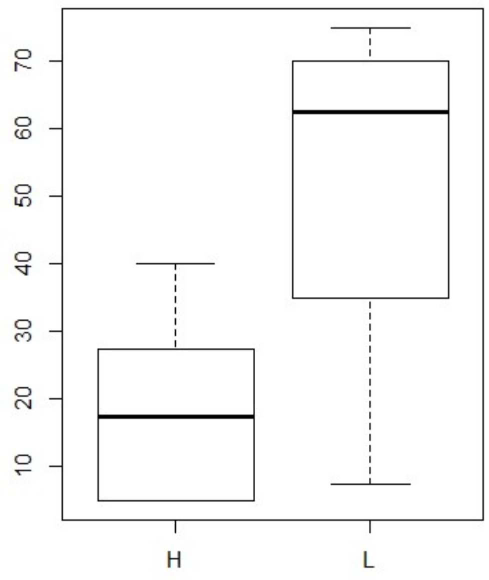

- H = highest 10
- L = lowest 10
- P = 0.000644

Survival

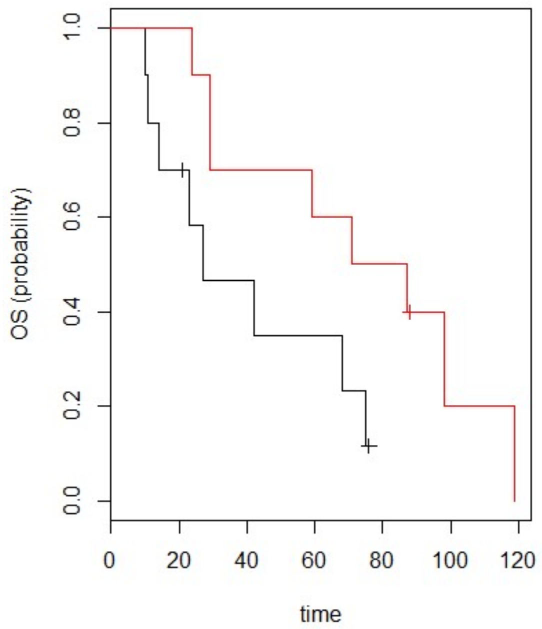

- Red = lowest 10
- Black = highest 10
- p = 0.0471

Stroma.Nucleus.Mean.Length.Width

What is Stroma.Nucleus.Mean.Length.Width?

The length-to-width ratio of an image object. There are two methods to approximate this:

1. The ratio of length to width is identical to the ratio of the eigenvalues of the covariance matrix, with the larger eigenvalue being the numerator of the fraction:

$$\gamma_v^{EV} = \frac{\lambda_1(v)}{\lambda_2(v)}$$

05 September 2012 Reference Book

2. The ratio of length to width can also be approximated using the bounding box:

$$\gamma_v^{BB} = \frac{\left(k_v^{bb'}\right)^2}{\#P_v}$$

Both calculations are compared; the smaller of both results is returned as the feature value.

Parameters

- $\#P_v$ is the Size of a set of pixels of an image object v
- $\lambda_1 \lambda_2$ are eigenvalues
- γ_v^{EV} is the ratio length of v of the eigenvalues
- γ_v^{BB} is the ratio length of v of the bounding box
- γ_v is the length-width ratio of an image object v
- $k_v^{\mathrm{BB}'}$
- $h_v^{\mathrm{BB}'}$
- a is the bounding box fill rate
- $\#Pxl$
- h
- w is the image layer weight
- $k_v^{bb^1} = \sqrt{(k_v^{bb'})^2 + (1-a)(h_v^{bb'})^2}$
- $a = \frac{\#P_v}{k_v^{bb'} - h_v^{bb'}}$
- $k \cdot h = \#P_v \Rightarrow k = \frac{\#P_v}{w}, h = \frac{\#P_v}{k} \Rightarrow \frac{k}{w} = \frac{k^2}{\#P_{xl}} = \frac{\#P_{xl}}{w^2}$

Expression

$\gamma_v = \min \gamma_v^{EV}, \max \gamma_v^{BB}$

Feature Value Range

$[0, \infty]$

High 1

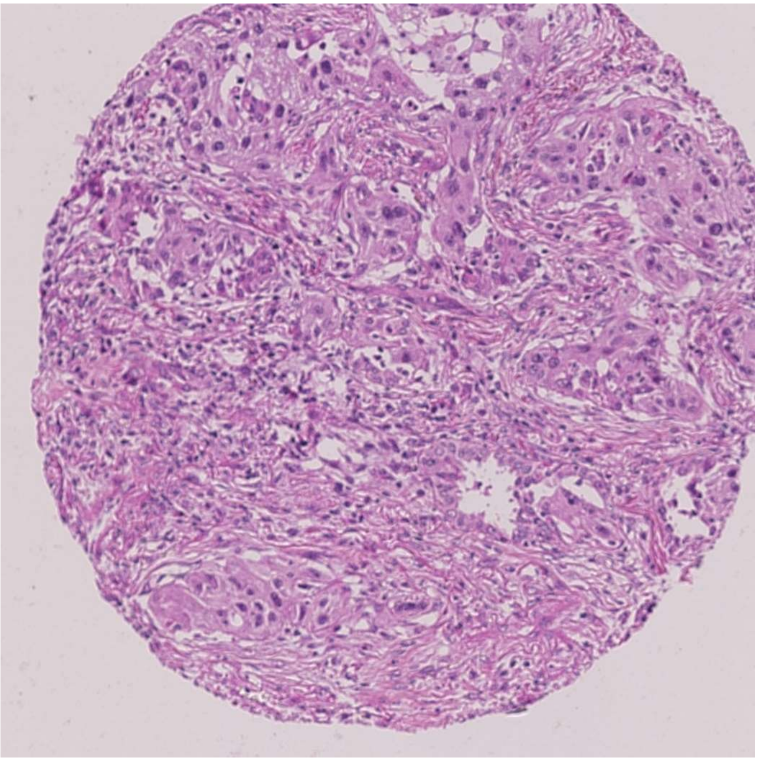

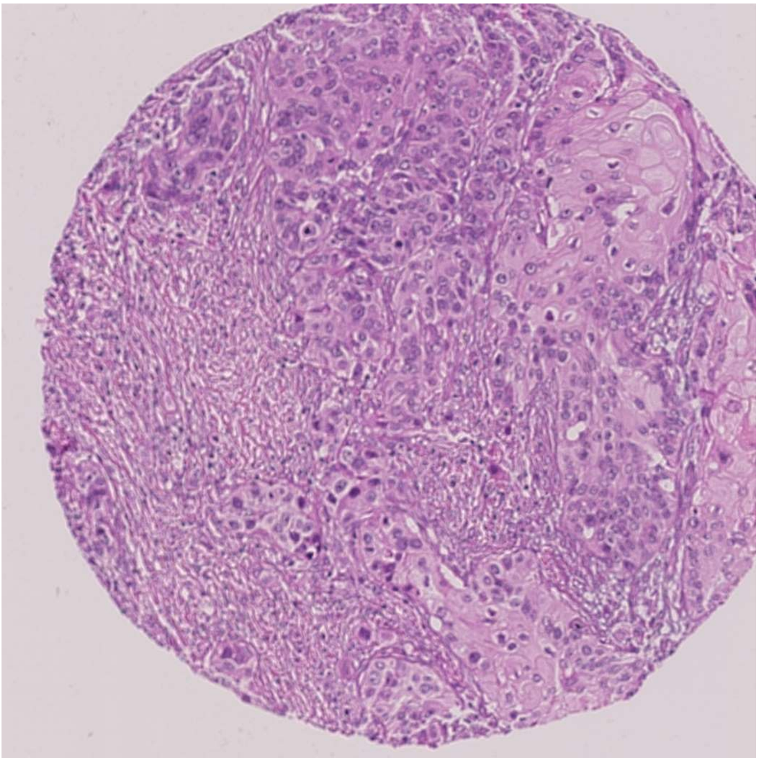

High 2

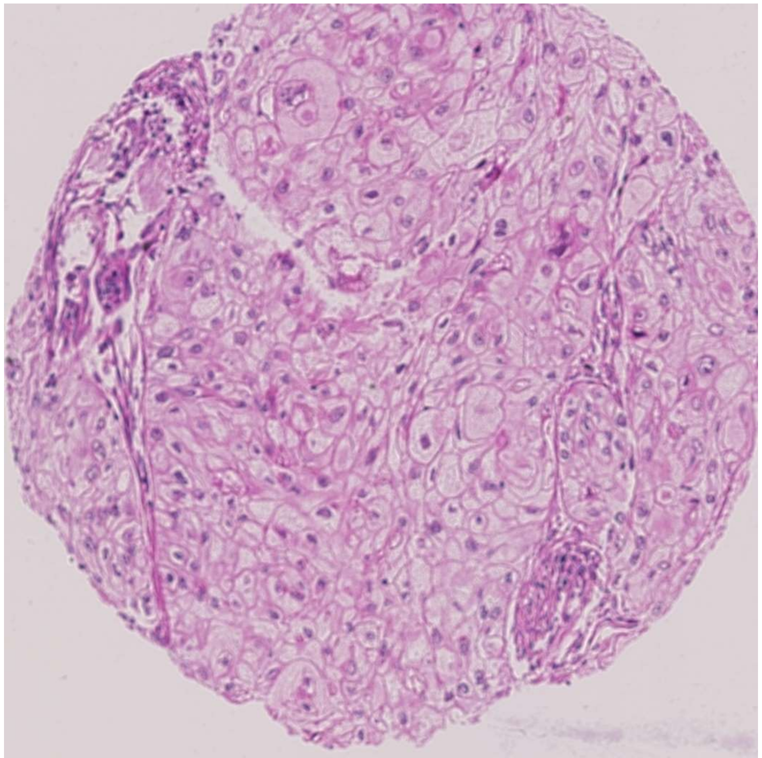

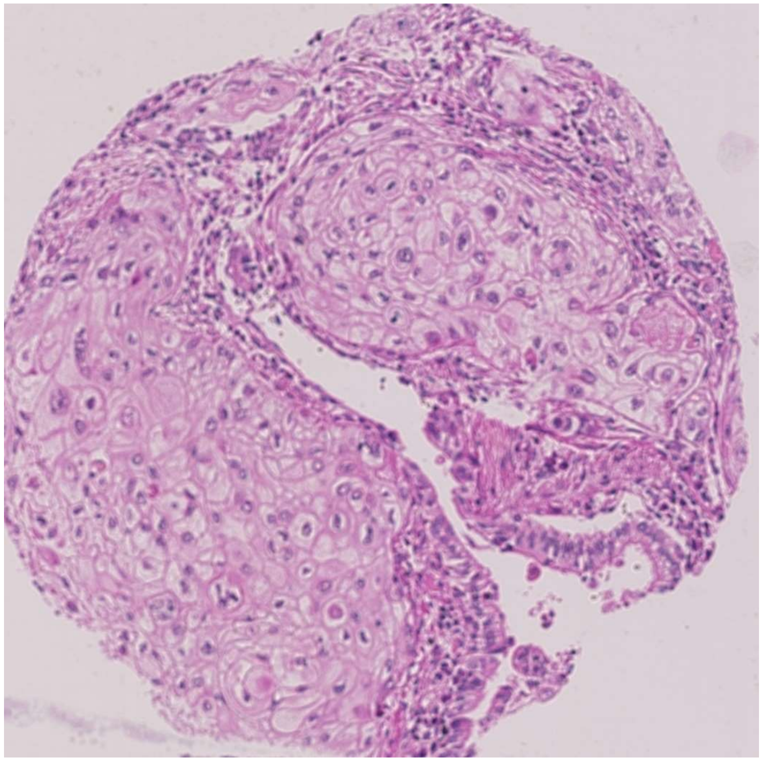

High 3

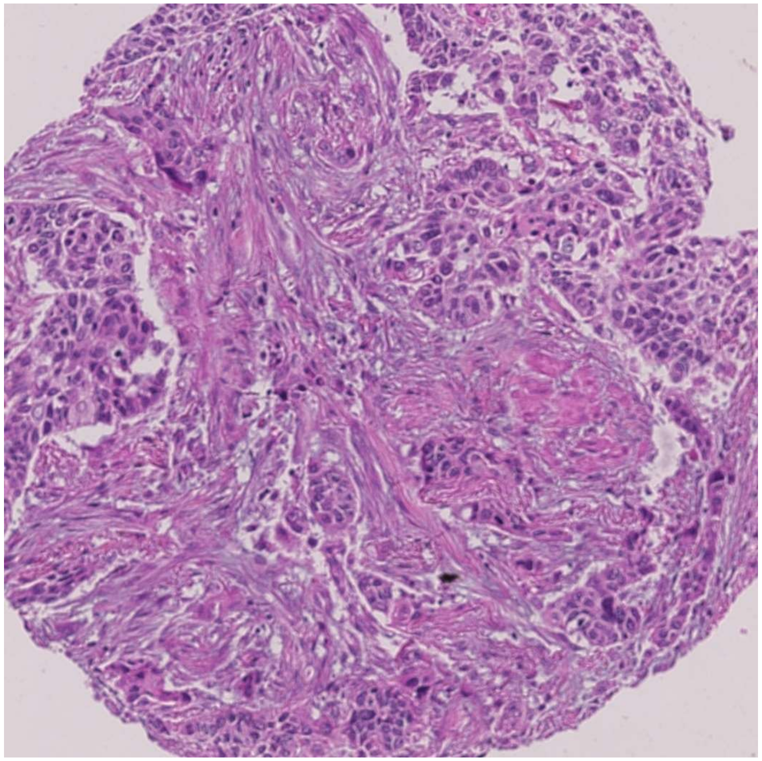

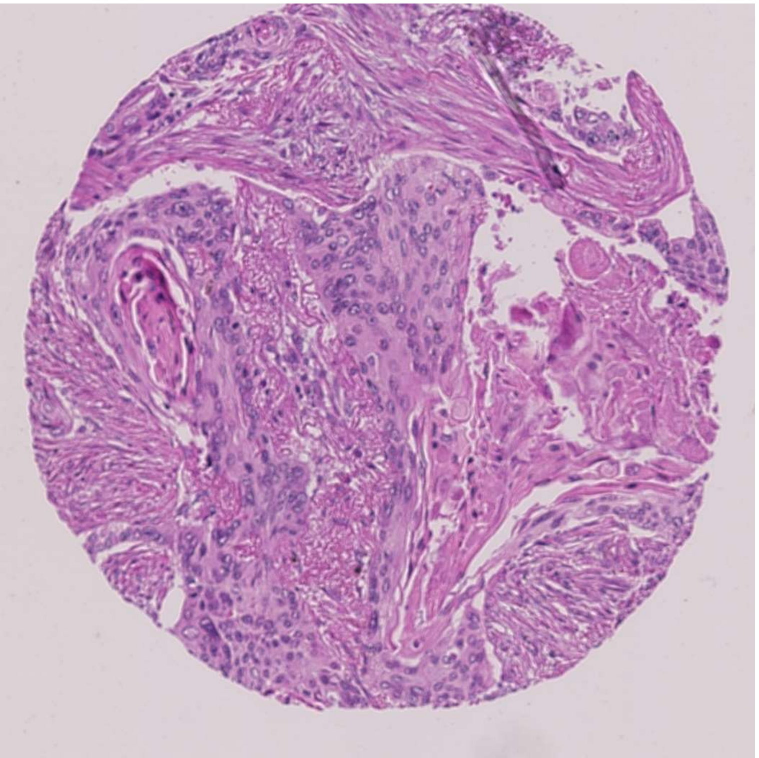

High 4

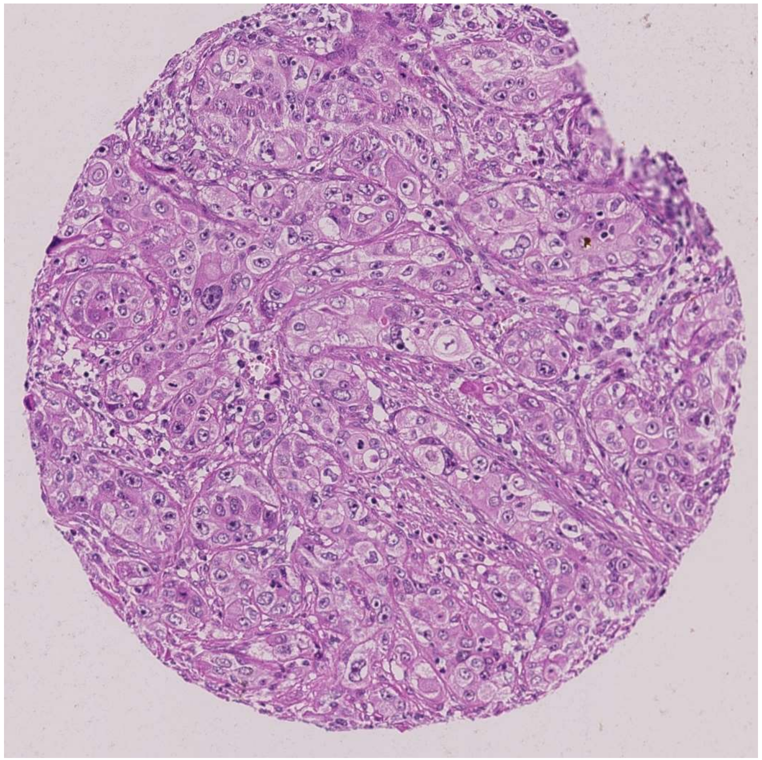

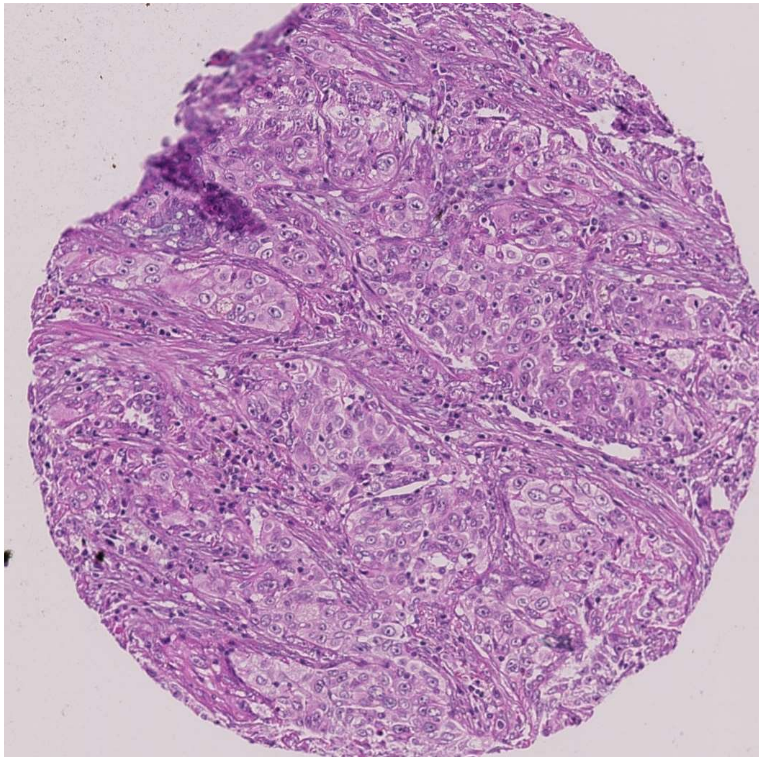

High 5

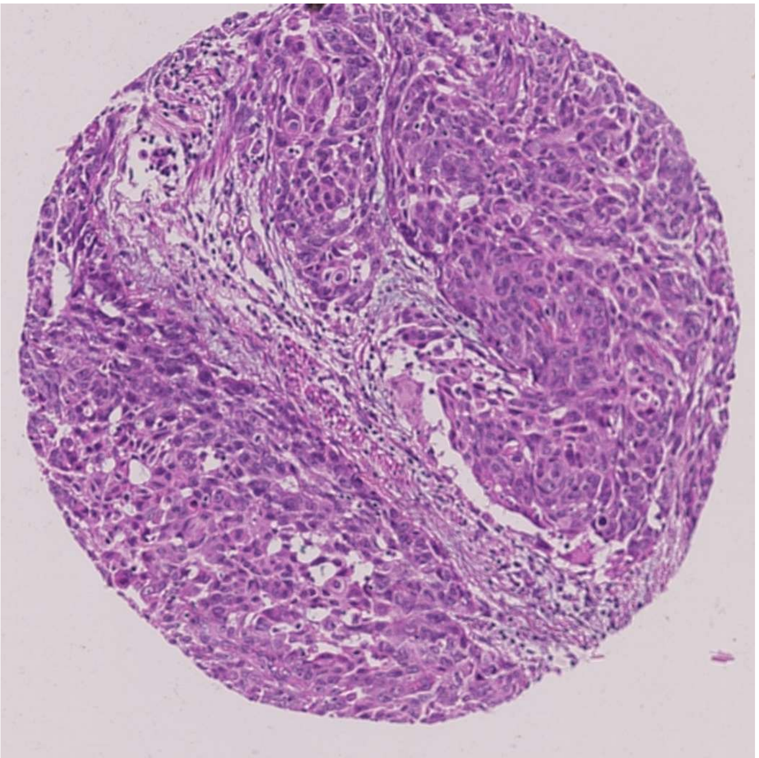

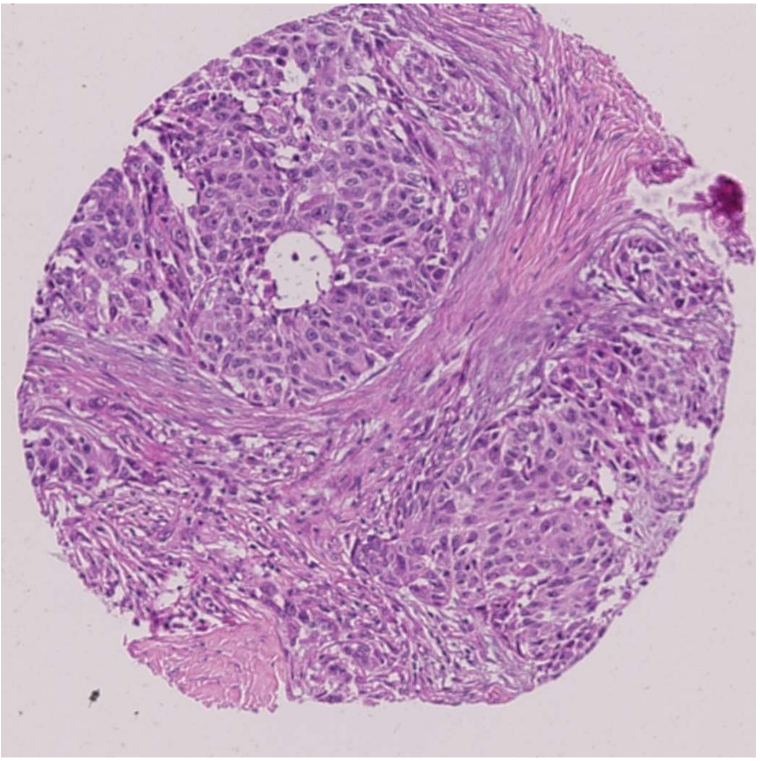

High 6

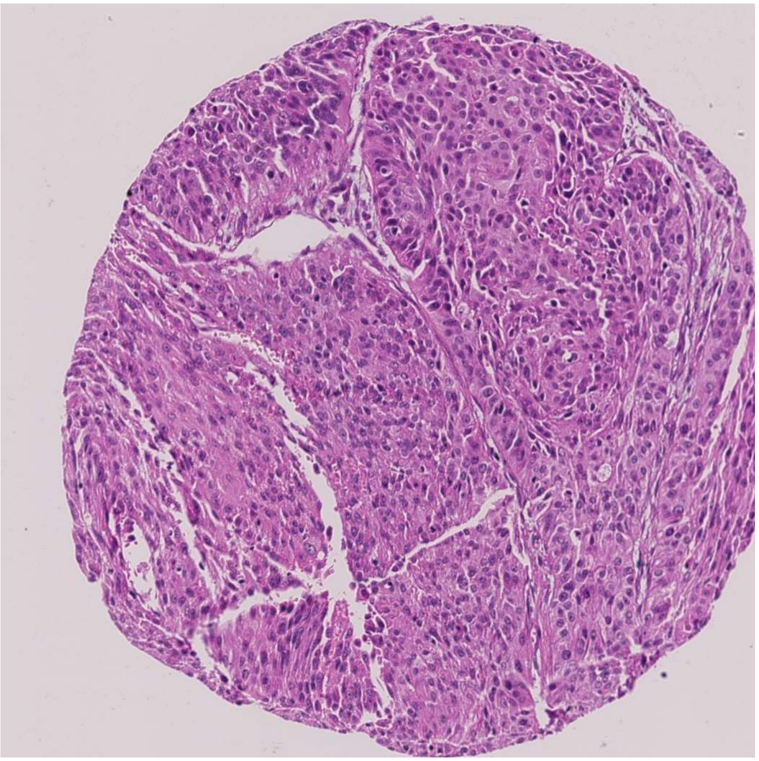

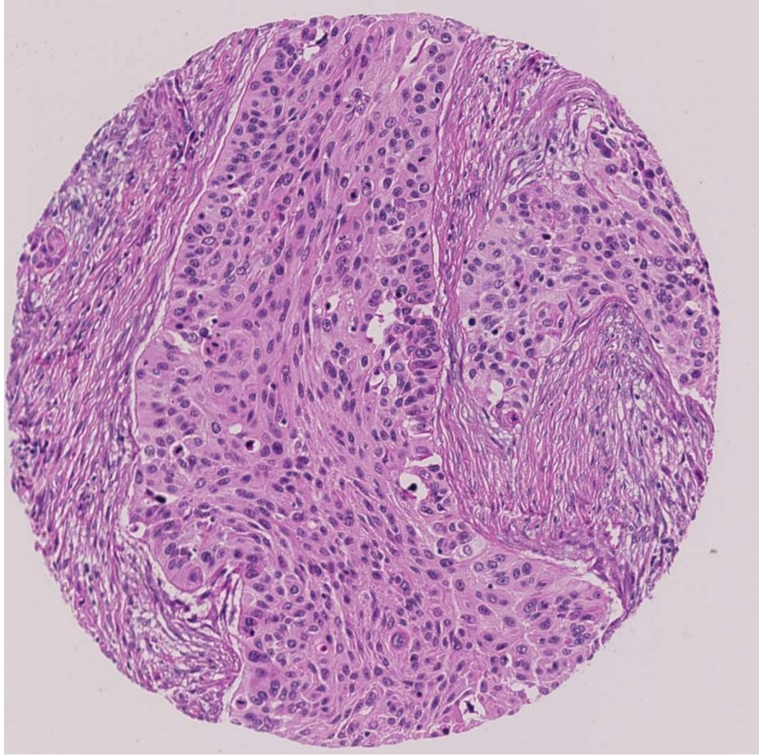

High 7

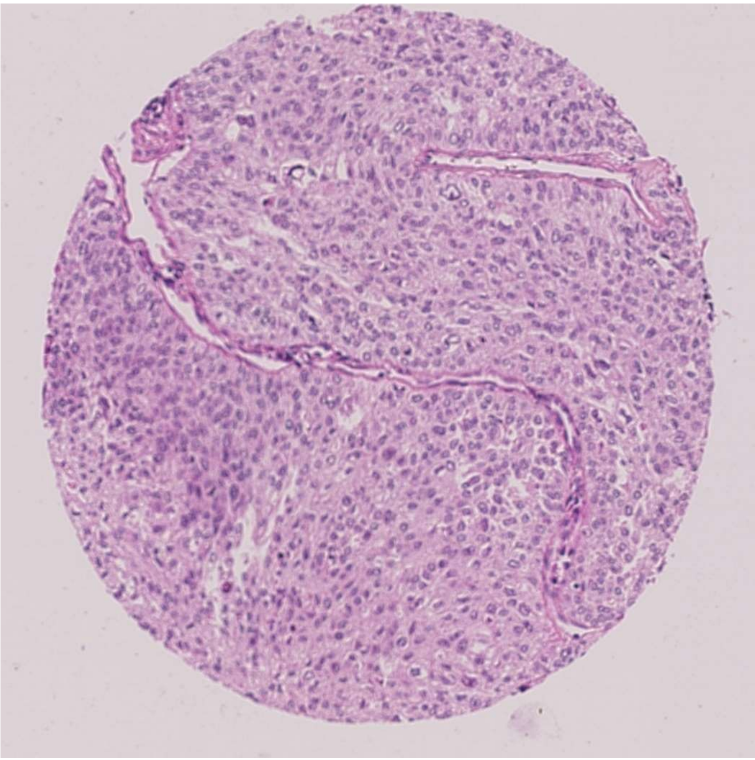

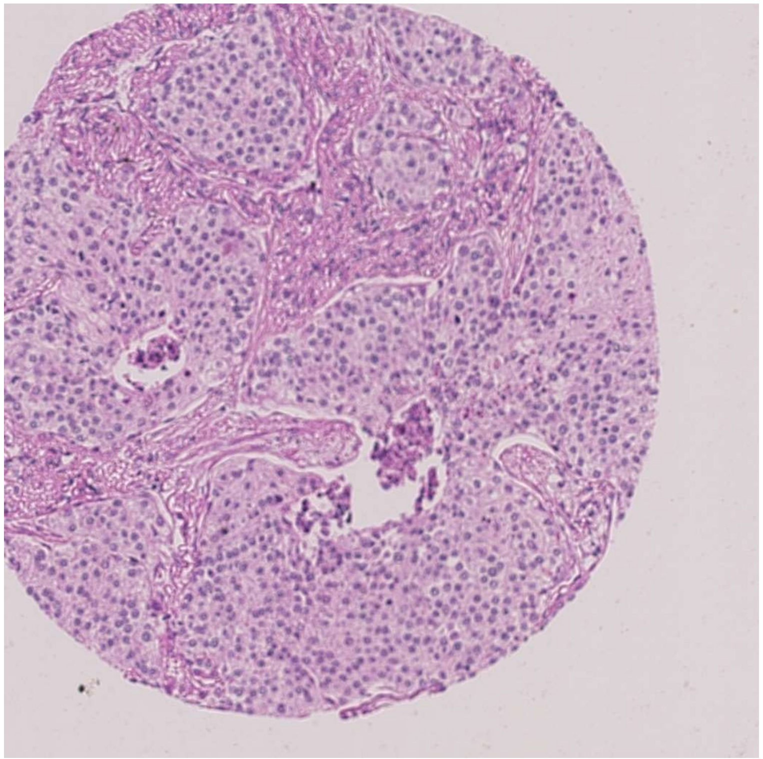

High 8

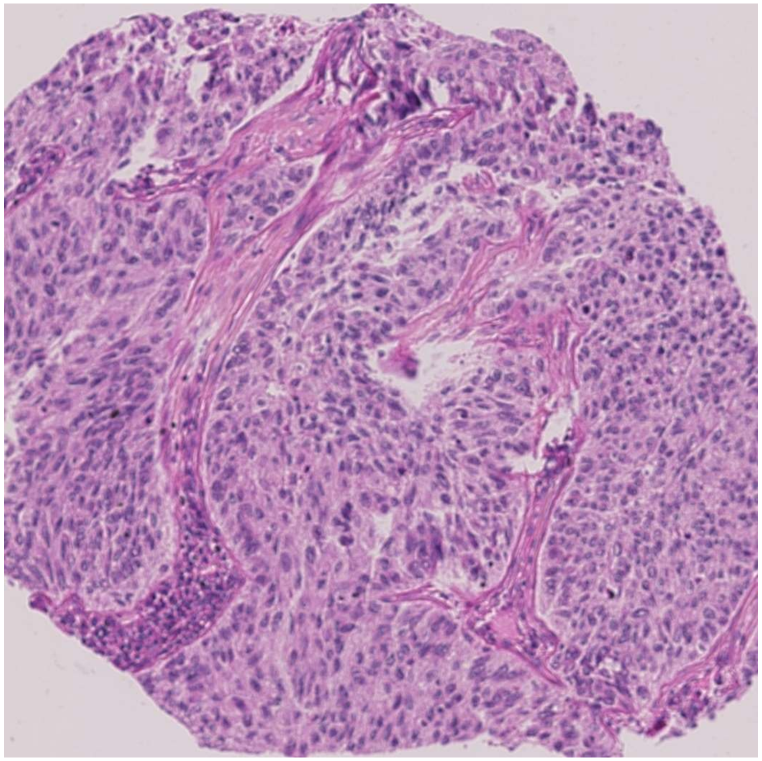

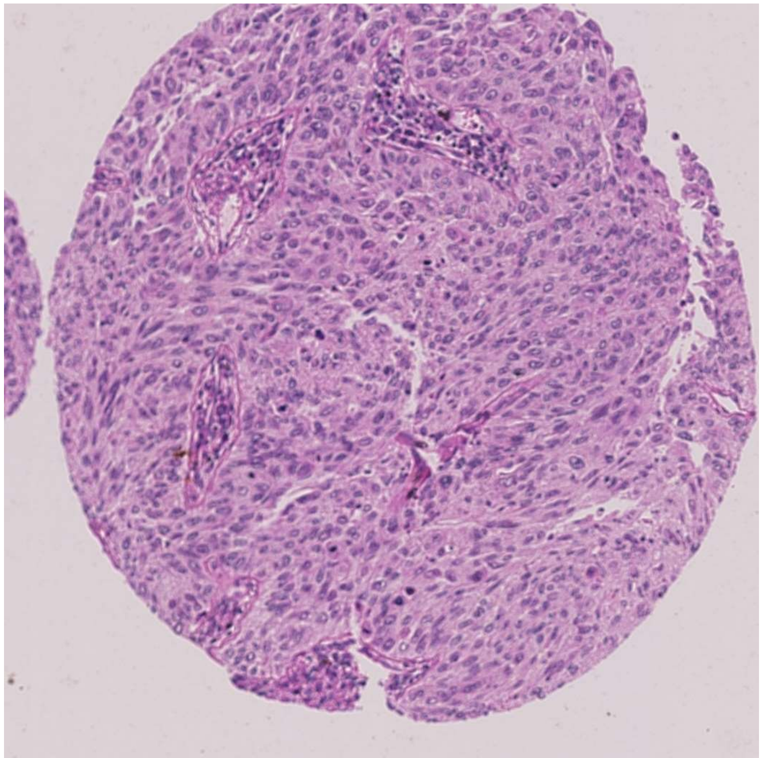

High 9

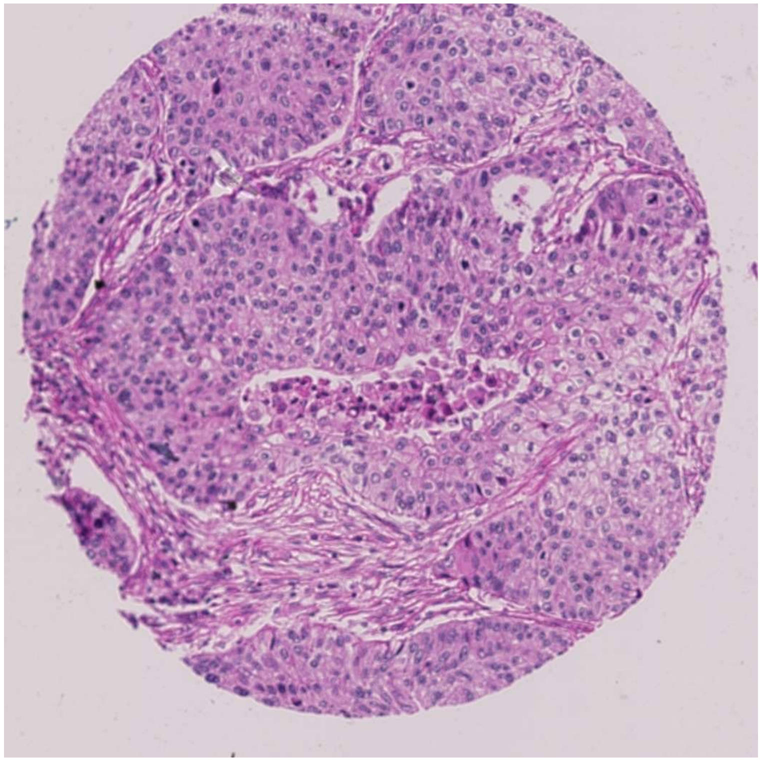

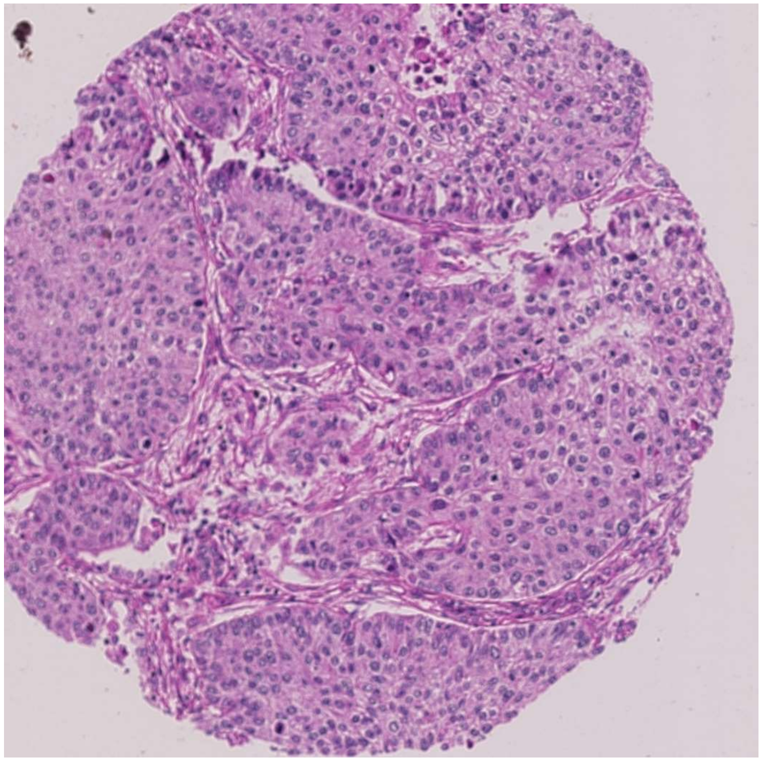

High 10

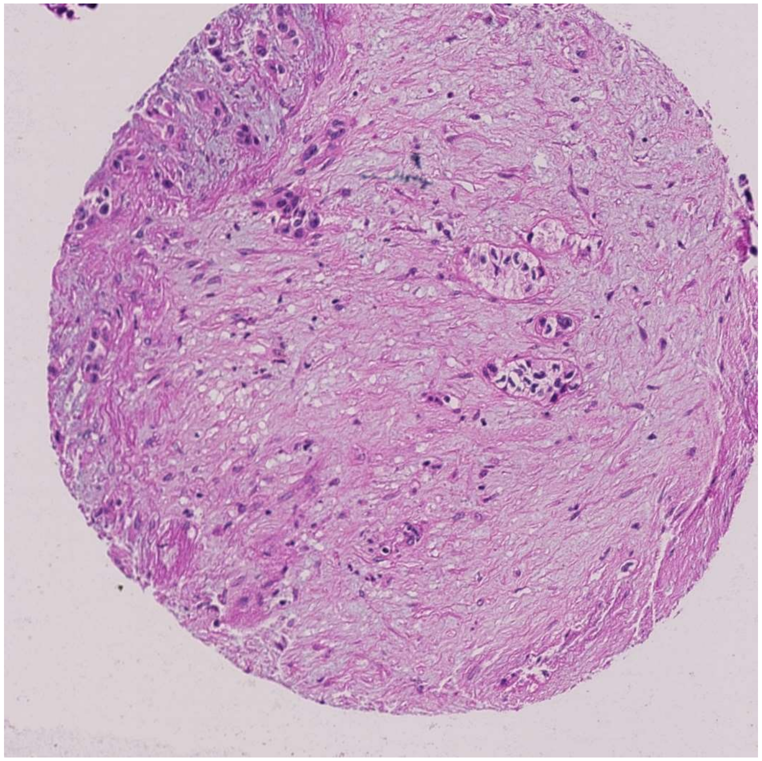

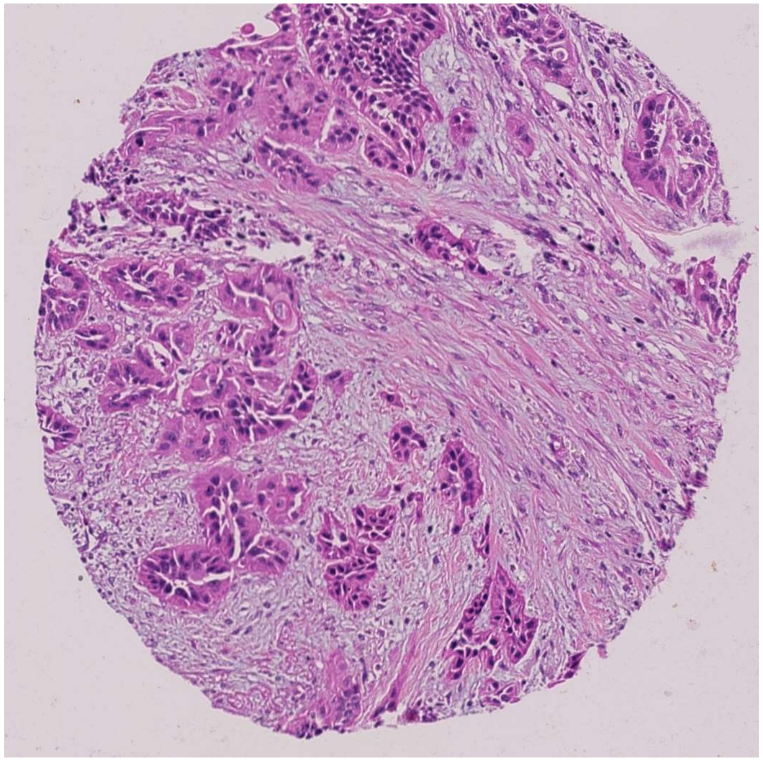

Low 1

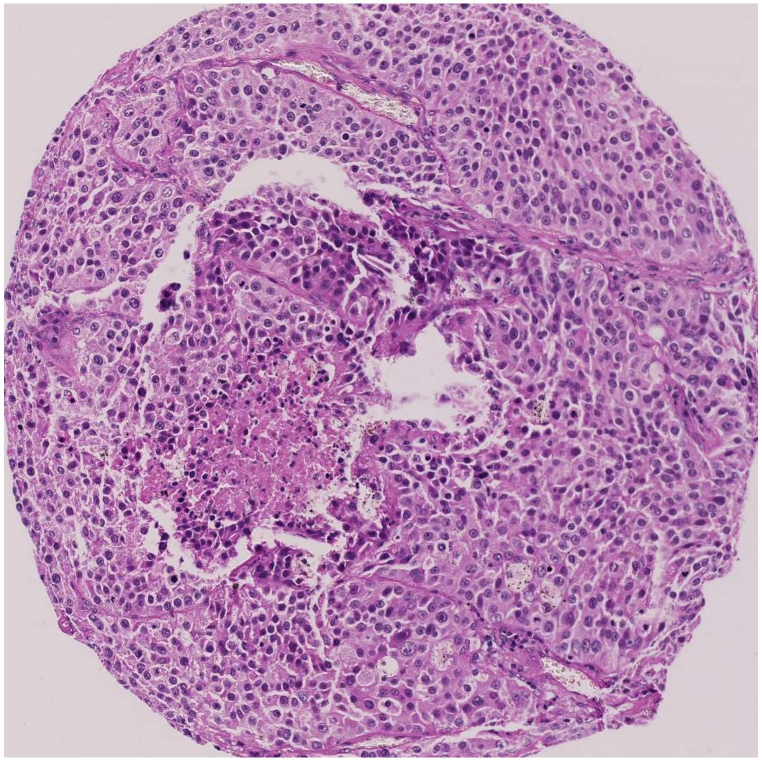

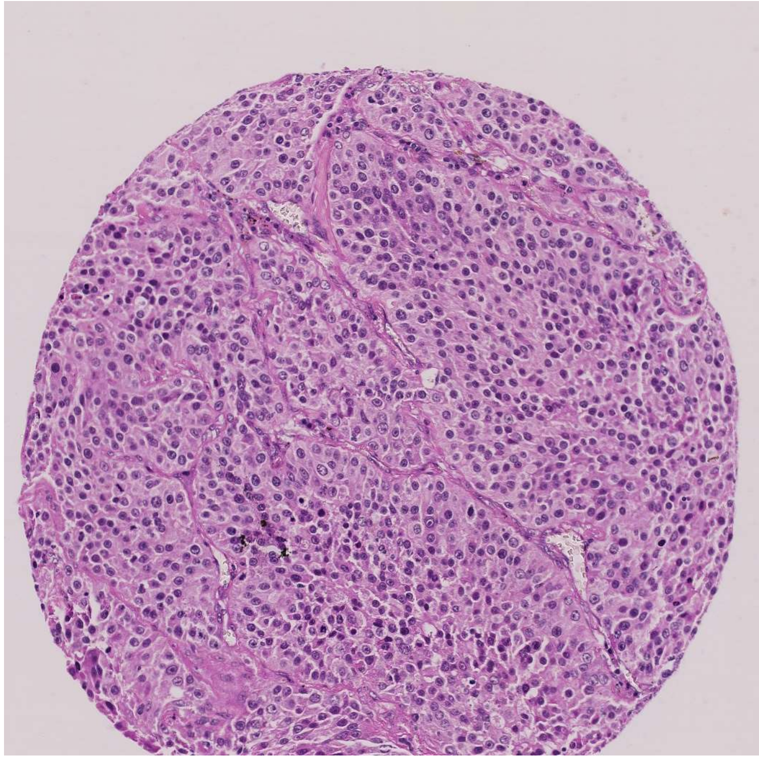

Low 2

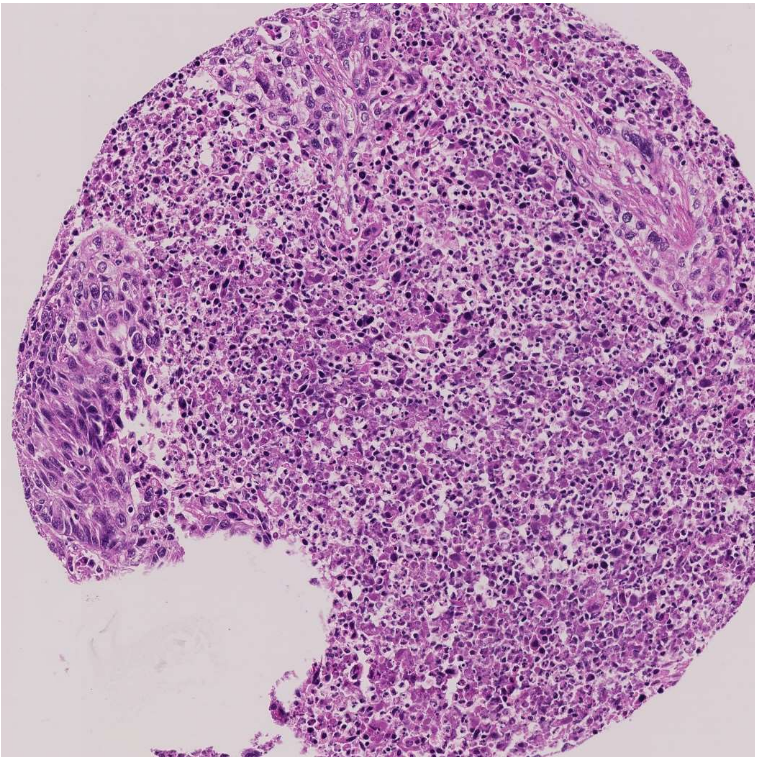

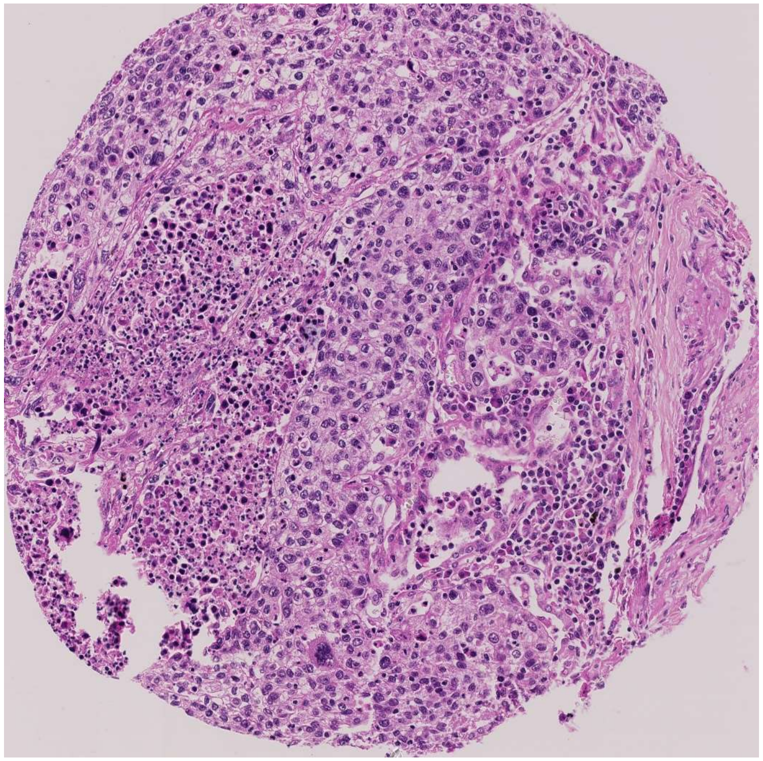

Low 3

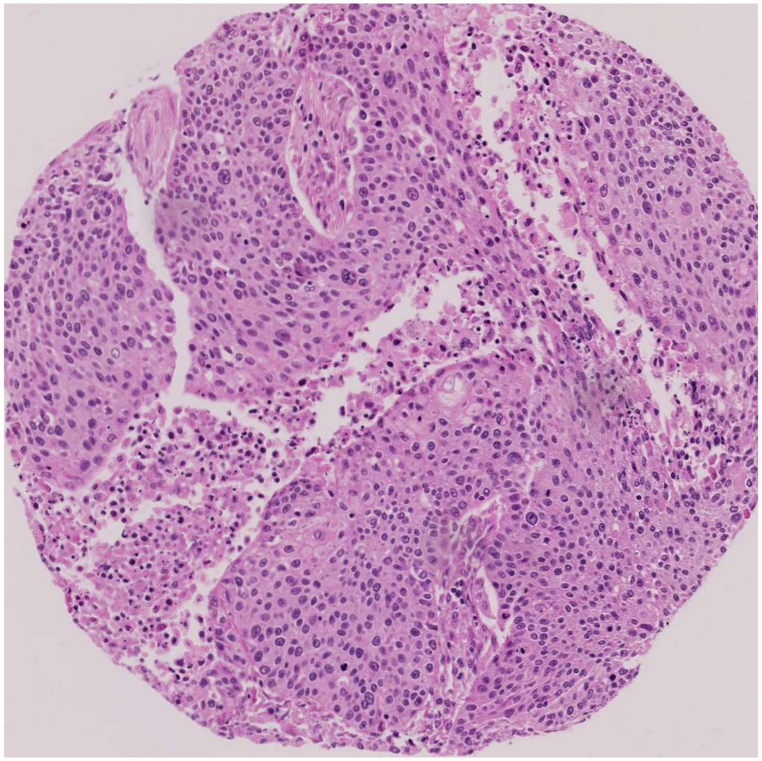

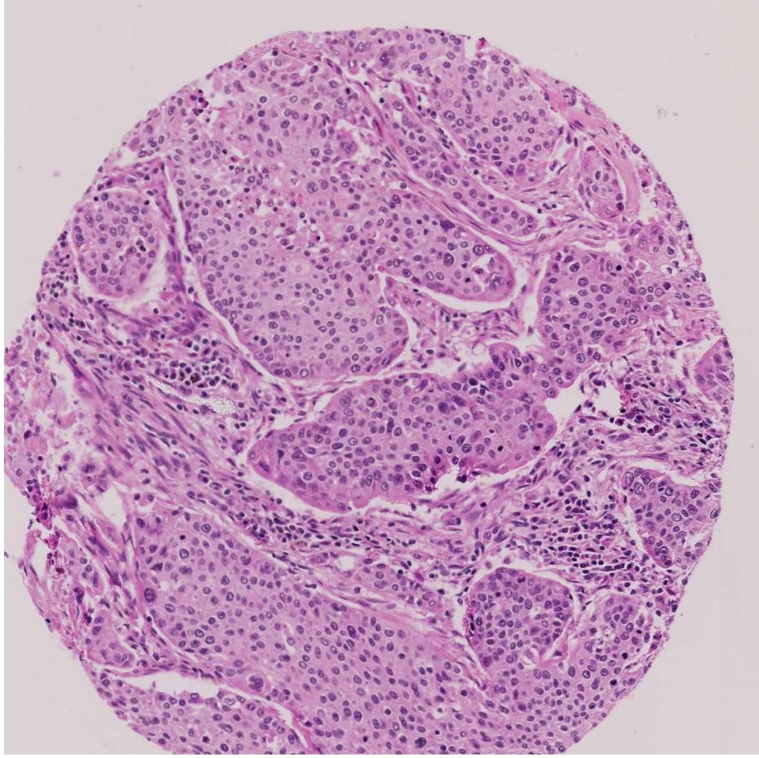

Low 4

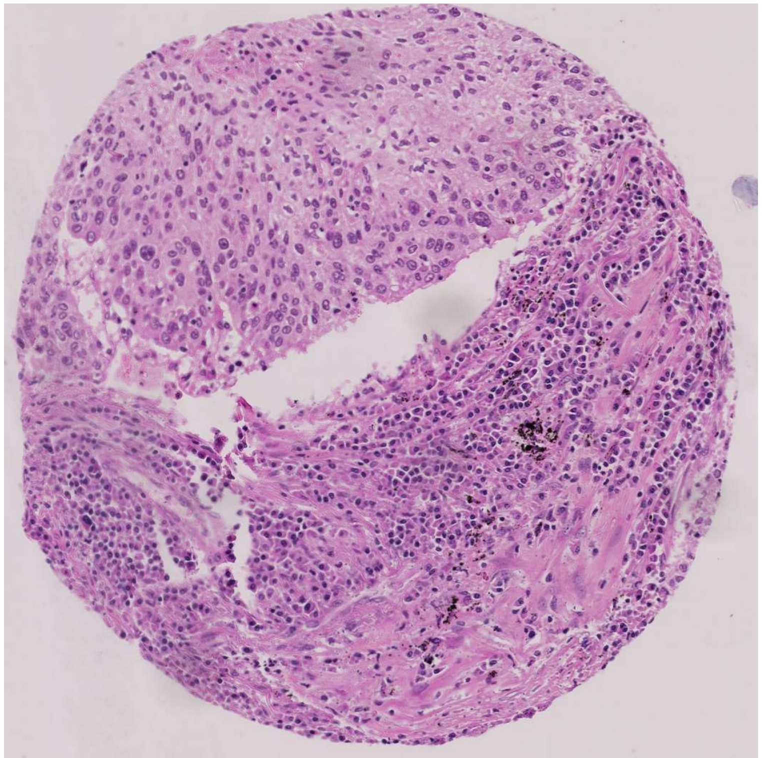

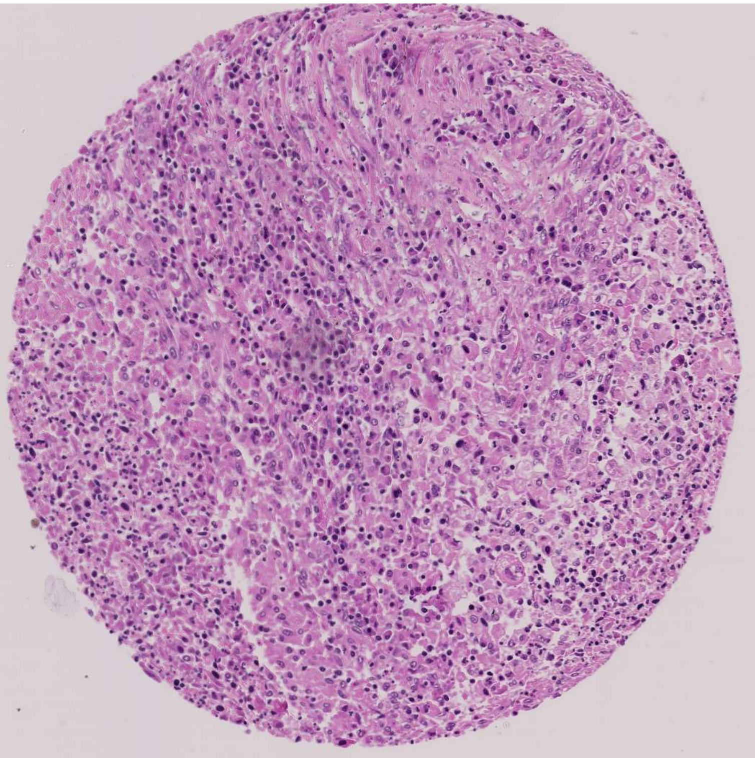

Low 5

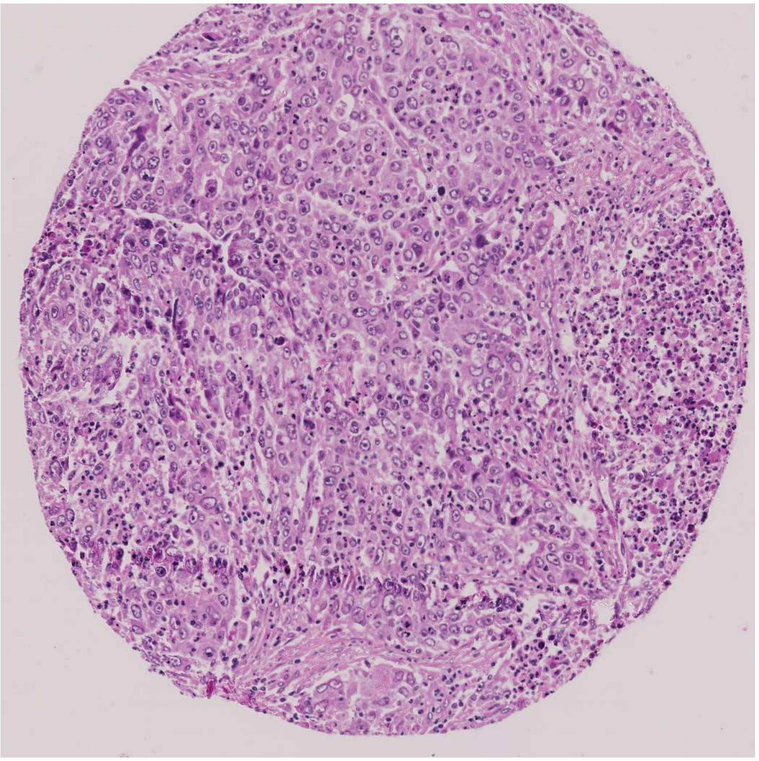

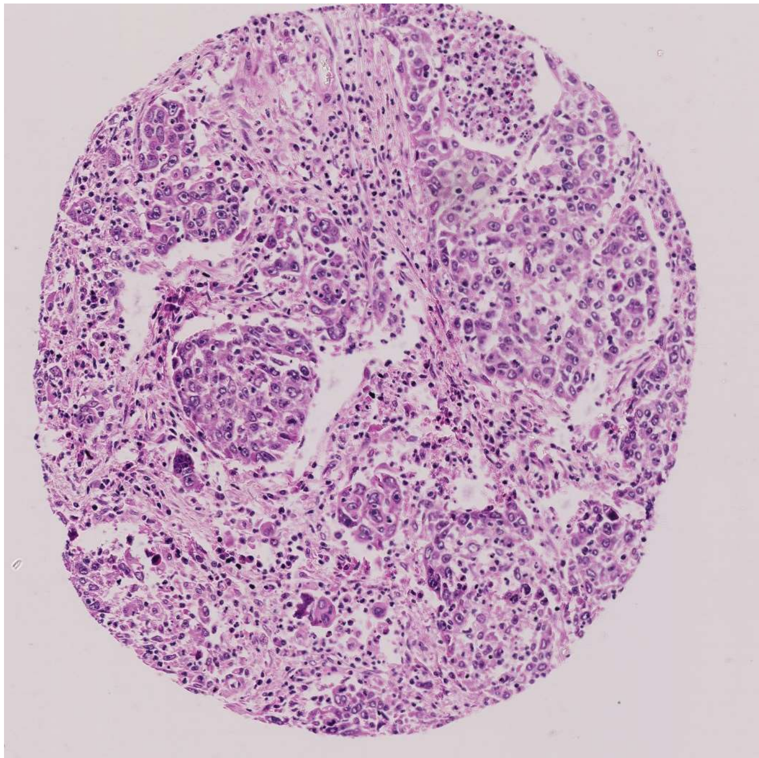

Low 6

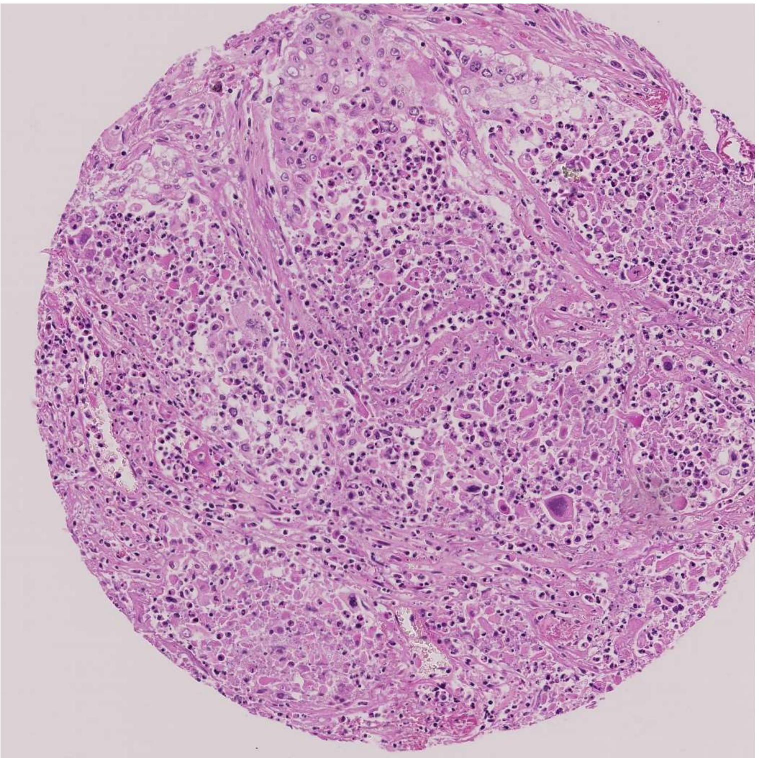

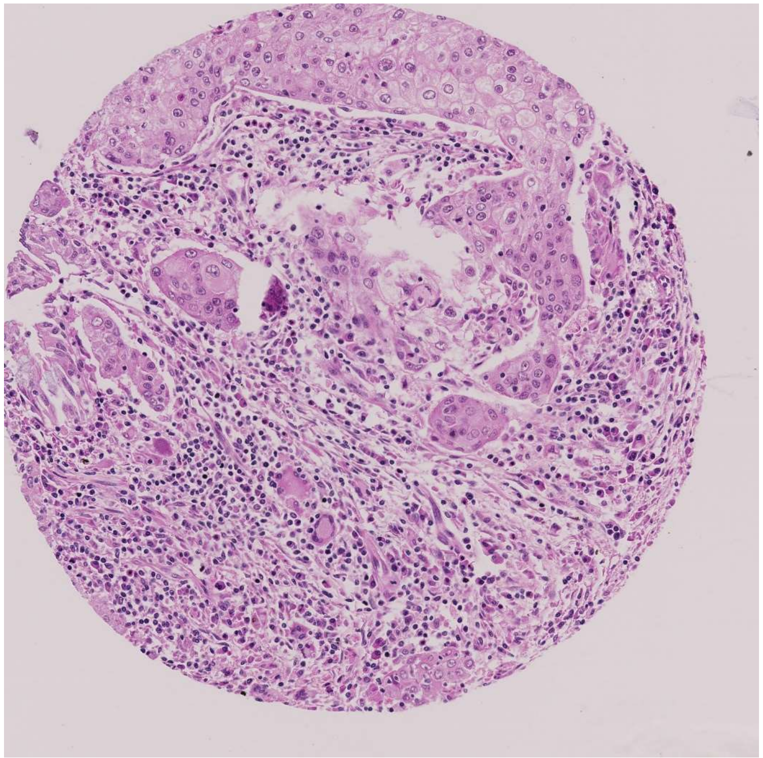

Low 7

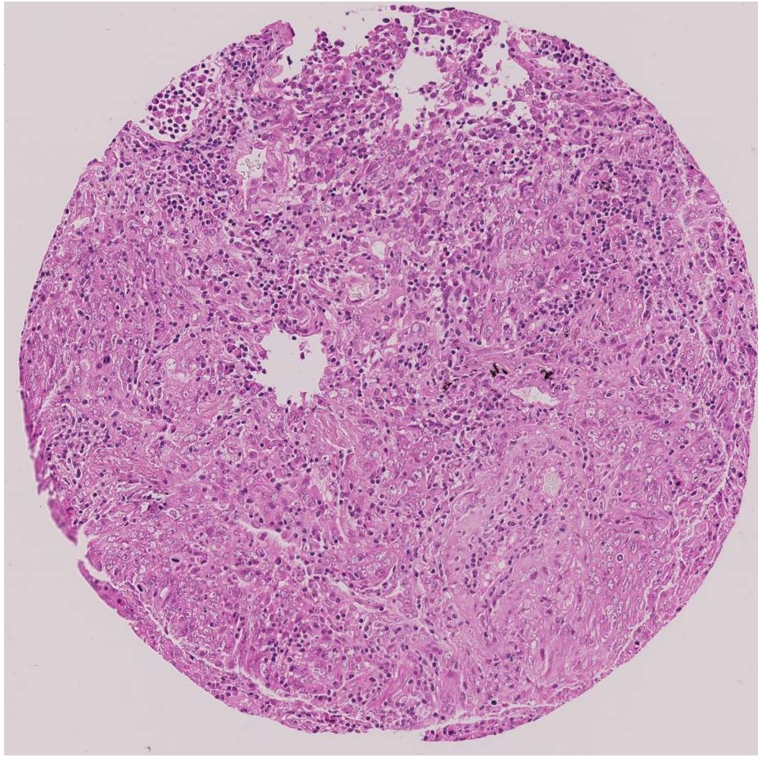

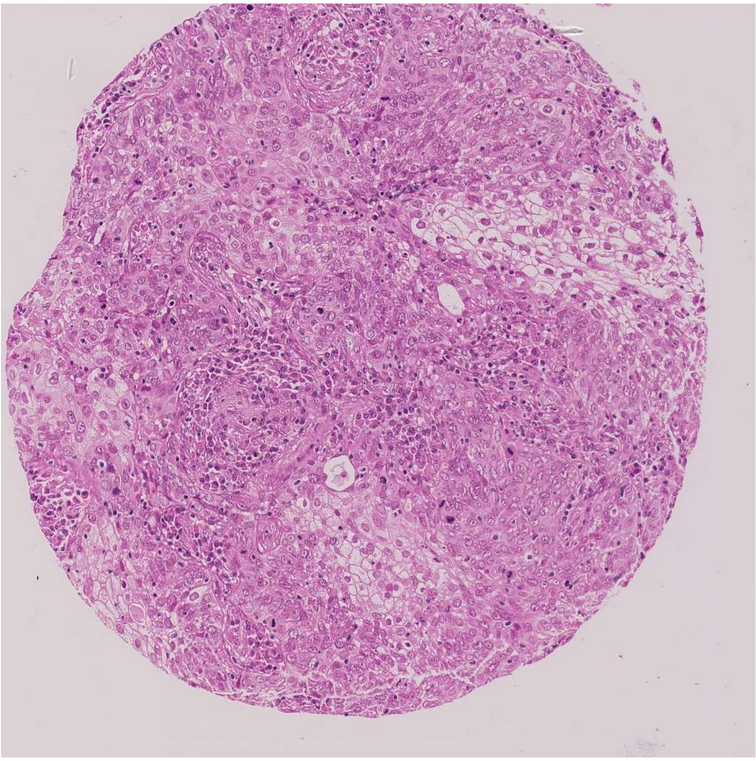

Low 8

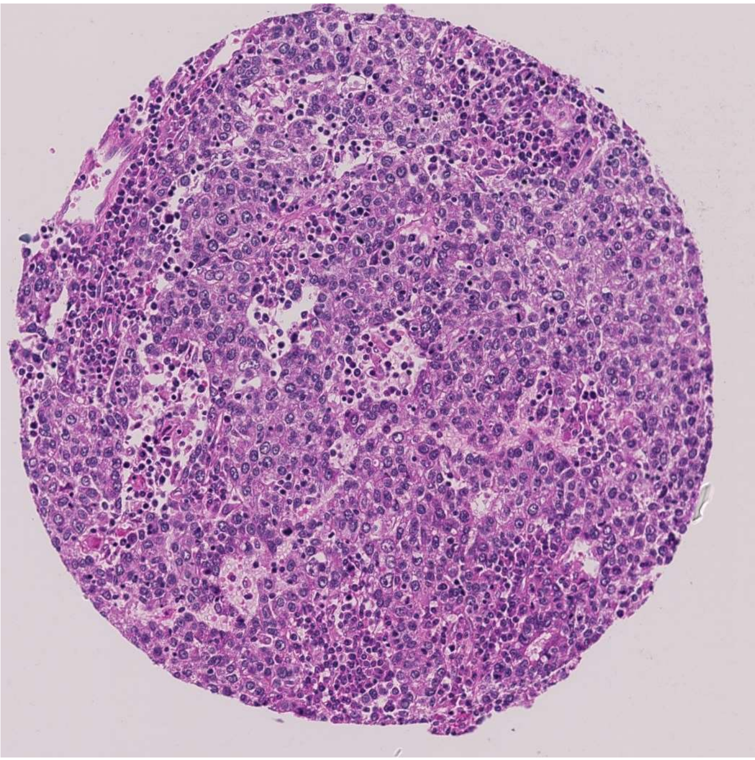

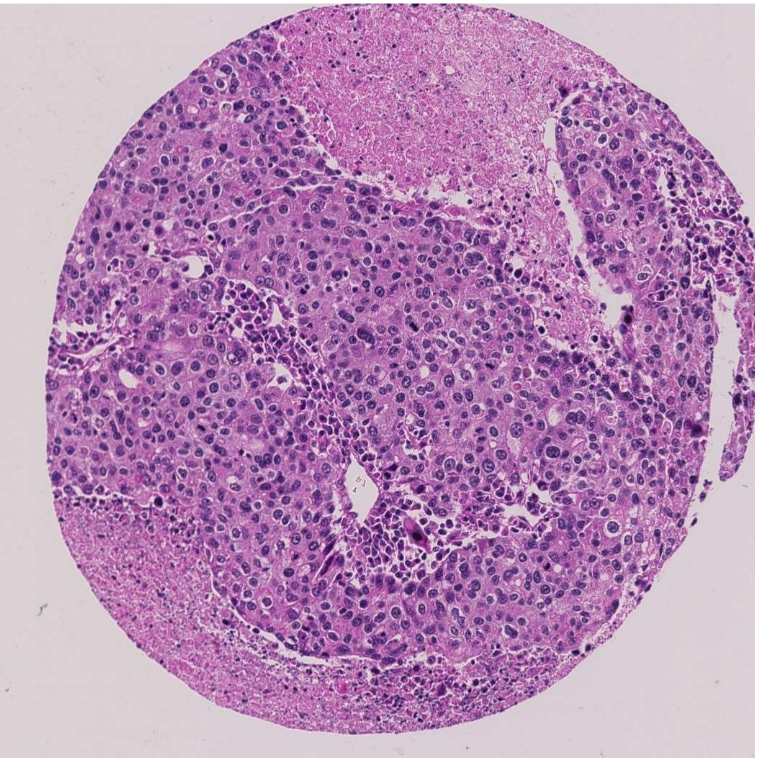

Low 9

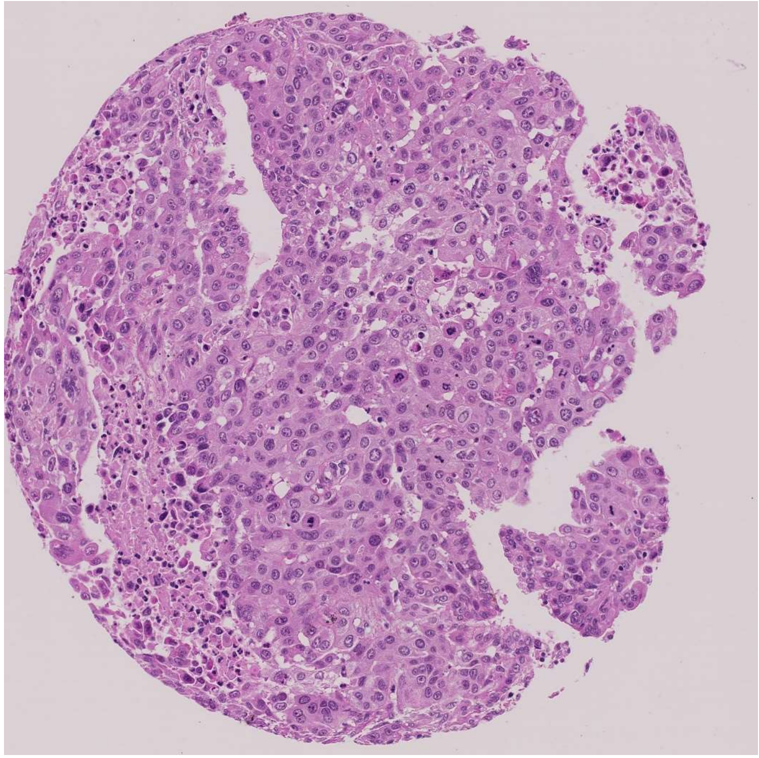

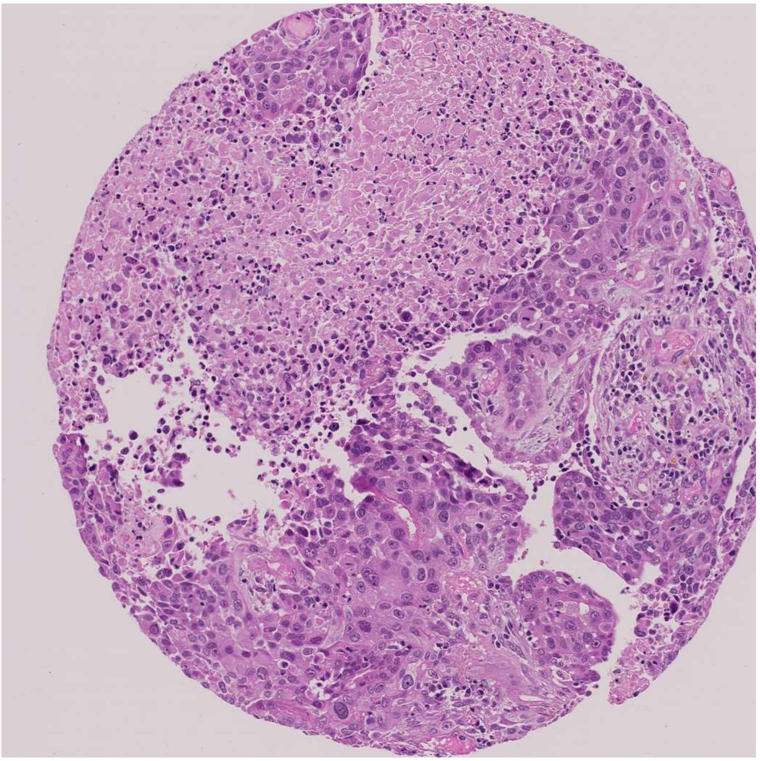

Low 10

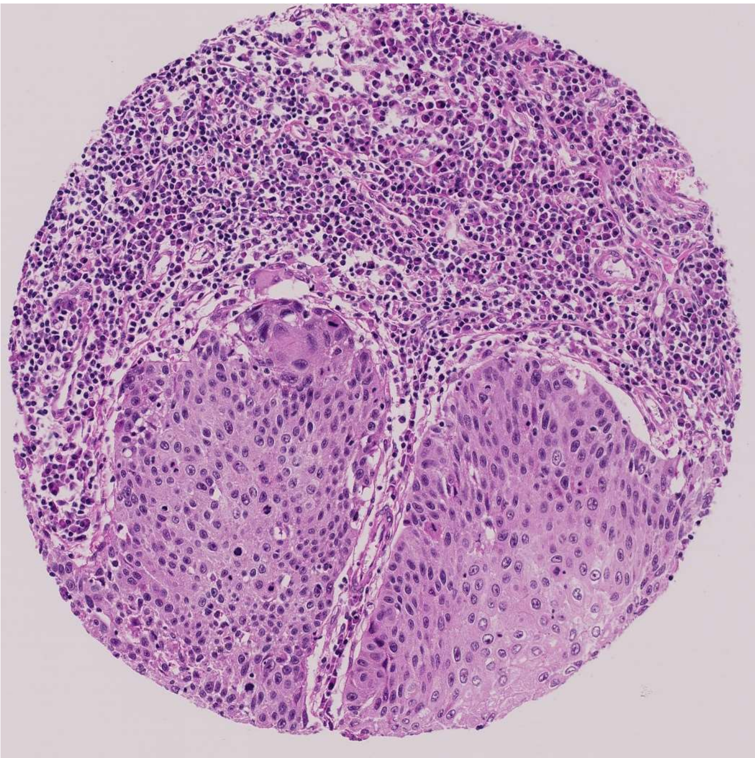

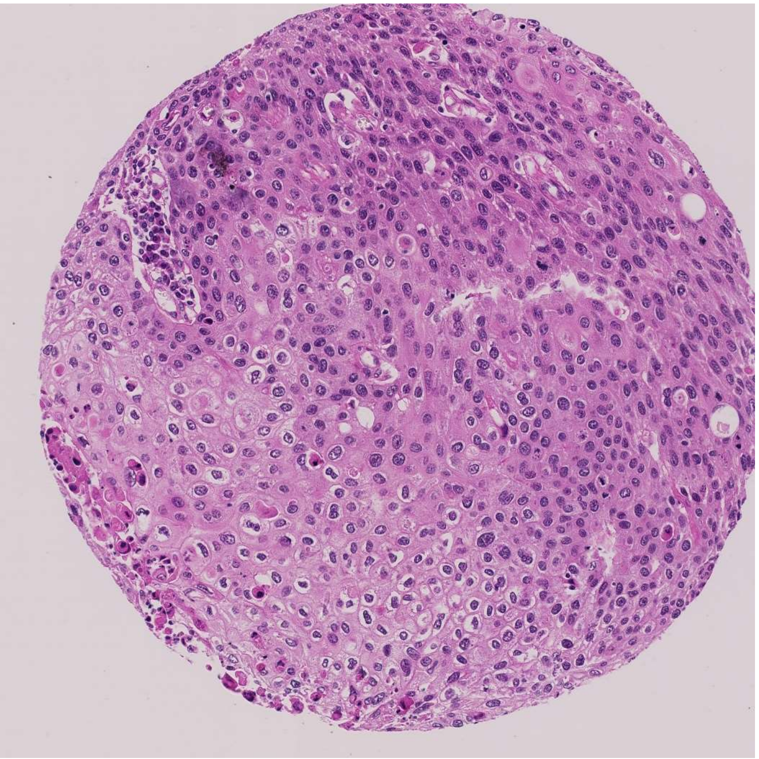

Summary of this feature

- **Length-Width ratio** measures the approximate length-to-width ratio of an object
- **Stromal Nucleus Mean Length-Width** measures the average of the length-to-width ratio feature across all nuclei in the stroma of an image
- As one might imagine:
  - A stroma rich in lymphocytes (with relatively round nuclei) would have a **lower** Mean
  - A stroma rich in spindle cells (with relatively elongated nuclei) would have a **higher** Mean
- Consistent with this, the highest 10 cases have relatively less **stromal chronic inflammation** than the lowest 10 by manual scoring (see next slide)

Inflammation

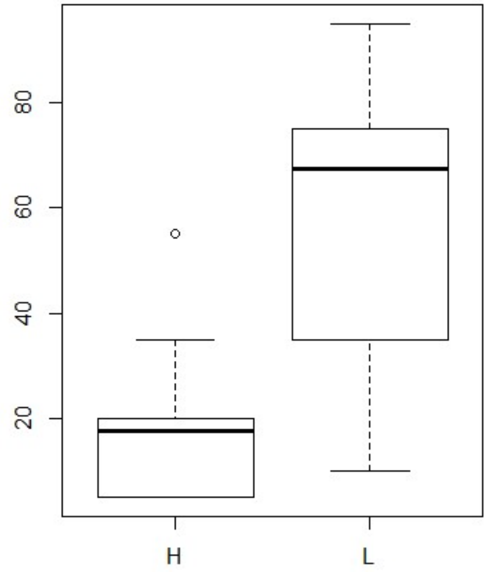

- H = highest 10
- L = lowest 10
- P = 0.000884

Survival

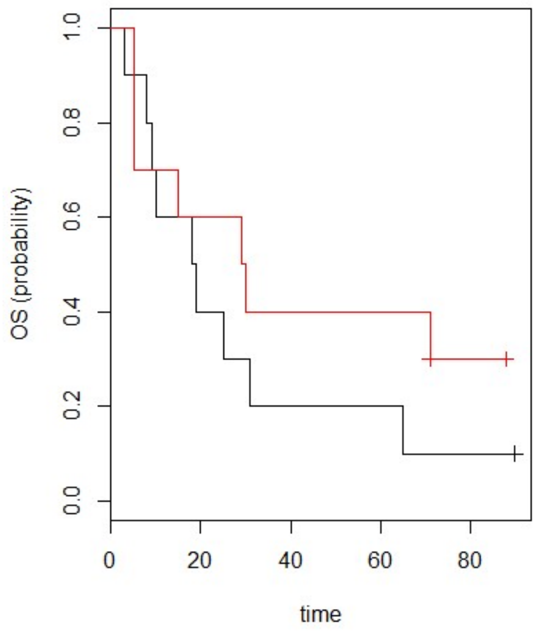

- Red = lowest 10
- Black = highest 10
- P = 0.301

Stroma.Nucleus.Max.Shape.index

What is Stroma.Nucleus.Max.Shape.index?

The Shape index describes the smoothness of an image object border. The smoother the border of an image object is, the lower its shape index. It is calculated from the Border Length feature of the image object divided by four times the square root of its area.

Parameters

- b_v is the image object border length
- $4\sqrt{\#P_v}$ is the border of square with area $\#P_v$

Expression

$$\frac{b_v}{4\sqrt{\#P_v}}$$

Feature Value Range

$[1, \infty]$; 1 = ideal.

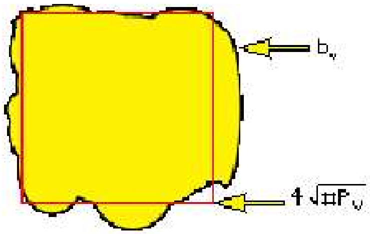

Figure 24.17. Shape index of an image object v

High 1

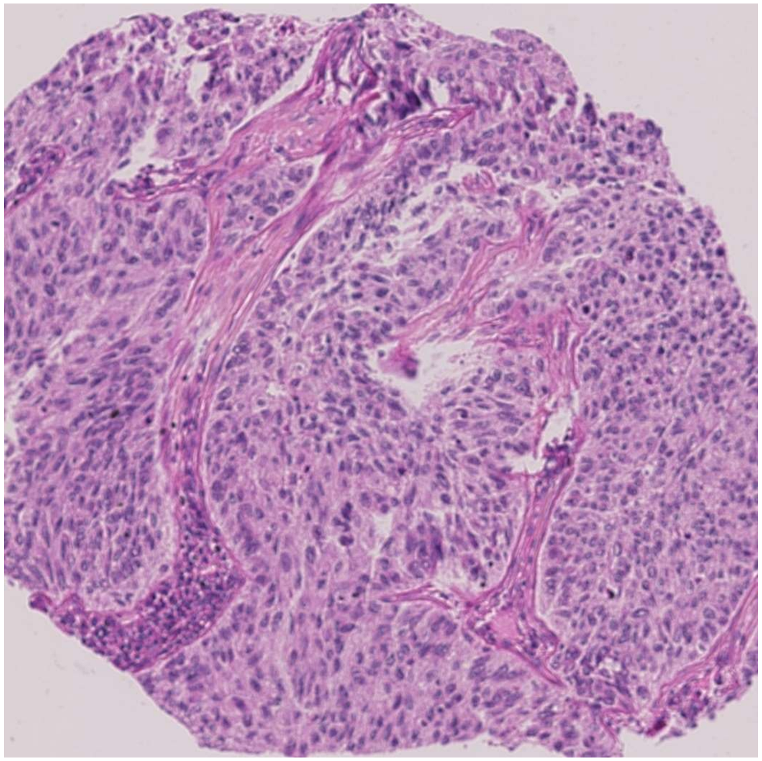

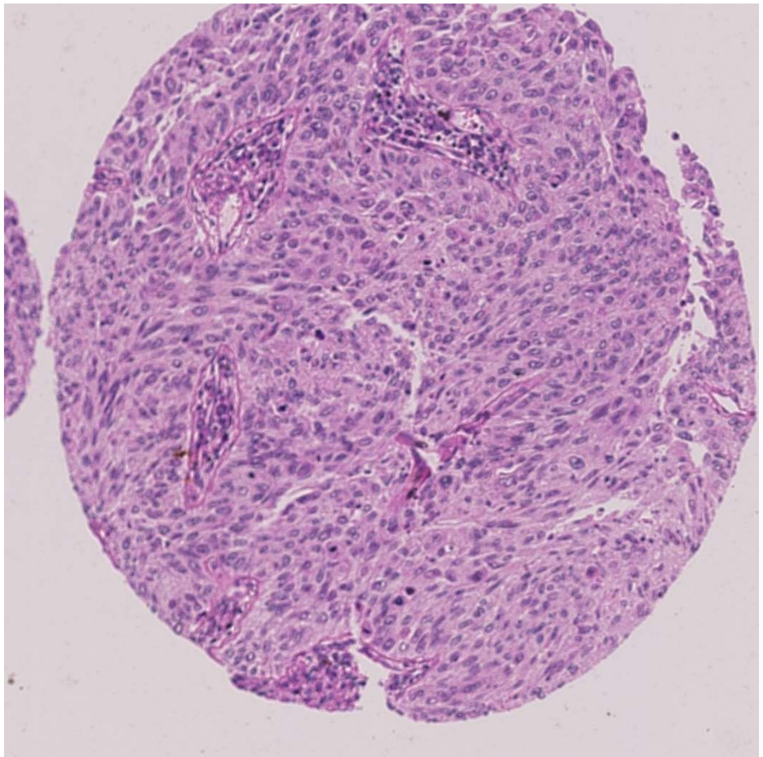

High 2

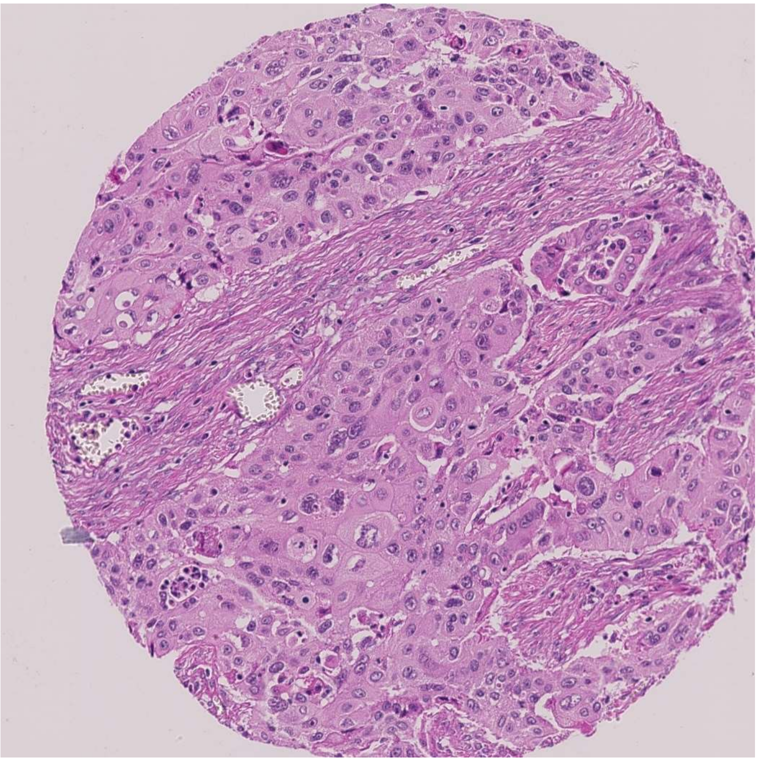

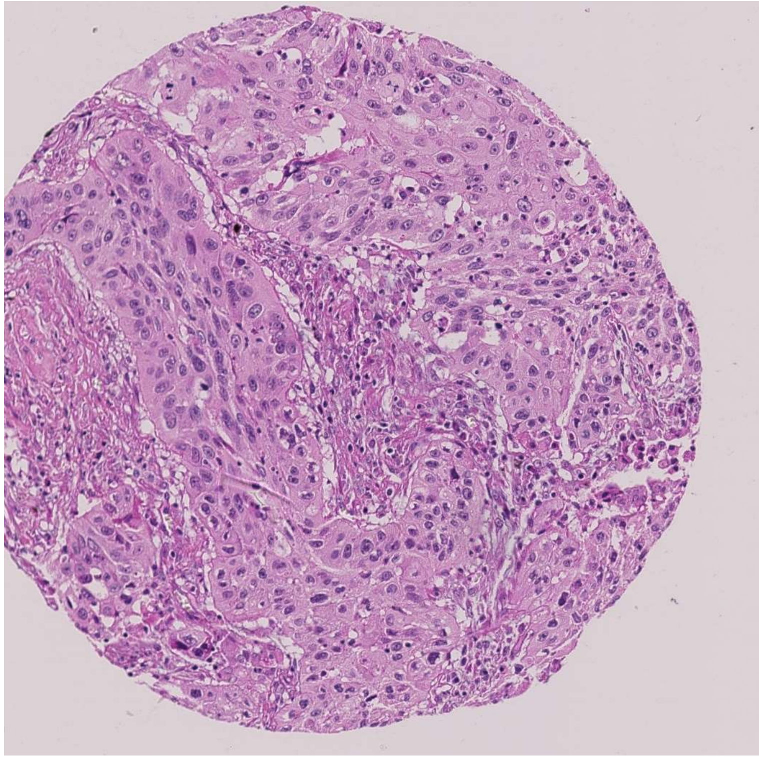

High 3

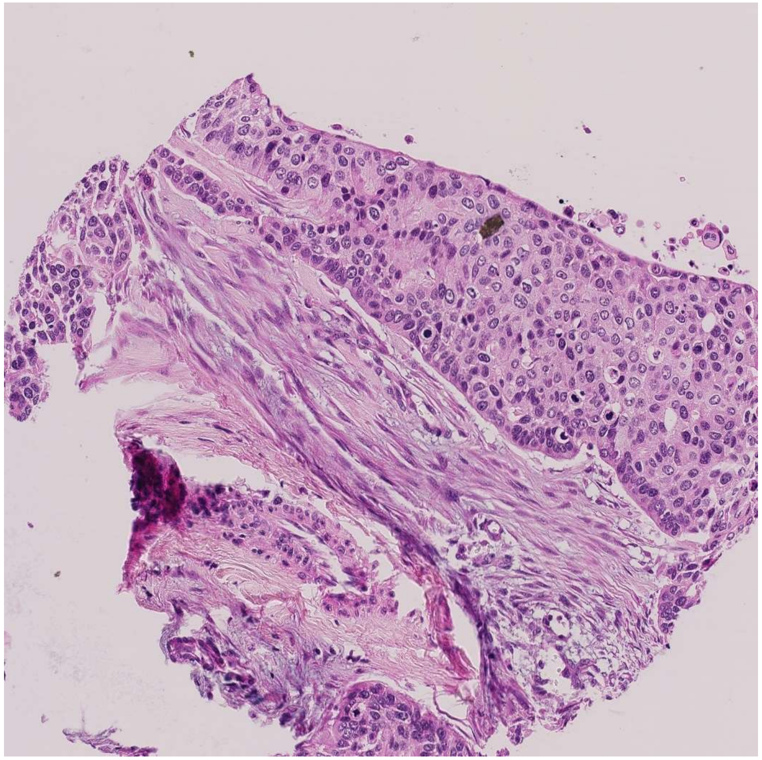

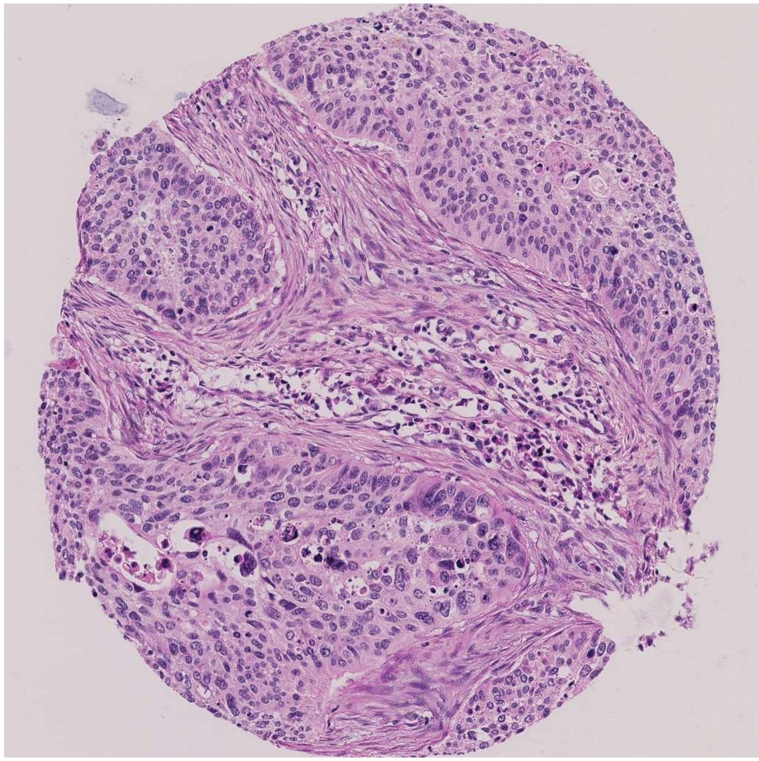

High 4

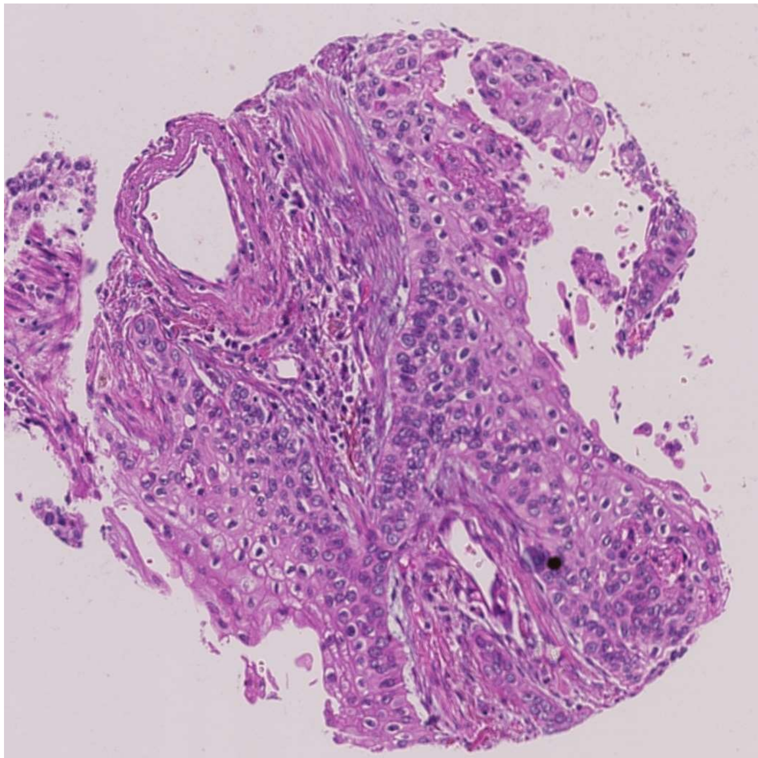

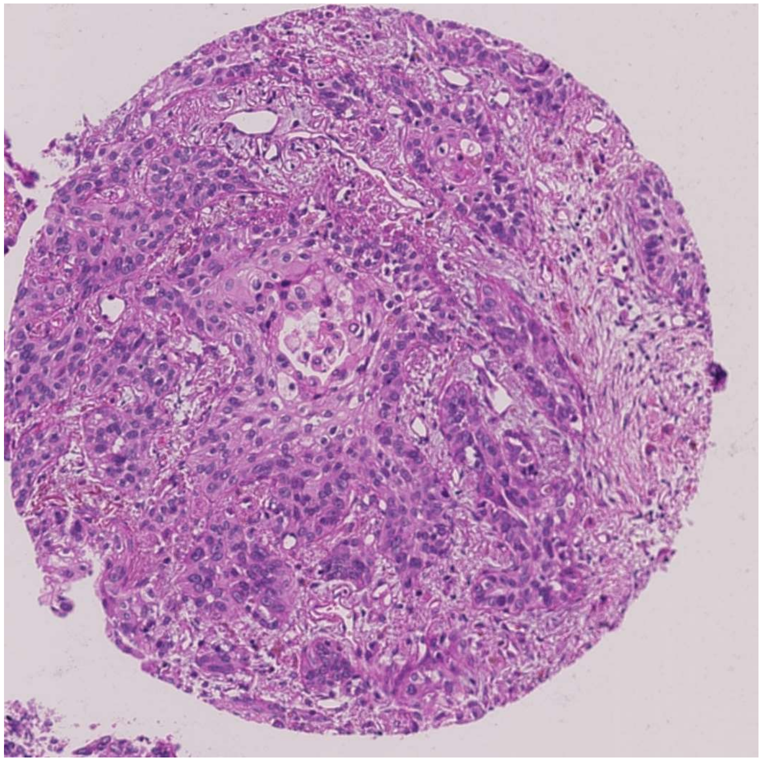

High 5

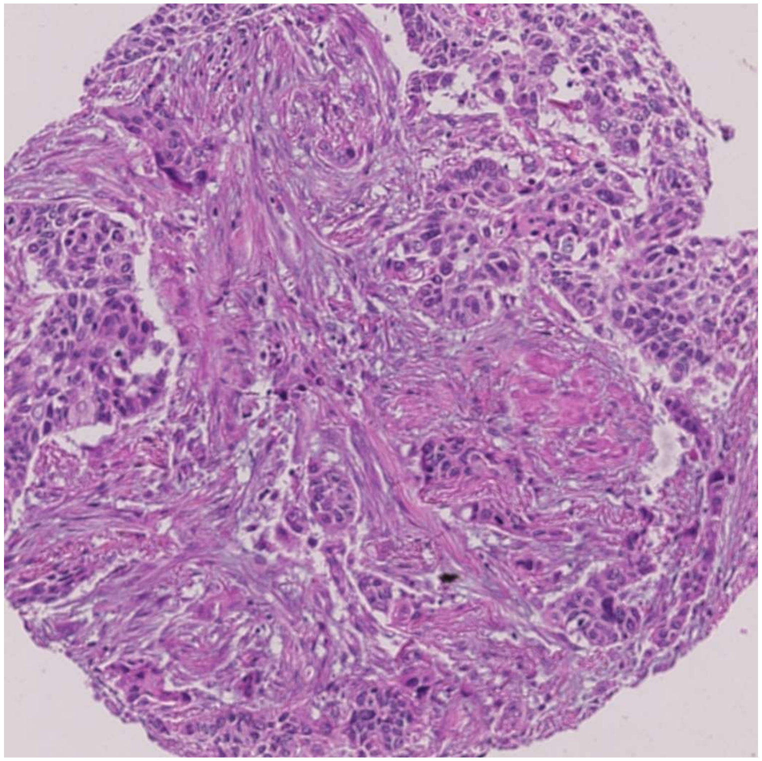

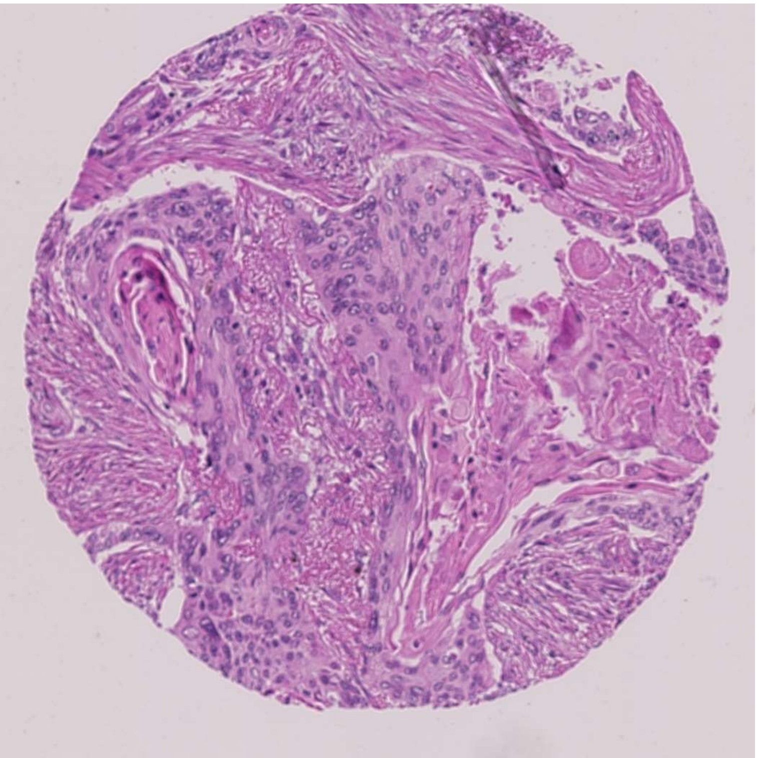

High 6

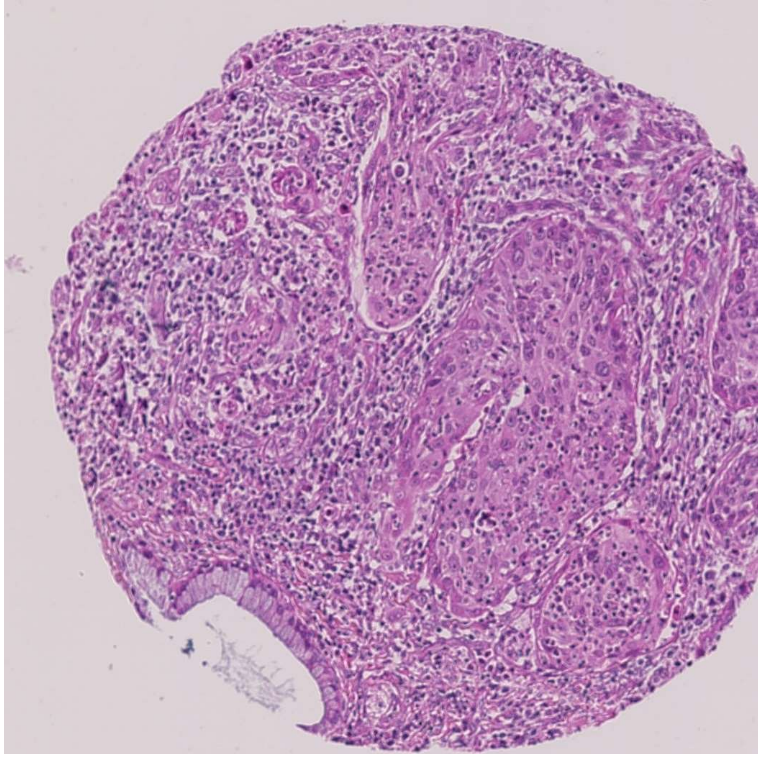

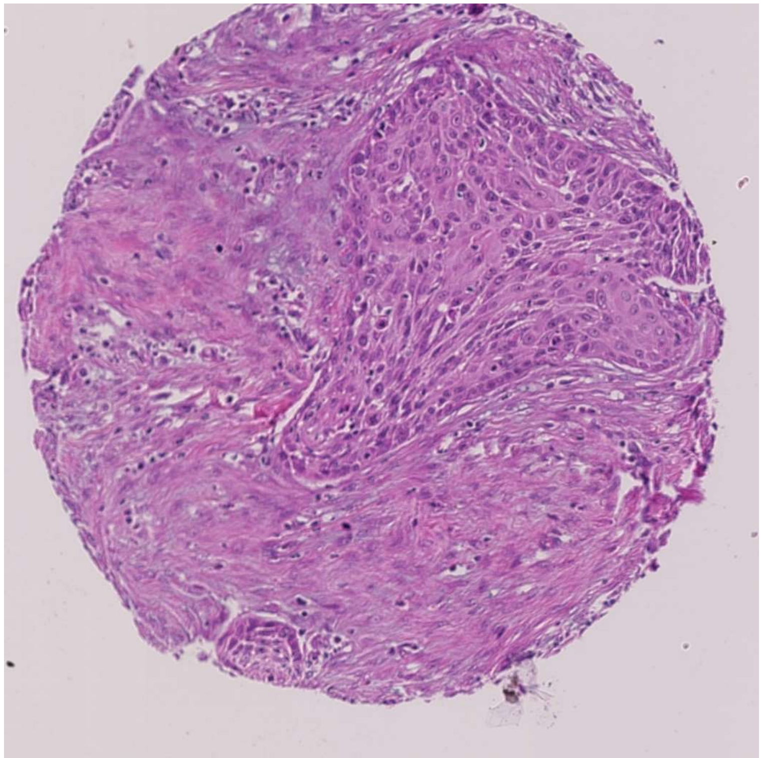

High 7

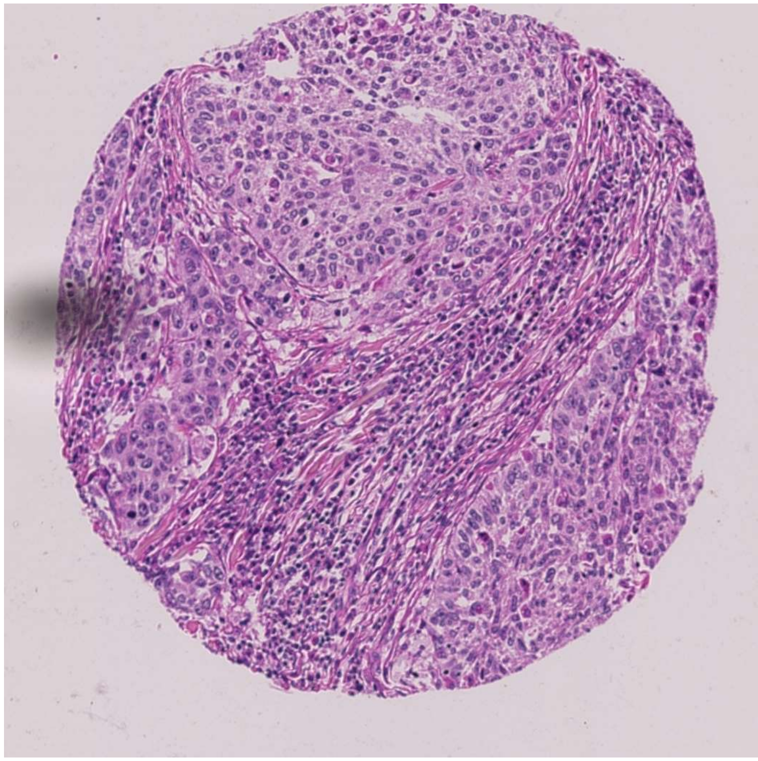

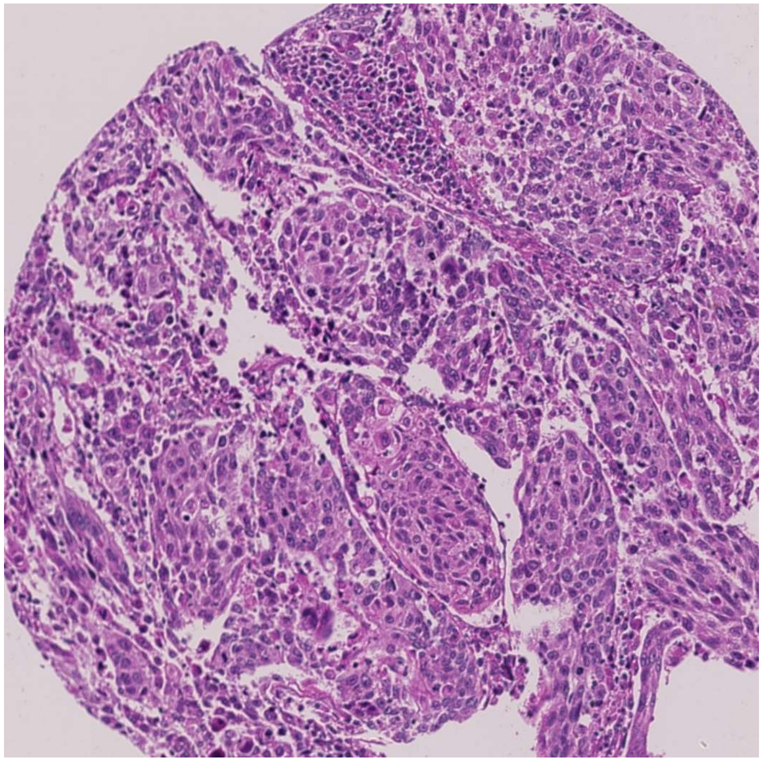

High 8

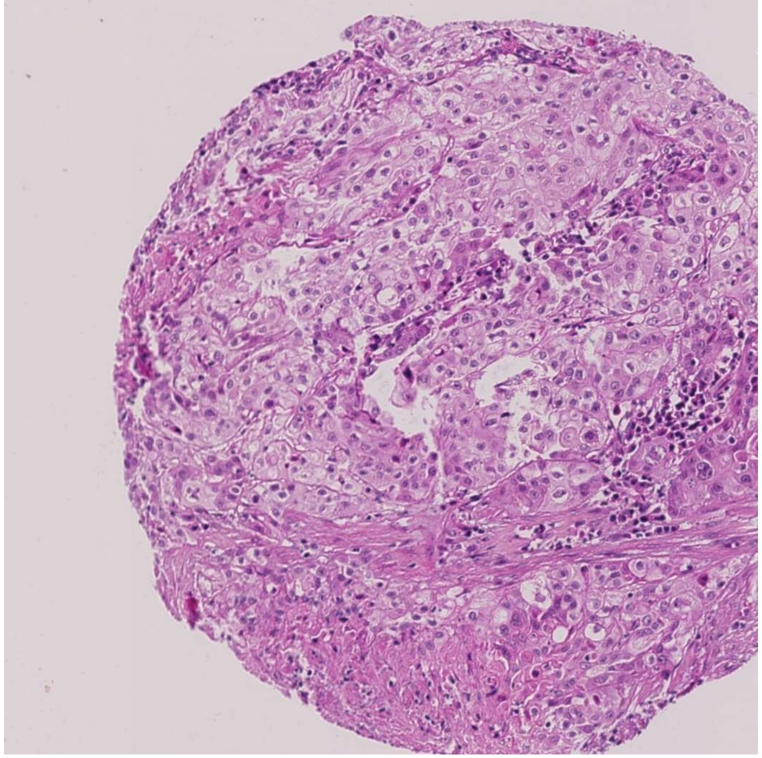

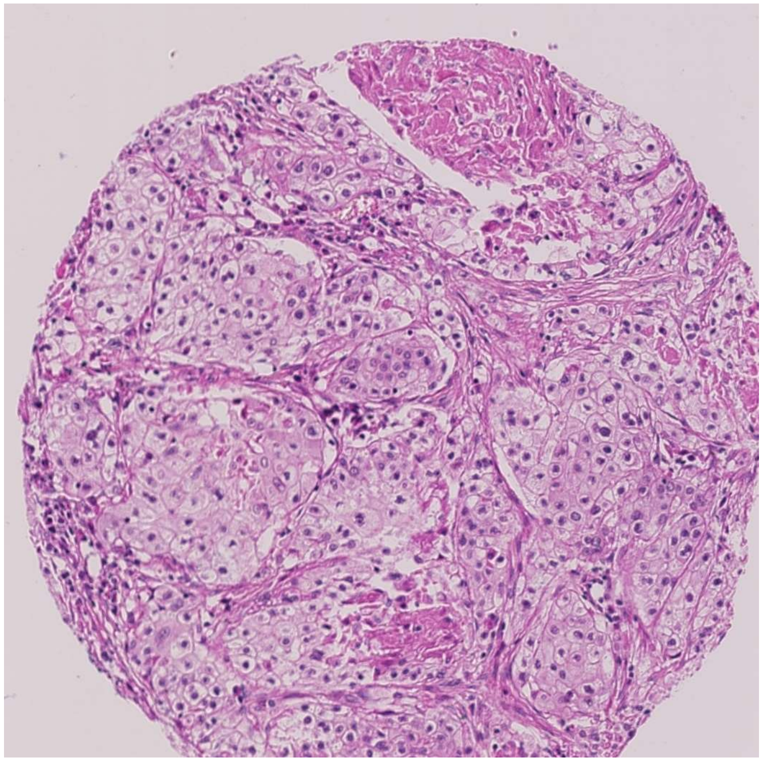

High 9

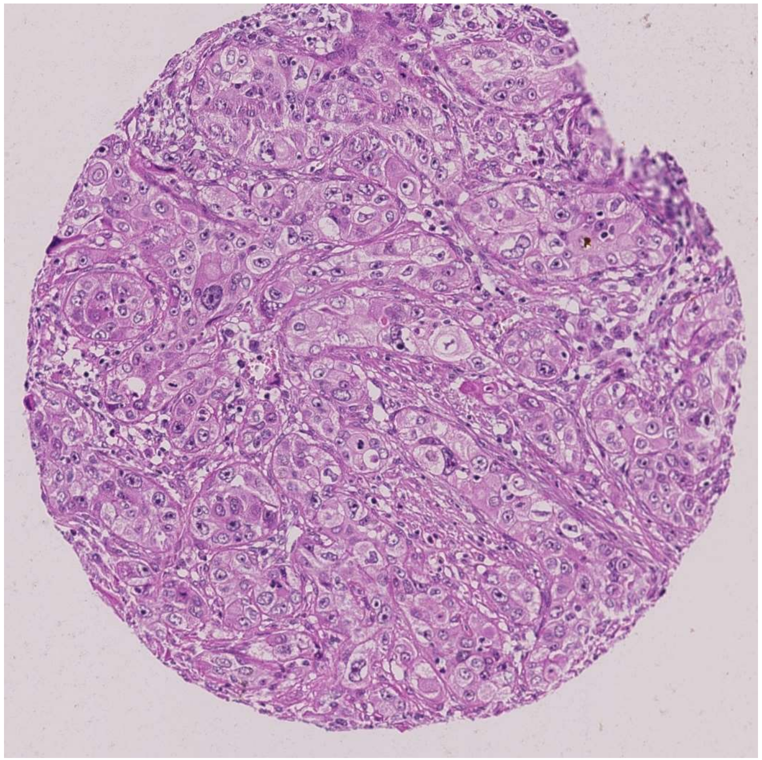

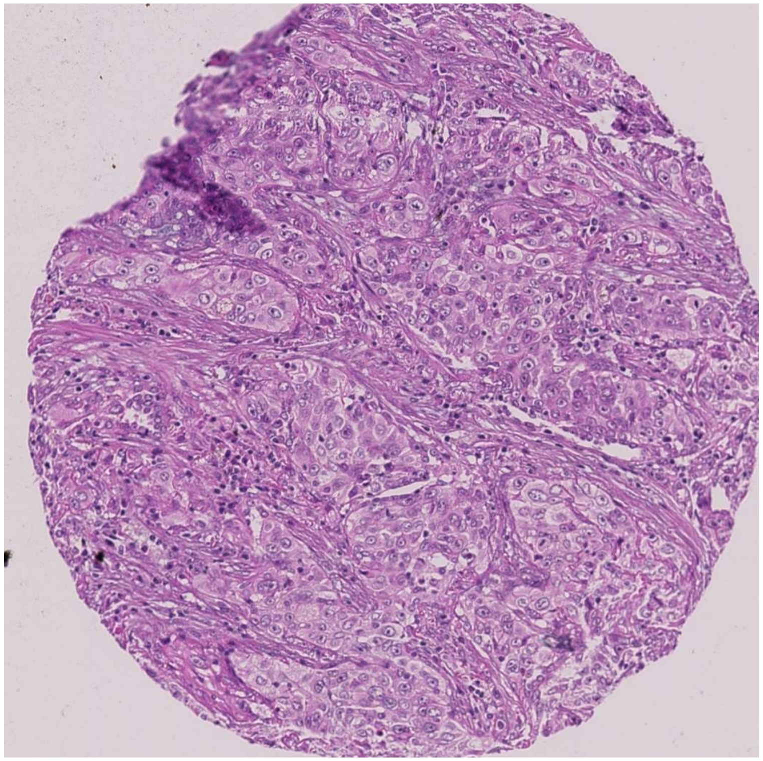

High 10

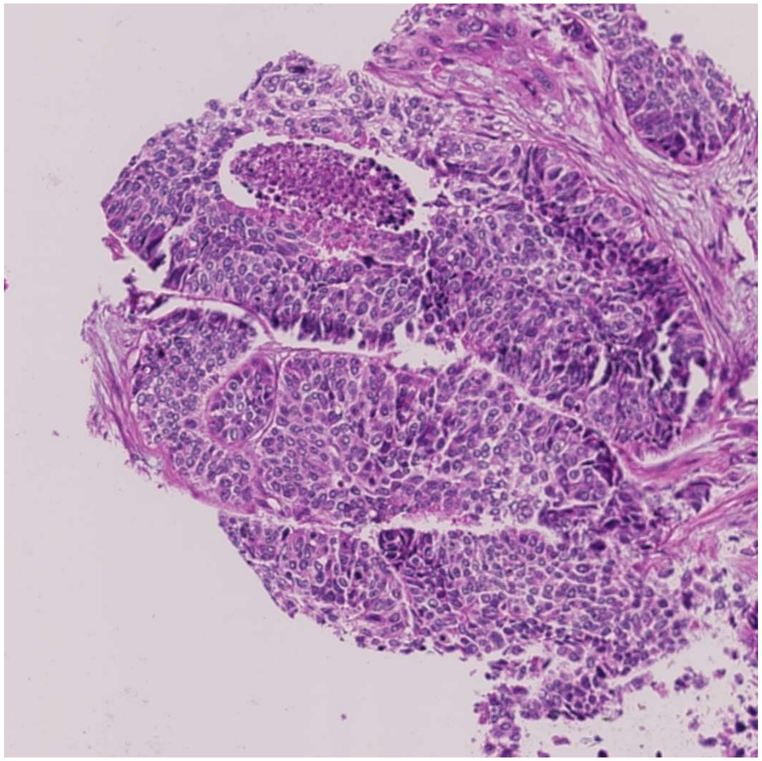

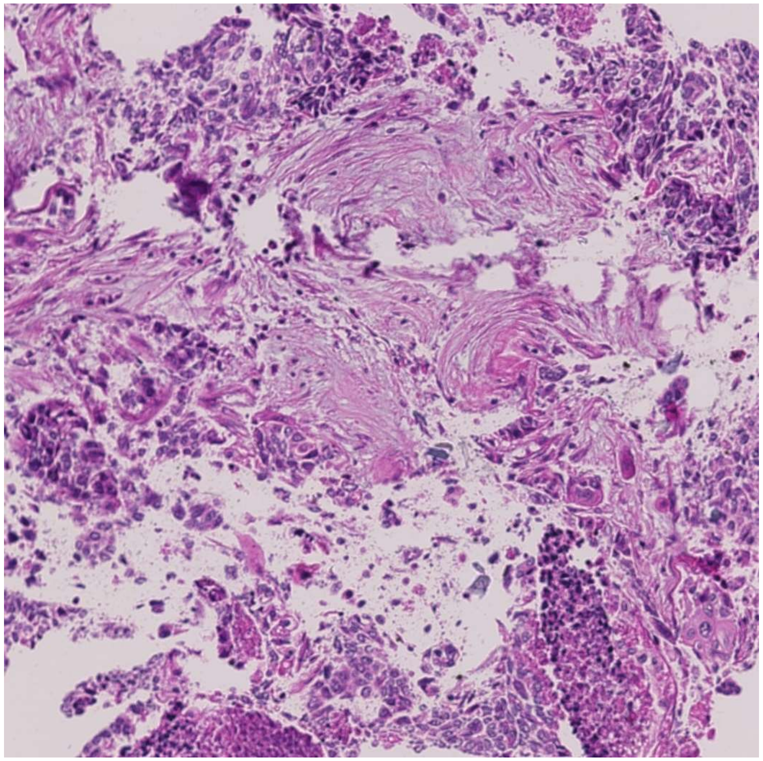

Low 1

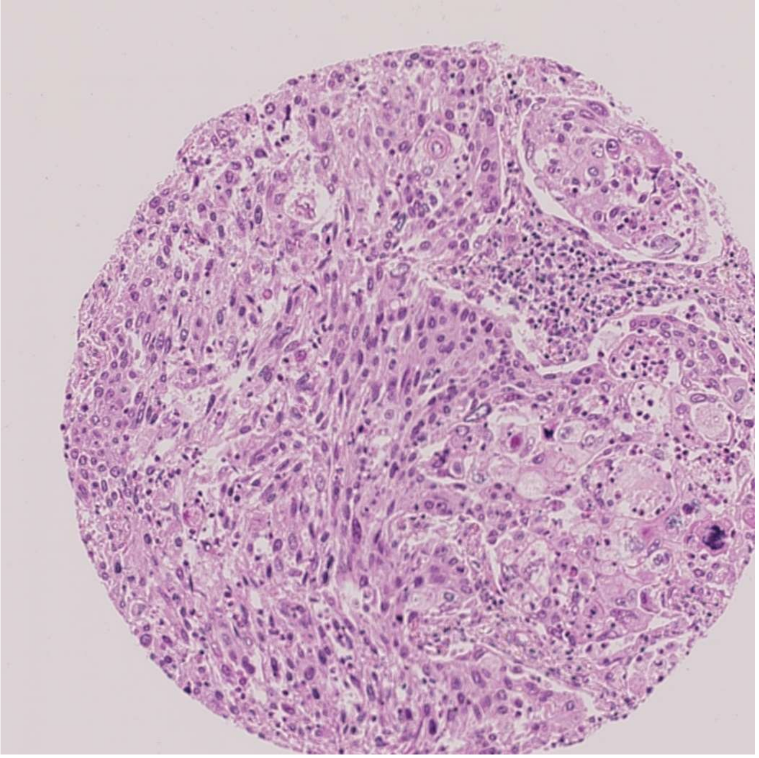

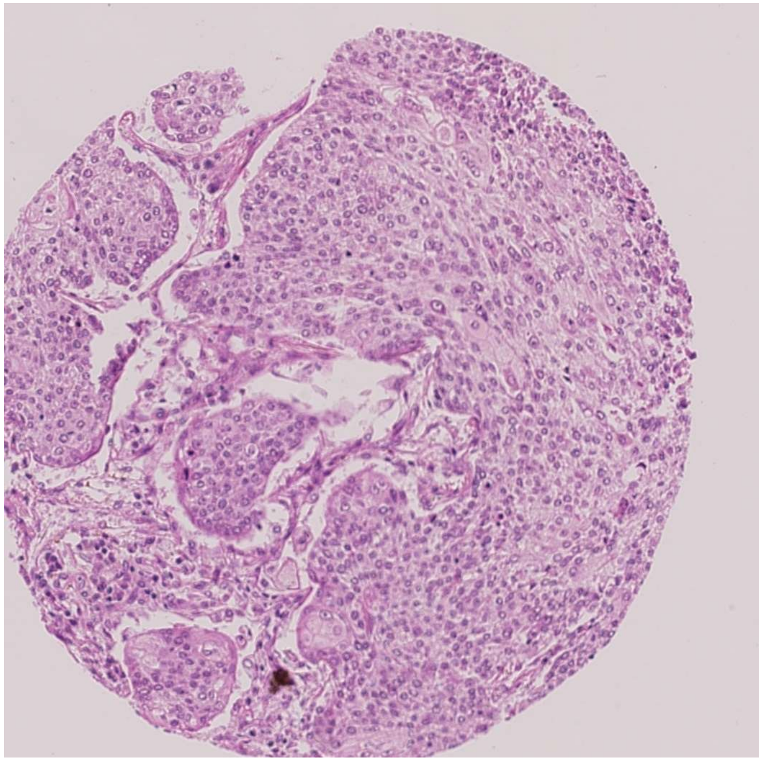

Low 2

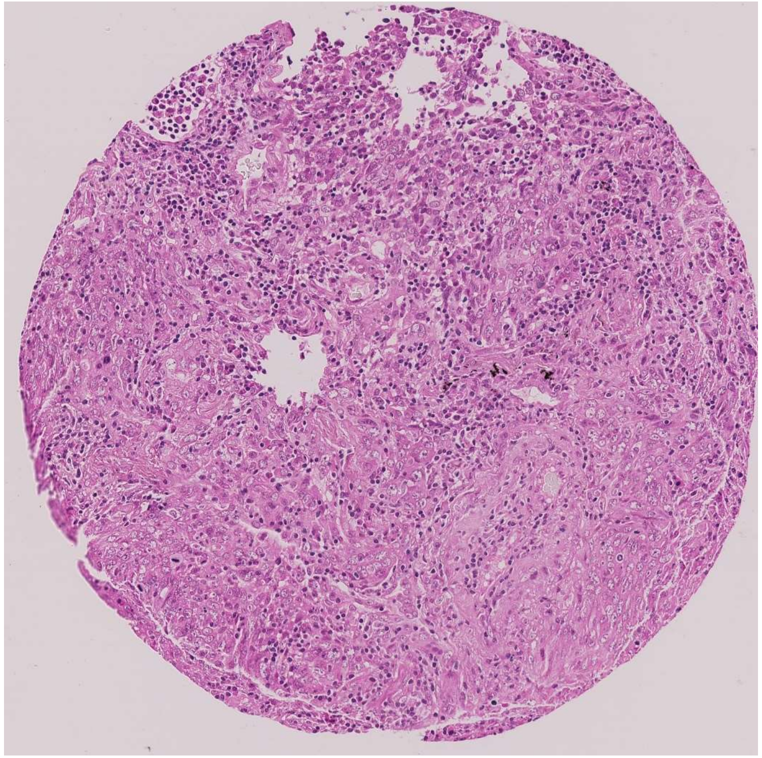

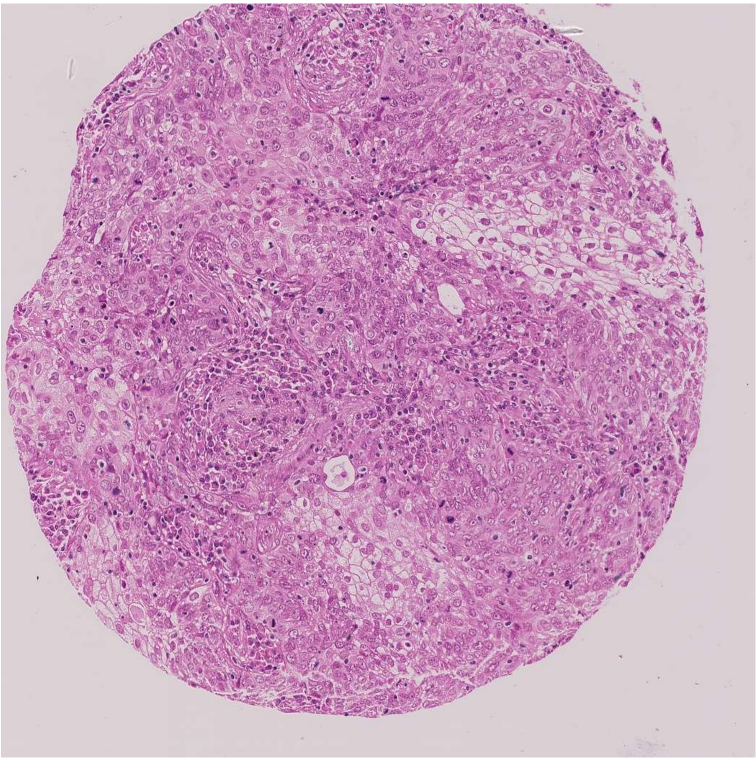

Low 3

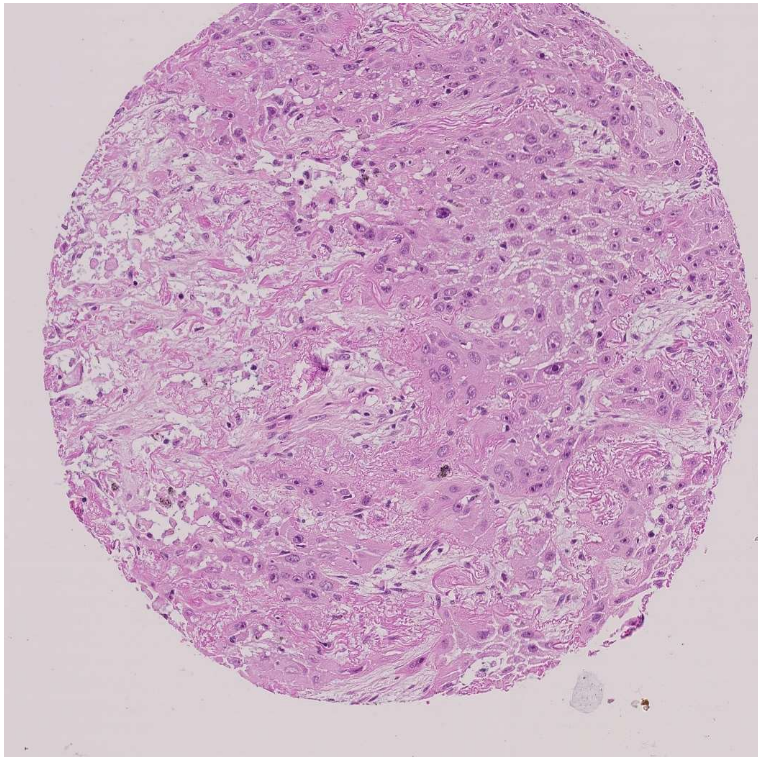

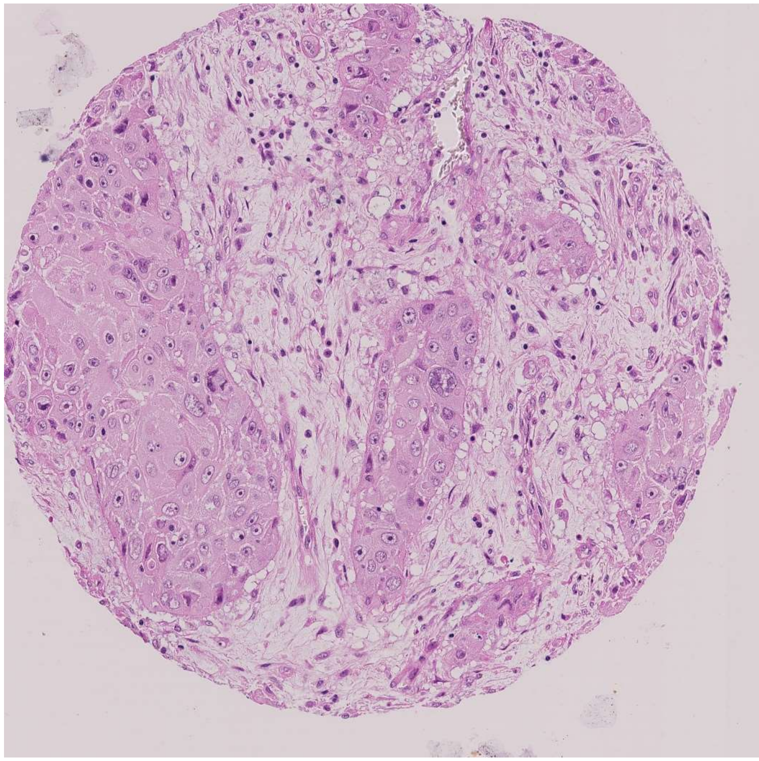

Low 4

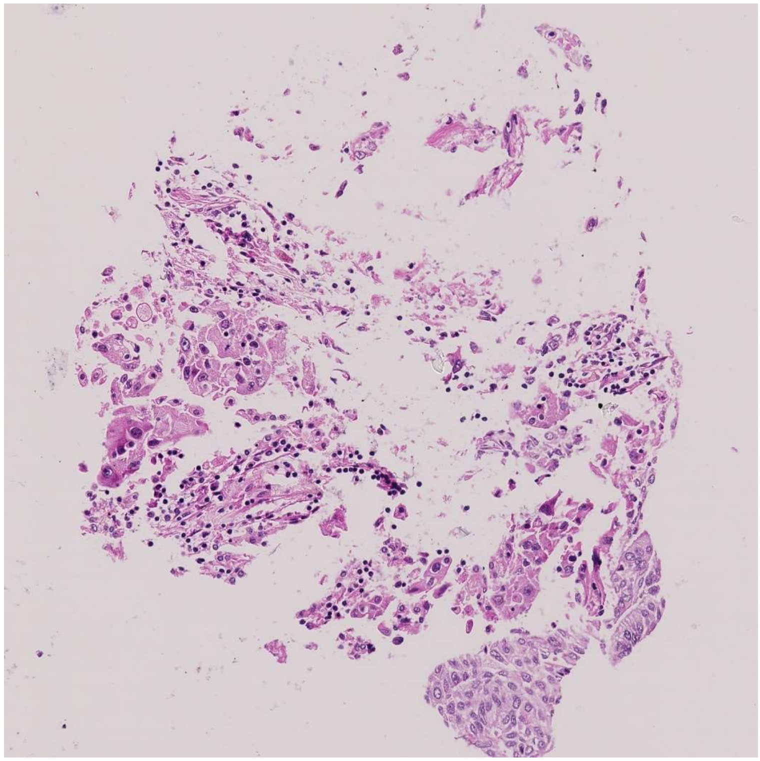

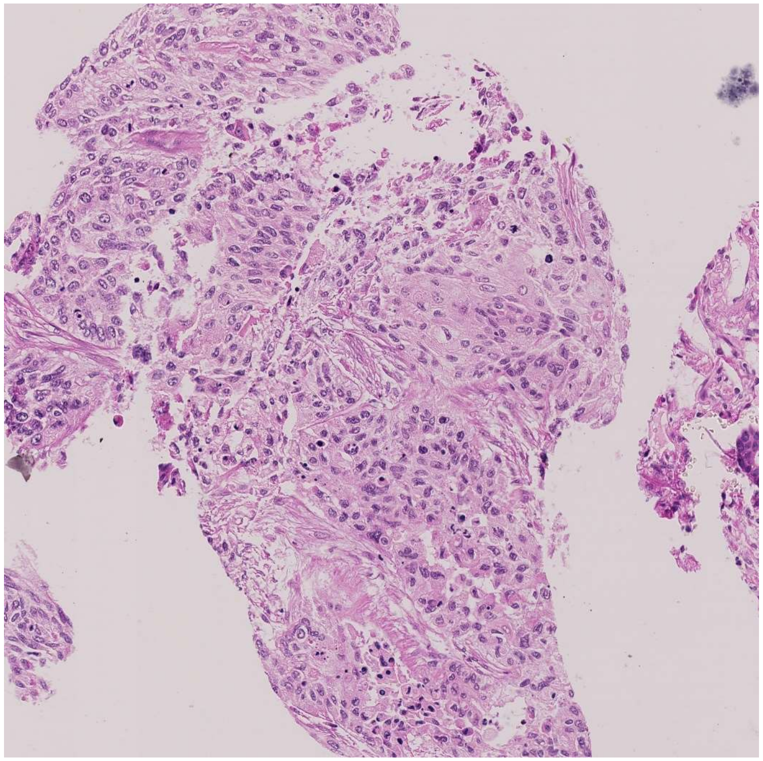

Low 5

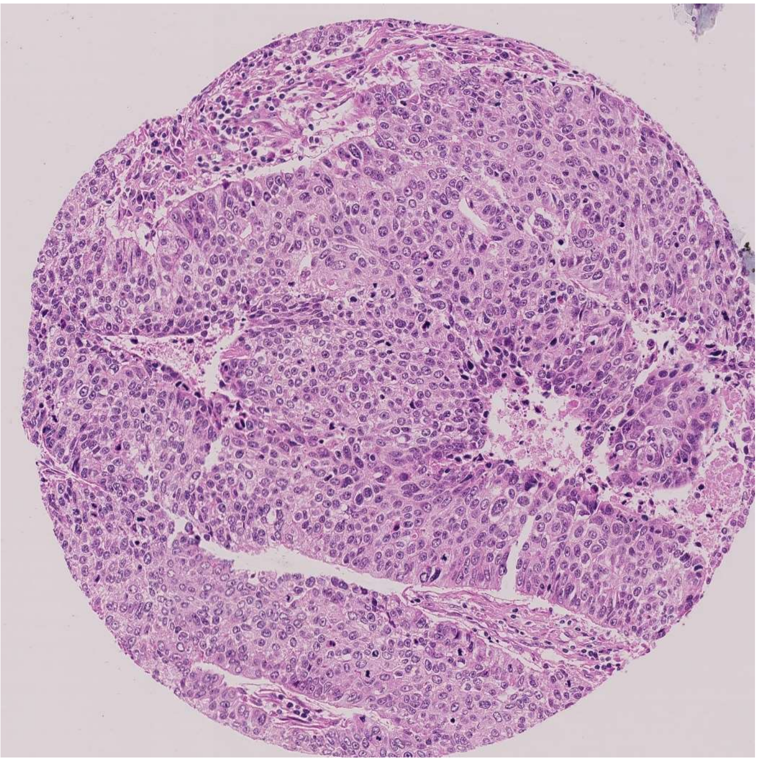

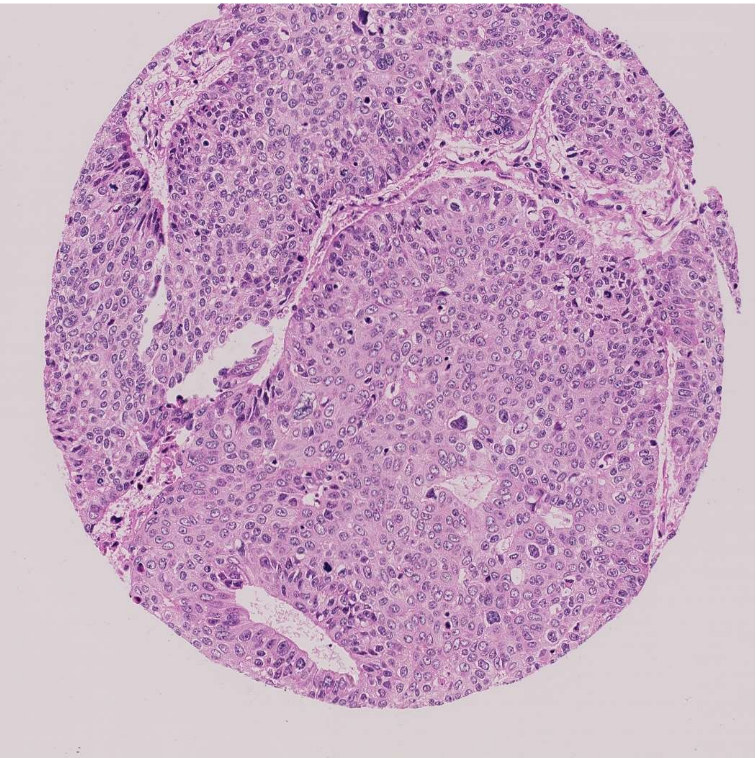

Low 6

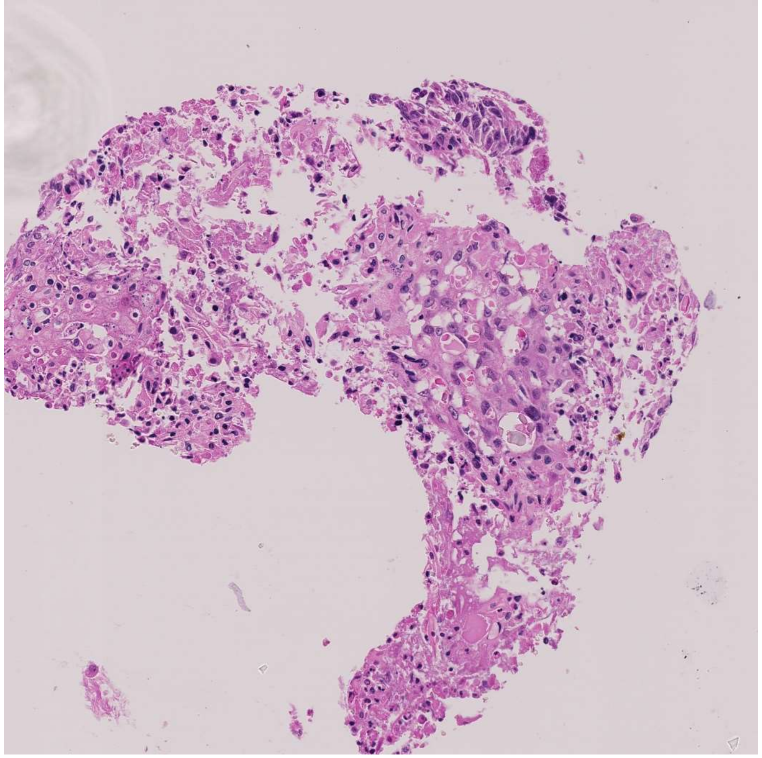

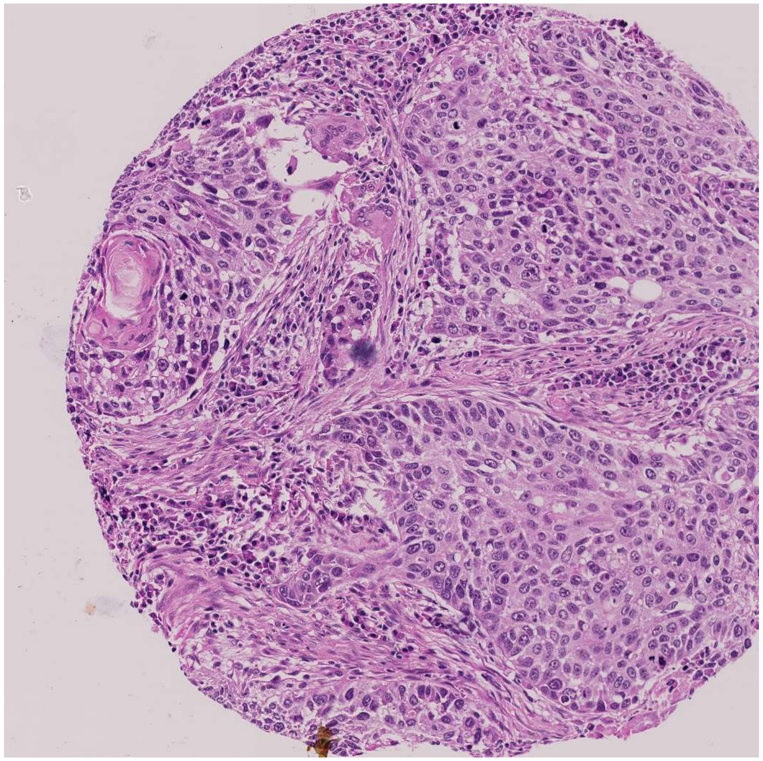

Low 7

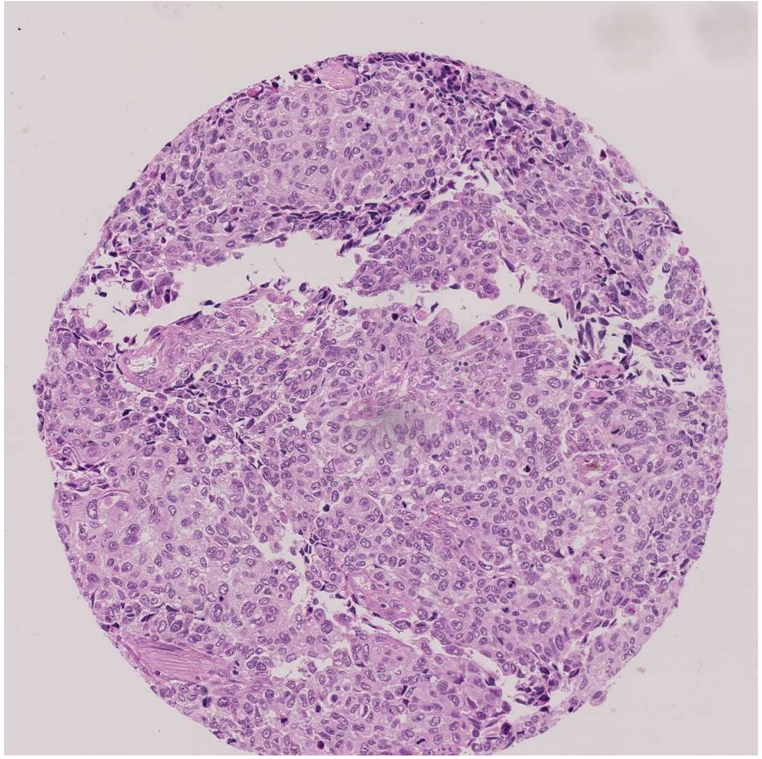

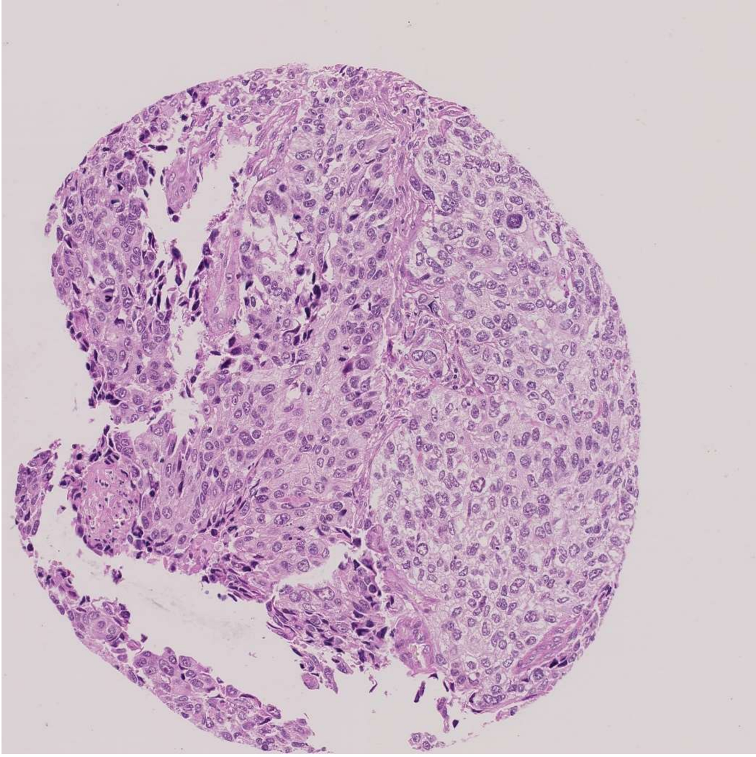

Low 8

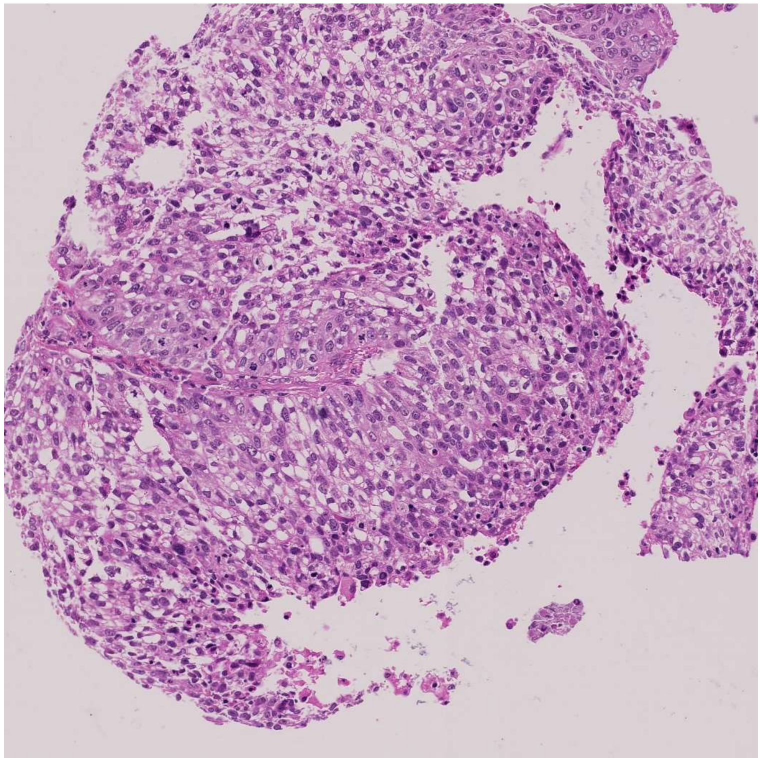

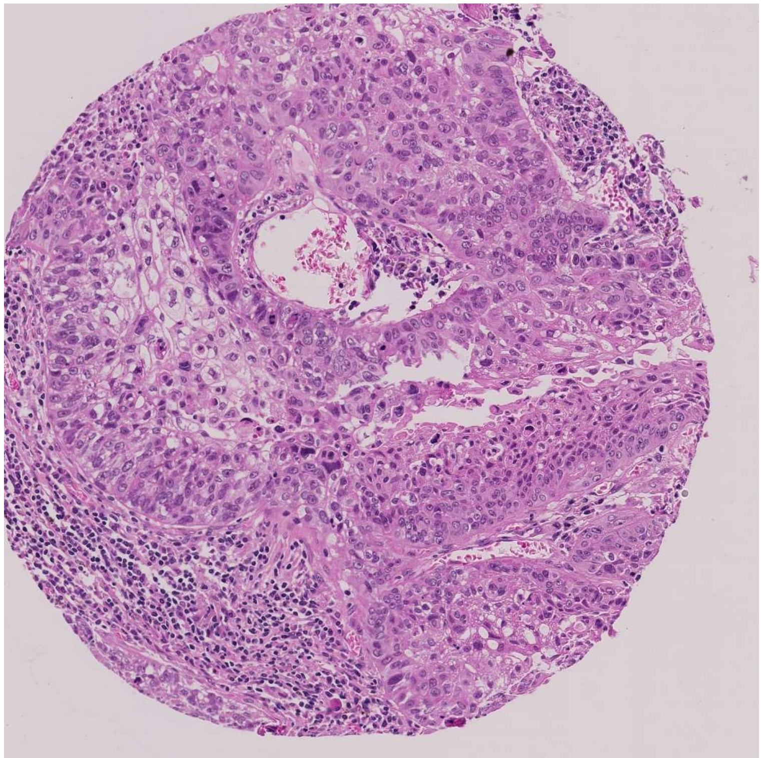

Low 9

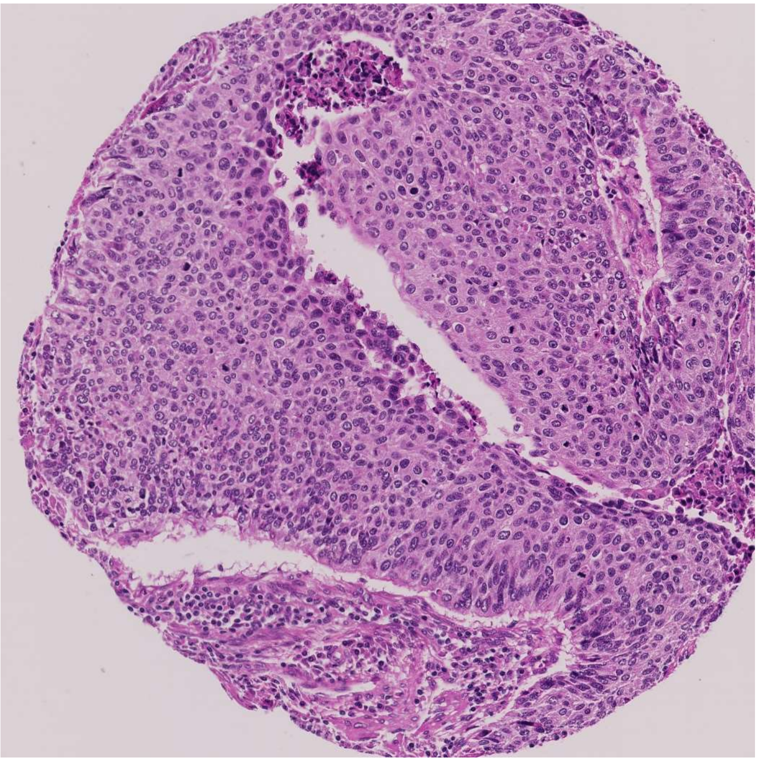

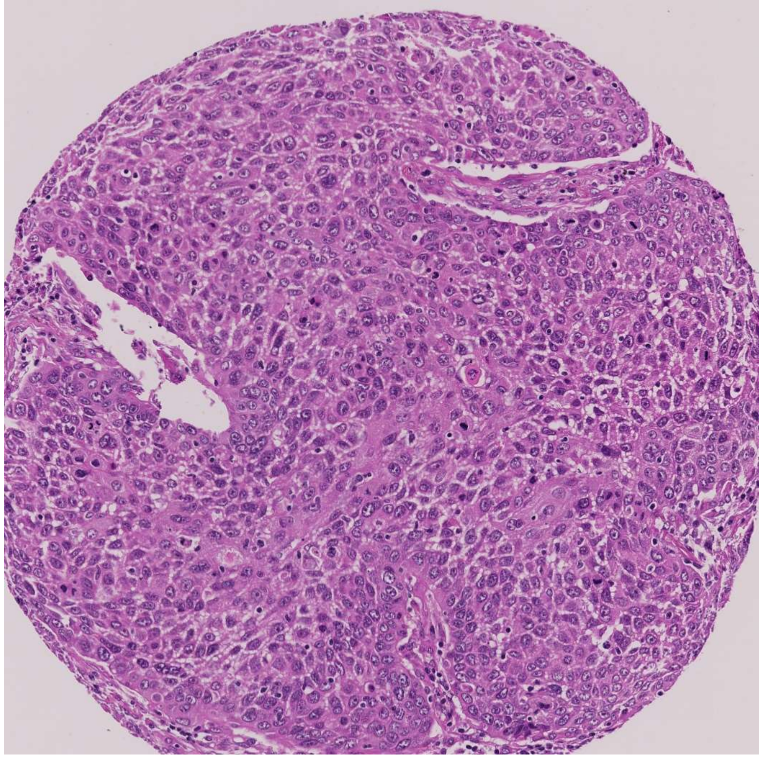

Low 10

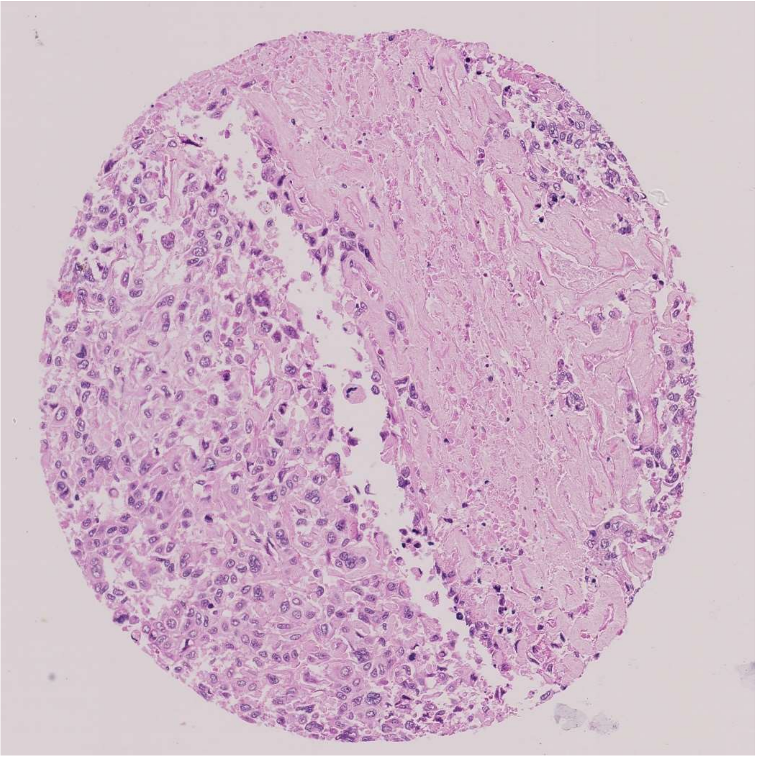

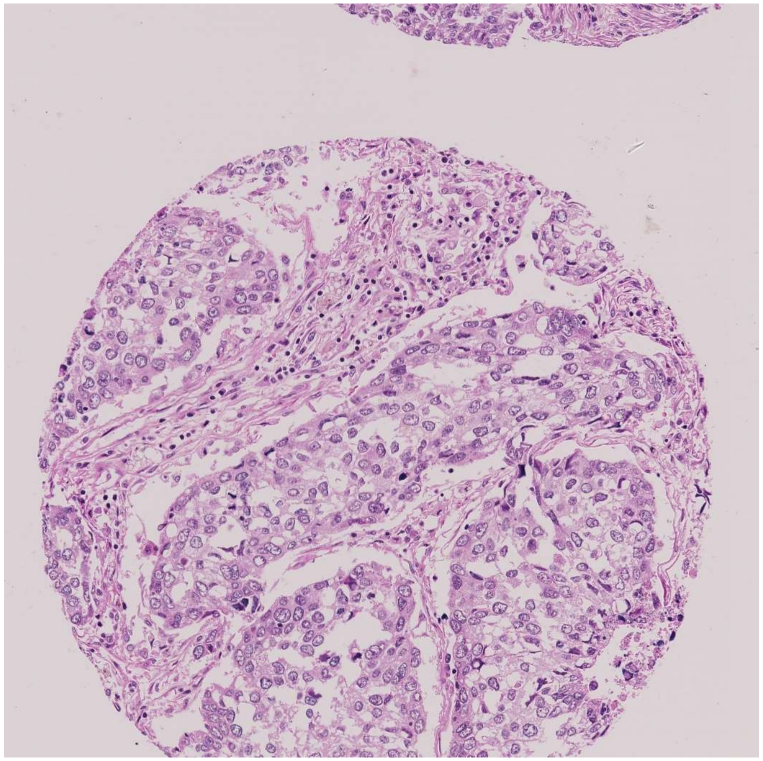

Summary of this feature

- **Shape index** measures the border length divided by four times the square root of the area of an object, and is a measure of the smoothness of an object
- **Max Stromal Nucleus Shape Index:** the shape indices of all nuclei in the stroma are measured individually during the analysis of an image; however, this feature only records the highest shape index value belonging to a single stromal nucleus
- As one might imagine:
  - If this single nucleus belongs to a lymphocyte, the shape of the nucleus would be relatively smooth and the maximum stromal nucleus shape index for the image would be **low**
  - If this single nucleus belongs to a stromal cell, the shape of the nucleus would be relatively irregular and the maximum stromal nucleus shape index for the image would be **high**
- **However**, visual inspection of the highest 10 and lowest 10 cases did not reveal an obvious difference in the stromal quality of the extreme cases for this feature; furthermore, there does not appear to be a difference in stromal inflammation between the highest 10 and lowest 10 cases (see next slide)
- **I have been unable to translate this feature into a histologic criteria for manual scoring**

Inflammation (manual scoring)

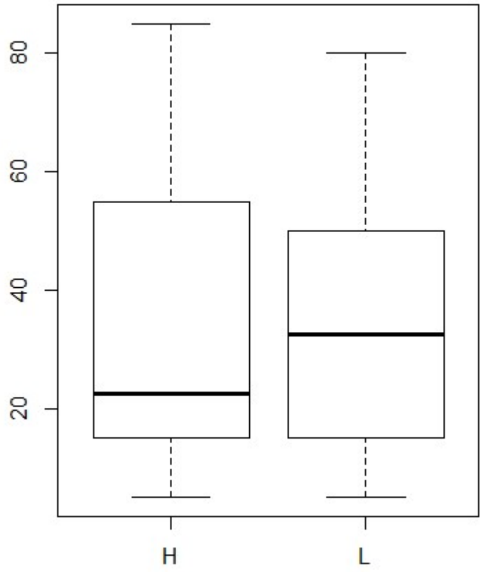

- H = highest 10
- L = lowest 10
- P = 0.693

Survival

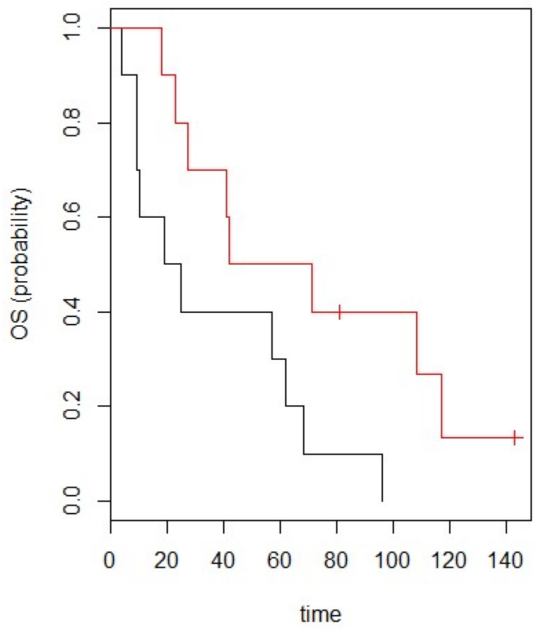

- Red = Lowest 10
- Black = Highest 10
- P = 0.035
